# Circadian Clocks Are Resounding in Peripheral Tissues

**DOI:** 10.1371/journal.pcbi.0020016

**Published:** 2006-03-10

**Authors:** Andrey A Ptitsyn, Sanjin Zvonic, Steven A Conrad, L. Keith Scott, Randall L Mynatt, Jeffrey M Gimble

**Affiliations:** 1 Experimental Obesity Laboratory, Louisiana State University Pennington Biomedical Research Center, Baton Rouge, Louisiana, United States of America; 2 Stem Cell Laboratory, Louisiana State University Pennington Biomedical Research Center, Baton Rouge, Louisiana, United States of America; 3 Departments of Bioinformatics and Computational Biology, Medicine, and Emergency Medicine, Louisiana State University Health Sciences Center, Shreveport, Louisiana, United States of America,; 4 Department of Veterinary Clinical Sciences, Louisiana State University School of Veterinary Medicine, Baton Rouge, Louisiana, United States of America; University of California San Diego, United States of America

## Abstract

Circadian rhythms are prevalent in most organisms. Even the smallest disturbances in the orchestration of circadian gene expression patterns among different tissues can result in functional asynchrony, at the organism level, and may to contribute to a wide range of physiologic disorders. It has been reported that as many as 5%–10% of transcribed genes in peripheral tissues follow a circadian expression pattern. We have conducted a comprehensive study of circadian gene expression on a large dataset representing three different peripheral tissues. The data have been produced in a large-scale microarray experiment covering replicate daily cycles in murine white and brown adipose tissues as well as in liver. We have applied three alternative algorithmic approaches to identify circadian oscillation in time series expression profiles. Analyses of our own data indicate that the expression of at least 7% to 21% of active genes in mouse liver, and in white and brown adipose tissues follow a daily oscillatory pattern. Indeed, analysis of data from other laboratories suggests that the percentage of genes with an oscillatory pattern may approach 50% in the liver. For the rest of the genes, oscillation appears to be obscured by stochastic noise. Our phase classification and computer simulation studies based on multiple datasets indicate no detectable boundary between oscillating and non-oscillating fractions of genes. We conclude that greater attention should be given to the potential influence of circadian mechanisms on any biological pathway related to metabolism and obesity.

## Synopsis

The metabolism of living organisms changes over the twenty-four hour daily cycle in an oscillatory manner. This repeating pattern of “peak” and “trough” expression is known as a “circadian rhythm.” We now know that the body's internal clock is controlled by a discrete group of genes. These important regulators are found in many different organs of the body, and they control expression of many other genes in the body. Using mice as an experimental animal, Ptitsyn and colleagues looked at the overall pattern of gene expression in fat tissues and the liver using three different mathematical tests. They present data indicating that the majority of active genes fluctuate rhythmically over a twenty-four hour period. This work suggests that future studies should pay close attention to the influence of the circadian rhythm in obesity and in fat metabolism.

## Introduction

The circadian, or daily, rhythm is one of the most obvious and well-studied periodic processes in living organisms. Studies of transcriptional output in different tissues report that expression of approximately 5%–15% of all mammalian genes show a circadian oscillation [1,2]. This circadian oscillation is driven by a molecular mechanism involving a transcriptional/translational feedback loop, which generates the basic rhythm driving gene expression. In mammals, the master circadian clock is located in the hypothalamus and is constantly adjusted to the daily light cycle through photic stimuli from the retina. Analysis of gene expression in peripheral tissues indicates that relatively few genes share the same circadian expression profile in more than one tissue [3].

We have completed independent circadian studies in AKR/J mice acclimated to a 12 h light: 12 h dark cycle, harvesting sets of three to five mice at 4-h intervals (details of the experiment given in Zvonic et al. [8] ). Total RNA samples from inguinal white adipose tissue (iWAT), brown adipose tissue (BAT), and liver have been assayed by RT-PCR and Affymetrix microarrays. In initial analyses, candidate genes were selected for validation by RT-PCR of their circadian expression profile in all three tissues. In further analyses, tissue samples harvested from three mice at 4-h intervals over a single 24-h period were pooled and assayed on duplicate microarrays. The resulting individual datasets for each of three different tissues contained more than 22,000 gene expression profiles. Each profile consisted of 12 time points, representing six periods of the day sampled two times. For analysis of periodicity, we considered this data as reflecting two complete daily cycles. The data was smoothed by a third-degree polynomial procedure and converted to a frequency domain (represented by a periodogram) by Discrete Fourier Transformation (DFT).

To identify periodically expressed genes, we applied three different algorithmic approaches in our analysis of the microarray data: the Fisher's *g-*test of periodogram, autocorrelation, and the permutation test. The Fisher's *g*-test estimates nonrandomness of the dominating frequency in the periodogram from the signal-to-noise ratio. In our case, the signal is a diurnal frequency, reflected by a specific peak in the periodogram, and the noise level is estimated from the height of all other frequencies represented in the periodogram (see description in Materials and Methods). Autocorrelation is based on a different principle: if a gene expression profile is formed by a periodic process, it should have parts of the profile repeating each other. Autocorrelation analysis determines if the expression profile correlates with itself to a shift of one day, thereby identifying a diurnal periodicity. These first two approaches, Fisher's *g*-test and autocorrelation, have been widely used for analysis of periodic gene expression and have been recommended in recent publications [4,5]. We have developed a permutation test as a noise-resistant alternative for the analysis of periodicity in short time series. Like the Fisher's *g*-test, it starts with DFT, producing a periodogram. However, unlike other tests, all nondiurnal frequencies are ignored along with their associated noise. The nonrandomness of only a single diurnal peak is estimated in simulation experiments by random shuffling of the original time series (see description in Materials and Methods). Although this method would be computationally ineffective for a longer time series, analysis of microarray data with only 12–24 datapoints in each expression profile offers no computational challenge. We have further employed these analytical tools to analyze two previously reported, independent circadian datasets, each prepared from murine liver [3,6]. Our findings suggest that experimental design, dataset size, and the frequency of datapoint collection can significantly impact the experimental outcome. We conclude that analyses using Fisher's *g*-test and autocorrelation alone may underestimate the contribution diurnal rhythms to global gene regulation.

## Results/Discussion

In the first step of our analysis, we applied standard procedures for the detection of circadian gene expression based on the widely accepted Fisher's *g-*test [7]. This estimation revealed only 650 genes shared in BAT, iWAT, and liver for which the *p*-value was less than 0.05, representing 12.8%, 14.8%, and 12% of the individual tissue oscillatory transcriptomes, respectively [8]. Within the periodic genes shared among these tissues were the circadian clock oscillator genes *Npas2, Bmal1 (Arntl), Per1, Per2, Per3,* and *Cry1,* as well as the circadian output gene *Dbp*. Our qRT-PCR studies [8] show these genes follow a circadian expression pattern (periodicity confirmed by cosine-fit analysis). A similar analysis of circadially expressed genes in BAT, iWAT, and liver using the permutation test developed by the authors revealed fewer (456) shared genes for which the *p*-value was less than 0.05 in all three tissues (Figure S1). However, among these genes there were more genes (nine) representing the circadian clock; based on mapping to the biological pathways (KEGG database), this is the largest functional category among genes periodically expressed in all three tissues (see Table S1). Functional annotation and mapping to the biological pathways of the tri-tissue overlapping list (see Table S2) suggests that most of the remaining genes with an oscillatory expression profile belong to the common “housekeeping” functional categories, indicative of basic cell physiology rather than tissue-specific functions.

**Figure 1:**
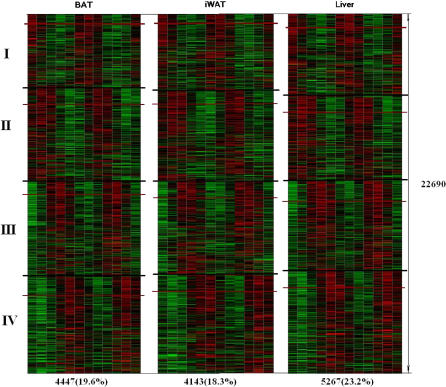
Summary of the Microarray Analysis of Circadian Periodicity and Phase in Murine BAT, iWAT, and Liver The red line marks the 0.05 cutoff for the *p*-value in Fisher's *g-*test. The Roman numerals represent the grouping of all expressed genes based on the calculated circadian phase, displayed in zeitgeber time: I, ZT0; II, ZT4; III, ZT8; IV, ZT16. To produce the heat map, all expression profiles have been *z*-scored. There seems to be no dependence between periodicity (indicated by *p*-value) and overall level of gene expression (Figure S4).

The three algorithms used for microarray data analysis (Fisher's *g*-test, autocorrelation, and permutation test) revealed that ~20% of all genes in each tissue alone followed a circadian expression pattern with confidence levels of at least 95% (*p* ≤ 0.05). The results of the analyses are summarized in Figure 1 and Table 1. Although these values were higher than previously reported in the liver [3,9,10], they may still underestimate the actual number of oscillating genes in these tissues. As in previous reports, we examined these genes one at a time, and retained only those whose individual estimated *p*-value was ≤0.05. However, from a biological perspective, it may be incorrect to consider expression values solely as independent variables as rhythmicity may exist in the noise; indeed, Hogenesch et al. [2] have speculated that the circadian clock may regulate as much as 10% of the genome, which includes genes responsible for the basic cell metabolism. Since these genes all are elements of a common, complex network, any change in the expression of an upstream element can cause a significant alteration in downstream elements of the same biological pathway. Previous studies have documented a role for the circadian clock in the regulation of key metabolic pathways [6]. This implies that large groups of genes could oscillate in a coordinated manner and their expression patterns should be studied in relation to one another.

**Table 1:**
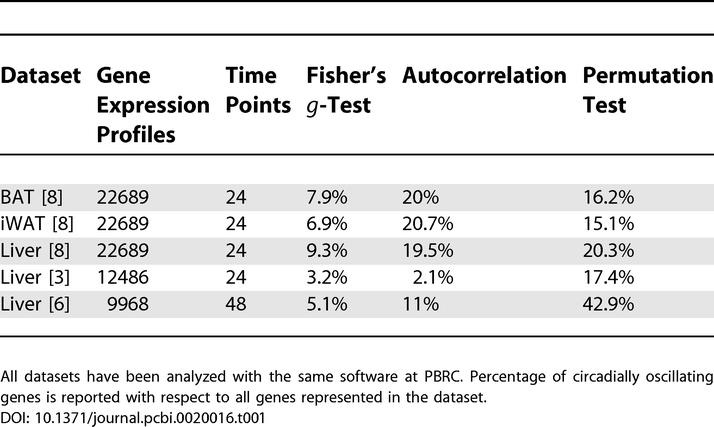
Fraction of Circadially Oscillating Genes Revealed by Different Algorithmic Approaches in Different Datasets

**Figure 2:**
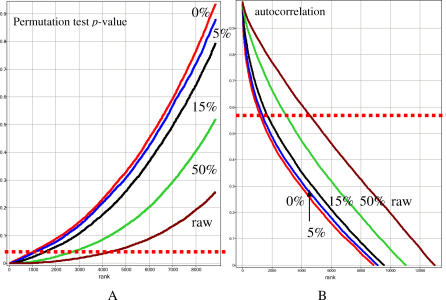
Results of the Simulation Experiment Plot (A) shows distribution of *p*-values and plot (B) shows respective autocorrelation coefficients for raw data and datasets with different proportion of genes left intact, while for the rest of the genes periodicity is eliminated by random permutation of time points. The dotted line indicates the *p* = 0.05 significance cutoff. The raw data is derived from the liver circadian expression microarray analysis.

We have conducted a computer simulation to model the distribution of *p*-values obtained using each of our three algorithms. As a test case, we modeled a system where only a limited percentage of the genes were permitted to display a circadian expression pattern. We deliberately disrupted the oscillatory pattern by randomly rearranging the time points within each series. Consecutive simulations produced datasets where all genes were reshuffled, then 0%, 5%, 15%, or 50% of randomly selected genes were left intact, while all others remained in the shuffled format. For each simulated dataset, we applied the same analysis to identify periodically expressed genes similar to the original microarray dataset. Figure 2 shows the distribution of *p*-values obtained either from the permutation test or the distribution of the highest positive autocorrelations with a circadian lag. In both cases the distributions were significantly different from the original data. Even with a complete disruption of true periodicity (0% line), ~1,000, or ~5%, of the total transcripts display an apparent false-positive circadian rhythm. Since the time series is relatively short, and there is the potential for biological and technical variability exists, it is expected that some profiles may appear “periodic” for purely stochastic reasons. As expected, the occurrence of genes identified as components of the basic circadian mechanism (such as *Clock, Bmal1, Cry,* etc.) decreases as the portion of shuffled genes increases. Indeed, none of these are found among false-positive circadian genes when all 100% are shuffled. Thus, if only ~20% of the transcriptome followed a circadian rhythm, as initially observed, the *p*-value distribution would be expected to resemble the corresponding simulated curve, rather than the profile depicted in Figure 2.

A variety of statistical models have been proposed for the description of periodic gene expression [5,11], and these include factors such as the true deviation in gene expression level and the stochastic component. Shuffling affects the order of time points, but does not alter the observed levels of gene expression. Our simulation preserves variation of gene expression and does not affect the probability that the circadian oscillations are observed due to a random arrangement of “noise.” The observed distribution can be explained if we assume that the number of periodically expressed genes is at least >50%, rather than the 10%–15% previously reported. This difference implies that relatively few genes can be identified as “circadian” based solely on a *p*-value derived from a single gene in the face of stochastic noise (signal-to-noise ratio). As the degree of stochastic noise increases due to biological variability between individual animals, technical differences introduced by the microarray methodology, and other sources of “background,” the accuracy of time series analyses of circadian expression profiles decreases.

The observed distribution of *p*-values is clearly different from the simulated data, where an identified fraction of nonperiodic genes have been introduced. This conclusion is substantiated by the gene expression heatmap, presented in Figure 1. Within each phase-group (based on the relative circadian time of peak expression), expression profiles are sorted by their *p*-values in ascending order, and illustrate that there is no obvious point at which we can differentiate between periodic and constitutively expressed genes, thus recapitulating the fact that only a small fraction of all genes examined do not follow an oscillatory expression pattern. Alternatively, there may be enough circadian genes mixed in to trick the eye. Nevertheless, we postulate that the fraction of expressed genes with a constant steady state mRNA level (unaffected by circadian oscillation) does not exceed 50% of all genes (Table 1). The introduction of alternative analytical tools to more detailed circadian datasets is likely to reveal additional genes as circadially regulated. The concept of “steady state mRNA levels” may become a temporally dependent term.

To further prove the conclusion that the majority of the expressed genes oscillate with a circadian rhythm we have reanalyzed two additional independent datasets. The first independent dataset, provided courtesy of Dr. Storch [3], was collected from murine liver and heart. Each dataset consisted of 12 time points collected at even intervals of 4 h over a period of 48 h. The experimental design was similar to our own based on number of time points and the period of observation. The summary of analysis for the liver dataset is presented in Table 1 and Figure S2. In our analysis of their data, both the Fisher's *g*-test and the autocorrelation approach have yielded fewer circadian genes (3.2% and 2.2%, respectively) than their reports. However, the permutation test revealed circadian pattern in 17.4% of all genes, which is more than originally reported by the authors (4.6% of all genes in the liver dataset). As in the previous analyses, a visual inspection of the heatmap of phase-grouped expression profiles showed no clear landmark demarcating that fraction of genes with a non-oscillating profile, i.e., a flat line. Indeed, it would appear that the expression profile of the majority of genes shows an oscillatory pattern.

The second dataset was provided courtesy of Dr. Hogenesch [6] and contains microarray expression profiles of nearly 10,000 genes in murine liver, measured at 4-h intervals over a 48-h period with two replicates for each time point. Although similar to the microarray used in our analysis of the murine liver, this dataset represents fewer genes, has twice as many datapoints, and differs in the methods of sample collection and processing. Nevertheless, use of the same computational analyses of periodicities led to similar results. The Fisher's *g-*test revealed 511 circadian expressed genes with *p* ≤ 0.05, or ~5% of all genes examined (Table 1). The autocorrelation method identified 1278 or ~13% of all genes as circadially expressed. In contrast to the first two methods, our permutation test reported an oscillatory profile in ~43% of all genes. The heatmap, generated after assigning a phase to each expression profile, suggested that this latter method is likely to be a correct, although still conservative, estimation of the true number of circadially expressed genes (Figure S3). We conclude that the increased number and frequency of datapoints collected in this dataset permits a more accurate assignment of circadian rhythmicity to individual genes using the permutation test.

**Figure 3:**
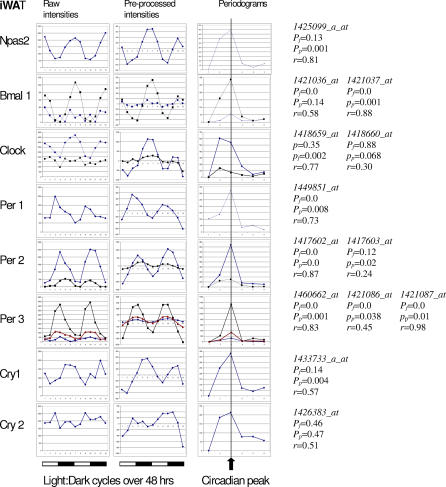
Expression Profiles and Periodicity Analysis of Basic Circadian Rhythm Genes in iWAT The first column plots are raw expression values as reported by the Affymetrix MAS5 algorithm. The second column plots show the same data after preprocessing (central value adjustment, polynomial smoothing, and trimmed mean subtraction). The third column presents the periodograms resulting from DFT analysis. Highlighted is a single peak corresponding to the circadian rhythm (two complete cycles in 48 h). For each probeset, representing a particular gene, the results of Fisher's *g-*test *(p_f_),* permutation test *(p_p_),* and autocorrelation *(r)* with circadian lag are listed to the right.

**Figure 4:**
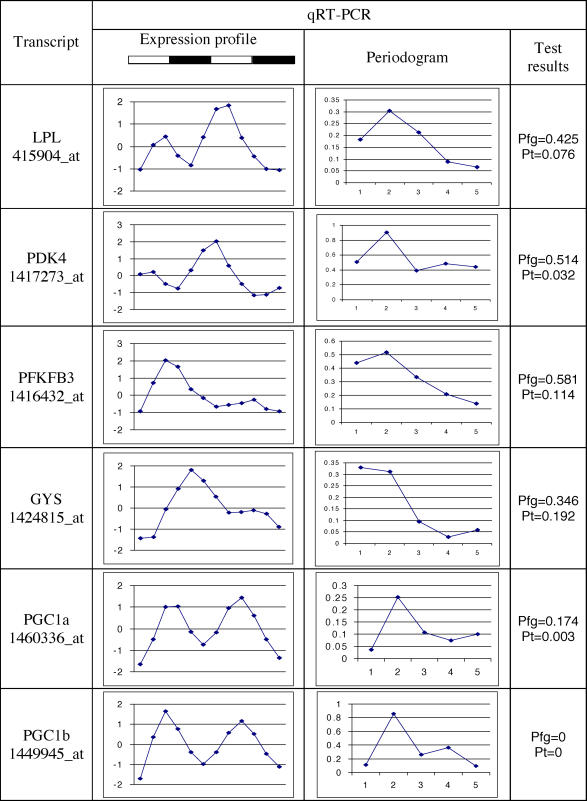
Experimental qRT-PCR Verification of Selected Microarray Expression Profiles in Liver. The qRT-PCR expression profiles for selected transcripts represented on microarray (probe names given in brackets).

**Figure 5:**
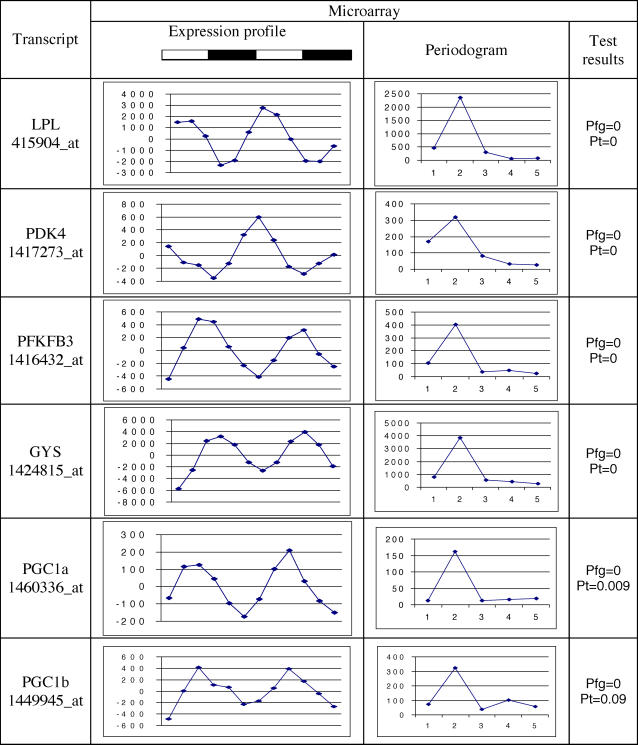
Experimental Verification of Selected Microarray Expression Profiles in Liver (continued from Figure 4). The alternative qRT-PCR expression profiles presented on Figure 4 are consistent with microarray data in phase and visual presence of diurnal oscillation pattern.

Recent reports have connected the basic circadian mechanisms to nutrient homeostasis [12]. Specifically, at least two components of the basic circadian pacemaker, *Bmal1* and *Clock,* were found to regulate glucose levels, and thus play a significant role in the energy balance [12]. This finding is consistent with the accepted view that circadian clocks are important in driving the activity and feeding behavior in mammals. Our analysis of a large collection of time series expression profiles in peripheral tissues leads to the conclusion that a high percentage, possibly a majority, of all genes has an oscillatory expression pattern. Direct application of the standard methods for identification of circadian expressed genes to our data already revealed a larger percentage of oscillating genes relative to previous reports, ranging from 7%–21% of all, in contrast to previously reported fraction of 5%–15 % of “actively expressed” genes. To allow for a direct comparison, we have calculated the percentage of expressed genes based on the total number of gene expression profiles available in each particular dataset. The results are presented in Table 1. In part, the increase in the numbers of oscillating genes could be explained by the improvement in the microarray technology over recent years, permitting more transcripts to be identified. Yet, our simulation studies have shown that, if there were fewer oscillating genes, we would continue to detect them, even with the present degree of stochastic noncircadian variation. We believe the discrepancy may result from the traditional 0.05 cutoff for *p*-values as well as the assumption that each gene's expression profile should be tested independently. The data presented in Figure 3 illustrate the latter point. In cases such as *Per2,* where the microarray contained multiple probe sets for an individual gene, the *p*-values were not identical, and fell both above and below the 0.05 cutoff points. This observation may also account for the low percentage of overlap between the lists of circadian-expressed genes identified in different tissues [3,8]. The lists of genes in all three tissues coexpressed in a circadian manner with *p* ≤ 0.05 were dominated by those involved in basic cell metabolic activities as well as the elements of the circadian molecular oscillator themselves [3,8]. Additional qRT-PCR analysis was performed using primers for a randomly selected subset of genes included on the Affymetrix microarray that were not directly associated with the core circadian oscillator (Figures 4, 5). The specific genes were: glycogen synthase 2 (GYS), lipoprotein lipase (LPL), peroxisome proliferator activated receptor γ coactivators 1 α and β (PGC1a, PGC1b), 6-phosphofructo-2 kinase/fructose 2,6 biphosphatase 3 (PFKFB3), and pyruvate dehydrogenase kinase isozyme 4 (PDK4). These genes had been identified as oscillating based on the permutation analysis uniquely in our liver dataset but not by the Fisher's *g* test in the liver datasets previously published [3,6]. All genes in the subset play an important role in energy metabolism and showed a pattern of peaks consistent with two complete diurnal periods. All qRT-PCR expression profiles displayed the same phase of oscillation as their microarray-derived counterparts. It is of interest to note that previously published studies have reported a diurnal or circadian oscillation of lipoprotein lipase serum enzyme levels in mice, rats, and humans, consistent with the current observations [13–16].

Permutation analysis of the liver data collected with the most comprehensive experimental design (based on the number of independent datapoints) [6], identified circadian expression in ~43% of the total number of genes (Table 1). The application of global transcriptomic approaches and statistical analytical tools permits a greater appreciation of the potential contribution of circadian biology to metabolism in peripheral tissues [3,4,6,9,10]. Our analyses lead us to conclude that circadian rhythms influence the expression of the majority of genes in metabolic tissues. Previous analyses using alternative statistical tools may have underestimated the full extent of the circadian contribution to gene regulation. The main reason for this underestimation might be that the null hypothesis is formulated on an equivocal assumption. It is commonly assumed that the default state for a gene expression over time is constant, only obscured by stochastic noise. Most currently applied methods test the data for the presence of oscillation based on signal-to-noise ratio. Applied on a gene-by-gene basis, these methods have limited resolution ability, curbed by the number of replicated periods in time series. Applying different methods, we observed continuous improvement but no obvious limit to the number of genes expressed in circadian oscillation pattern. This observation makes us question the basic assumption of the default non-oscillating expression. The alternative assumption would be that all genes in a living tissue are expressed in an oscillating pattern, only obscured by stochastic deviations. For a relatively small number of genes, a constant level of expression may be imposed by their function. In this case, expression profiles should be statistically tested for the absence of a circadian pattern. Our thesis of default assumption of oscillation for the timeline expression profiles is corroborated by the analysis of phase distribution in different datasets, presented in Figure 1 and in Figures S2 and S3. Each gene is assigned a proper phase by maximum correlation to an ideal sinusoidal profile.

Our conclusions have significant implications for investigators examining metabolic pathways related to diabetes, obesity, and their associated co-morbidities. First of all, the assumption of oscillation means that synchronization of time at which gene expression is measured for different experimental conditions is important for the majority of genes, not only for a fraction of circadian genes. Phase, along with fold change or amplitude, becomes an important factor in understanding gene expression. The biological importance of oscillation cannot be estimated by simply measuring amplitude and should be considered within a specific functional, spatial, and temporal context. Relatively small diurnal variations of upstream regulators may have a large impact on the downstream functions. Also, even without an absolute change in peak expression level, a phase shift can be equivalent to a downregulation at a defined time point. Hence, gene interaction within biological pathways should also be perceived and modeled in the context of the phase of a dynamic diurnal oscillation, similar to an alternating electrical current circuit, rather than by assuming it to be a static or unchanging process, similar to a direct electrical current circuit.

## Materials and Methods

### Affymetrix oligonucleotide microarray gene expression analysis.

The microarrays were performed as described in Zvonic et al. [8]. The study examined BAT, iWAT, and liver harvested every 4 h over a 24-h period from AKR/J male mice maintained under a constant 12-h light–12-h dark cycle in accordance with previous circadian studies of central and peripheral tissue gene expression profiles [17–20]. RNA integrity was assessed by electrophoresis on the Agilent 2100 Bioanalyzer (Agilent Technologies, Palo Alto, California, United States). Double-stranded cDNA was synthesized from approximately 9 μg total RNA using a Superscript cDNA Synthesis Kit (Invitrogen, Carlsbad, California, United States) in combination with a T7-(dT)24 primer. Biotinylated cRNA was transcribed in vitro using the GeneChip IVT Labeling Kit (Affymetrix, Santa Clara, California, United States) and purified using the GeneChip Sample Cleanup Module. Ten micrograms of purified cRNA was fragmented by incubation in fragmentation buffer (200 mM Tris-acetate [pH 8.1], 500 mM potassium acetate, 150 mM magnesium acetate) at 94 °C for 35 min and chilled on ice. Six and a half micrograms of fragmented biotin-labeled cRNA was hybridized to the Mouse Genome 430A 2.0 Array (Affymetrix). Microarrays were performed in duplicate at each time points as suggested by [21]. Arrays were incubated for 16 h at 45 °C with constant rotation (60 rpm), washed, and then stained for 10 min at 25 °C with 10 μg /mL streptavidin-R phycoerythrin (Vector Laboratories, Burlingame, California, United States) followed by 3 μg /mL biotinylated goat anti-streptavidin antibody (Vector Laboratories) for 10 min at 25 °C. Arrays were then stained once again with streptavidin-R phycoerythrin for 10 min at 25 °C. After washing and staining, the arrays were scanned using a GeneChip Scanner 3000. Pixel intensities were measured, expression signals were analyzed, and features extracted using the commercial software package GeneChip Operating Software version 1.2 (Affymetrix). All 22,690 expression profiles resulting from the standard Affymetrix processing were used in the consequent analysis of periodicity without further filtering. In addition to the standard Affymetrix processing, we have performed a median adjustment to compensate for possible systematic variation of intensity between chips. In each time series, the data was smoothed by the third-degree polynomial procedure, and the median of each profile was subtracted from each point to center all deviations at about zero. To produce the heatmaps presented on Figure 1 and Figures S1–S3, we have also equalized the amplitude of variation by *z*-score transformation. The plot was produced using Spotfire Decisionsite software (Spotfire, Somerville, Massachusetts, United States).

### Spectral analysis.

Consider a series of microarray expression values for gene *x* with *N* samples of the form


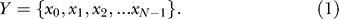


This series can be converted from time-domain, where each variable represents a measurement in time to a frequency domain using the DFT algorithm. Frequency domain representation of the series of experiments is also known as periodogram, which can be denoted by *I*(*ω*):


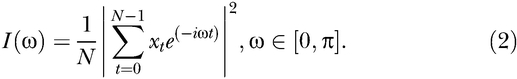


If a time series has a significant sinusoidal component with frequency *ω*∈[0, *π*], then the periodogram exhibits a peak at that frequency with a high probability. Conversely, if the time series is a purely random process (also known as “white noise”), then the plot of the periodogram against the Fourier frequencies approaches a straight line [22].

### Fisher's *g*-test.

Significance of the observed periodicity can be estimated by Fisher *g-*statistics, as recently recommended in [5]. Fisher derived an exact test of the maximum periodogram coordinate by introducing the *g*-statistic


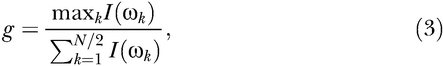


Where *I*(*ω_k_*) is a *k-*th peak of the periodogram. Large values of *g* indicate a nonrandom periodicity. To calculate the *p*-value of the test under the null hypothesis we use the exact distribution of *g* given by


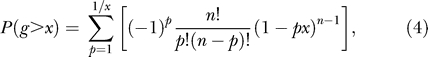


where *n* = [*N/*2] and *p* is the largest integer less than 1*/x*.

To account for multiple testing problems, we employ the method of FDR as a multiple comparison procedure [23]. This method is less conservative compared to the classic Bonferroni correction, which make it more applicable for testing large numbers of relatively short time series produced by microarray experiments. The FDR threshold is determined from the observed *p*-value distribution, and hence is adaptive to the actual data [5].

Consider the set of ordered *p*-values *p(1), p(2), . . .* , *p(G)* with corresponding genes *g(1), g(2), . . .* , *g(G),* and apply the following algorithm:


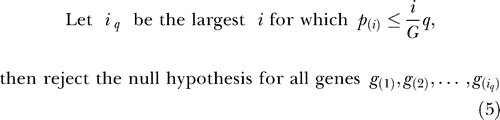


It has been shown that this procedure controls the FDR at level *q* [23]. This algorithm closely follows the guidelines recommended for analysis of periodicities in time-series microarray data [5] with exception that we applied locally developed C++ code instead of R scripts.

### Permutation test.

The alternative test for significance of a particular (in our case circadian) periodicity among large numbers of gene expression profiles is based on the random permutation procedure. Consider a time series *Y* = {x_0_,x_1_,x_2_, … x_*N*−1_}, in which technical variation approaches or even exceeds the amplitude of periodic expression. In a very short time series there is a significant probability to observe a periodicity due to stochastic reasons. However, the periodic change of the base expression level can still be identified in spite of the high noise level. Let *YR* be a random permutation of the time series *Y* and its corresponding periodogram *IR*(*ω*). If the periodogram *IY*(*ω*) contains a significant peak corresponding to a particular frequency (for example, circadian) this peak results from a particular order of observations in the series *Y*. A random permutation would preserve the same noise level, but not the periodicity. After DFT, a periodogram *IR*(*ω*) represents only the peaks occurring by chance. To avoid random reinstitution of periodicity of length *T* (in this case circadian), we generate *YR* by multiple shuffling of randomly selected time points 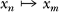, where 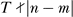, i.e., each shuffle swaps time points from different phases. Comparing permutations with deliberately wiped out periodicity to the original time series we can estimate whether a particular order of observations (i.e., time series) is important. For each gene expression profile we generate two series of min(*n*!,1000) random permutations. Each permutated series *YR* is transformed to the frequency domain and a single peak of the periodogram *IR*(*ω*) is stored. The *p*-value for the null hypothesis of random nature of a particular peak of periodogram can be estimated by comparing the stored *IR*(*ω*) values to the observed *I*(*ω*):


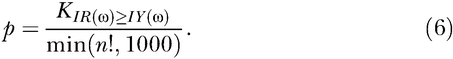


Here *K* is the number of permutated series *YR* for which the circadian peak of periodogram is higher or equal to that of the original time series *Y*. High *p*-value exceeding the threshold, for example 0.05, means that at least 5 out of 100 random permutations of time series produce a periodogram with the same or higher peak, corresponding to a given periodicity. Low *p*-values of indicate a significant difference between periodograms *IR*(*ω*) preserving circadian periodicity and purely random periodograms with the same level of technical variation.

### Autocorrelation.

For a given a discrete time series *Y* = {x_0_,x_1_,x_2_, … x_*N*−1_} the autocorrelation is simply the correlation of the expression profile against itself with a frame shift of *k* datapoints (where 0 ≤ *k* ≤ *N* − 1, often referred to as the lag).

For the time shift *f,* defined as *f =i + k* if *i + k < N* and *f =i + k* − N otherwise:


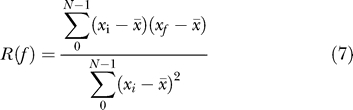


For each time series we calculate the maximum positive *R*(*f*) among all possible phase shifts *f* and use 0.05 significance cutoff values for correlation coefficient. Time series that shows significant autocorrelation *R*(*f*) with the lag *f* corresponding to one day (six datapoints) are considered circadially expressed.

### Phase classification.

We have assigned phase to each expression time series by computing cross-correlation


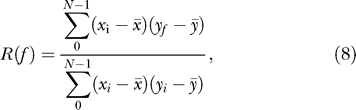


where *x* is a gene expression time series of *N* points and *y* is an artificially generated profile of ideal cosine function


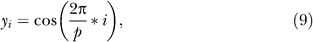


where *p* is the number of time points in a complete circadian cycle; for example *p* = 6 time points in the Zvonic et al. [8] dataset. To account for all phases, the artificial cosine curve profile has been regenerated with a phase shift by one time point. The highest correlation among all possible phase shifts was assigned as the most probable phase. The significance of periodicity was not assessed at this point, it was done separately by three independent procedures described above. All expression profiles were sorted (classified) first by the assigned phase then by ascending *p*-value estimated by one of the described algorithms. The heatmap was generated from the table of sorted time series expression profiles using Spotfire Decisionsite software (Spotfire).

## Supporting Information

Figure S1 Venn Diagram of Circadially Expressed Genes Revealed by Permutation Test with *p* = 0.05 Cutoff (16 KB PDF)

Figure S2 Results of Phase Classification for Harvard Murine Liver Time Series Data Phase is assigned to each expression profile based on the maximal correlation to an artificial cosinusoid profile with a given phase shift. Phase I starts with a peak value at time zero, thus there is a peak in the middle and a rise at the end. For other phases there are two red zones, corresponding to the peak expression values, spaced by dark or green areas. This pattern extends far beyond 575 out of 12,486 genes reported in [3]. As in all other discussed datasets, the period of observation covers two complete daily cycles.
(111 KB PDF)

Figure S3 Results of Phase Classification for GNF Murine Liver Time Series Data Phase is assigned to each expression profile based on the maximal correlation to an artificial cosinusoid profile with a given phase shift. Phase I starts with a peak value at time zero, thus there is a peak in the middle and a rise at the end. For other phases there are two red zones, corresponding to the peak expression values, spaced by dark or green areas. This pattern is prominent across the absolute majority of expressed genes, not merely 10%–15% of each phase category.
(232 KB PDF)

Figure S4 Relation between *p*-Value (Estimated by Permutation Test) In all three tissues, the mean expression level (raw) is plotted on the abscissa (*x*-axis) and the corresponding *p*-value on the ordinate (*y*-axis).
(2.2 MB PDF)

Protocol S1 Experimental Procedures (51 KB PDF)

Table S1 KEGG Charts The relative abundance of KEGG biological pathways represented in the subset of transcripts for which oscillation is detected in all three tissues (BAT, iWAT, and liver). Mapping to the KEGG database was performed using the DAVID online service (http://david.niaid.nih.gov/david).
(43 KB PDF)

Table S2 Functional Annotation of Transcripts for Which Circadian Oscillation Is Detected in All Three Tissues (BAT, iWAT, and Liver) (242 KB PDF)

## Abbreviations

BAT: brown adipose tissue, DFT, Discrete Fourier Transformation
iWAT: inguinal white adipose tissue
