# Supplementary material for: Circadian Clocks Are Resounding in Peripheral Tissues
Source: PLoS Comput Biol. 2006 Mar 10;2(3):e16. doi: 10.1371/journal.pcbi.0020016 (PMC1391918; doi:10.1371/journal.pcbi.0020016)
Supplement: Supplementary file 1 [file pcbi.0020016.sd001.pdf]

Experimental Procedures

In Vivo Circadian Studies

Studies were conducted using 8-10 week old male AKR/J mice obtained from the Jackson Laboratories (Bar Harbor, ME). The animals were acclimated to a regular chow diet (Purina 5015) *ad libitum*, under a strict 12-hour light:12-hour dark cycle for 2 weeks. During this period, all animals were handled frequently by the staff to reduce the stress introduced by human contact. Following the acclimation period, animals were sacrificed in groups of 3 (2003 study) or 5 (2004 study) animals every 4 hours over a 48-hour period. Animals in the restricted feeding (RF) study were divided into two groups; "control" animals had *ad libitum* access to food, while the "RF" animals had access to food only during the 12-hour light period. Body weight, as well as food intake, were monitored daily for each animal during the 7-day restricted feeding period. These animals were sacrificed in groups of 3 every 4 hours over a 24-hour period. All animals were sacrificed by cervical dislocation or CO_2 asphyxiation. From the individual animals, we harvested serum, inguinal white adipose tissue (iWAT), epididymal WAT (eWAT), brown adipose tissue (BAT), and liver.

Quantitative Real Time PCR (qRT-PCR)

Total RNA was purified from tissues collected using TriReagent (Molecular Research Center) according to the manufacturer's specifications. Approximately 2µg of total RNA was reverse transcribed using Moloney murine leukemia virus reverse transcriptase (MMLV-RT; Promega) with Oligo dT at 42ºC for 1 hour in a 20µL reaction. Primers for genes of interest were identified using the Primer Express software (Applied Biosystems). Within each PCR experiment, the dynamic range of amplification was first determined using pooled cDNA from each tissue set. PCR was performed on diluted cDNA samples with SYBR® Green PCR Master Mix (Applied Biosystems) using the 7900 Real Time PCR system (Applied Biosystems) under universal cycling conditions (95°C for 10 min; 40 cycles of 95°C for 15 sec; then 60°C for 1 min). All results were normalized relative to a *Cyclophilin B* expression control.

Serum Analysis

Commercially available ELISA kits for melatonin (Research Diagnostics, Flanders, NJ, Cat. # RE54021), leptin (Linco Research, Inc., St Louis, MO, Cat. # EZML-82K), and RIA kit for corticosterone (Mp Biomedicals, LLC, Orangeburg, NY, Cat. # 07-120102) were used according to the manufacturer's protocols. Replicate assays were performed on serum samples pooled from n = 3-5 animals harvested at individual time points.

Affymetrix Oligonucleotide Microarray Gene Expression Analysis

RNA integrity was assessed by electrophoresis on the Agilent 2100 Bioanalyzer (Agilent Technologies, Palo Alto, CA). Double-stranded cDNA was synthesized from approximately 9 µg total RNA using a Superscript cDNA Synthesis Kit (Invitrogen, Carlsbad, CA) in combination with a T7-$(dT)_{24}$ primer. Biotinylated cRNA was transcribed *in vitro* using the GeneChip IVT Labeling Kit (Affymetrix, Santa Clara, CA) and purified using the GeneChip Sample Cleanup Module. Ten micrograms of purified cRNA was fragmented by incubation in fragmentation buffer (200 mM Tris-acetate, pH

8.1, 500 mM potassium acetate, 150 mM magnesium acetate) at 94°C for 35 minutes and chilled on ice. Six and a half micrograms of fragmented biotin-labeled cRNA was hybridized to the Mouse Genome 430A 2.0 Array (Affymetrix), interrogating over 14,000 substantiated mouse genes. Arrays were incubated for 16 hr at 45°C with constant rotation (60 rpm), washed, and then stained for 10 min at 25°C with 10 µg /mL streptavidin-R phycoerythrin (Vector Laboratories, Burlingame, CA) followed by 3 µg /mL biotinylated goat anti-streptavidin antibody (Vector Laboratories) for 10 min at 25°C. Arrays were then stained once again with streptavidin-R phycoerythrin for 10 min at 25°C. After washing and staining, the arrays were scanned using a GeneChip Scanner 3000. Pixel intensities were measured, expression signals were analyzed and features extracted using the commercial software package GeneChip Operating Software v.1.2 (Affymetrix). Data mining and statistical analyses were performed with Data Mining Tool v.3.0 (Affymetrix) algorithms.

Affymetrix Microarray Data Normalization and Statistical Analysis
Arrays were globally scaled to a target intensity value of 2500 in order to compare individual experiments. The absolute call (present, marginal, absent) of each gene expression in each sample, as well as the direction of change, and fold change of gene expressions between samples were identified using the above-mentioned software.
