# Supplementary material for: Circadian Clocks Are Resounding in Peripheral Tissues
Source: PLoS Comput Biol. 2006 Mar 10;2(3):e16. doi: 10.1371/journal.pcbi.0020016 (PMC1391918; doi:10.1371/journal.pcbi.0020016)
Supplement: Supplementary file 2 [file pcbi.0020016.sg001.pdf]

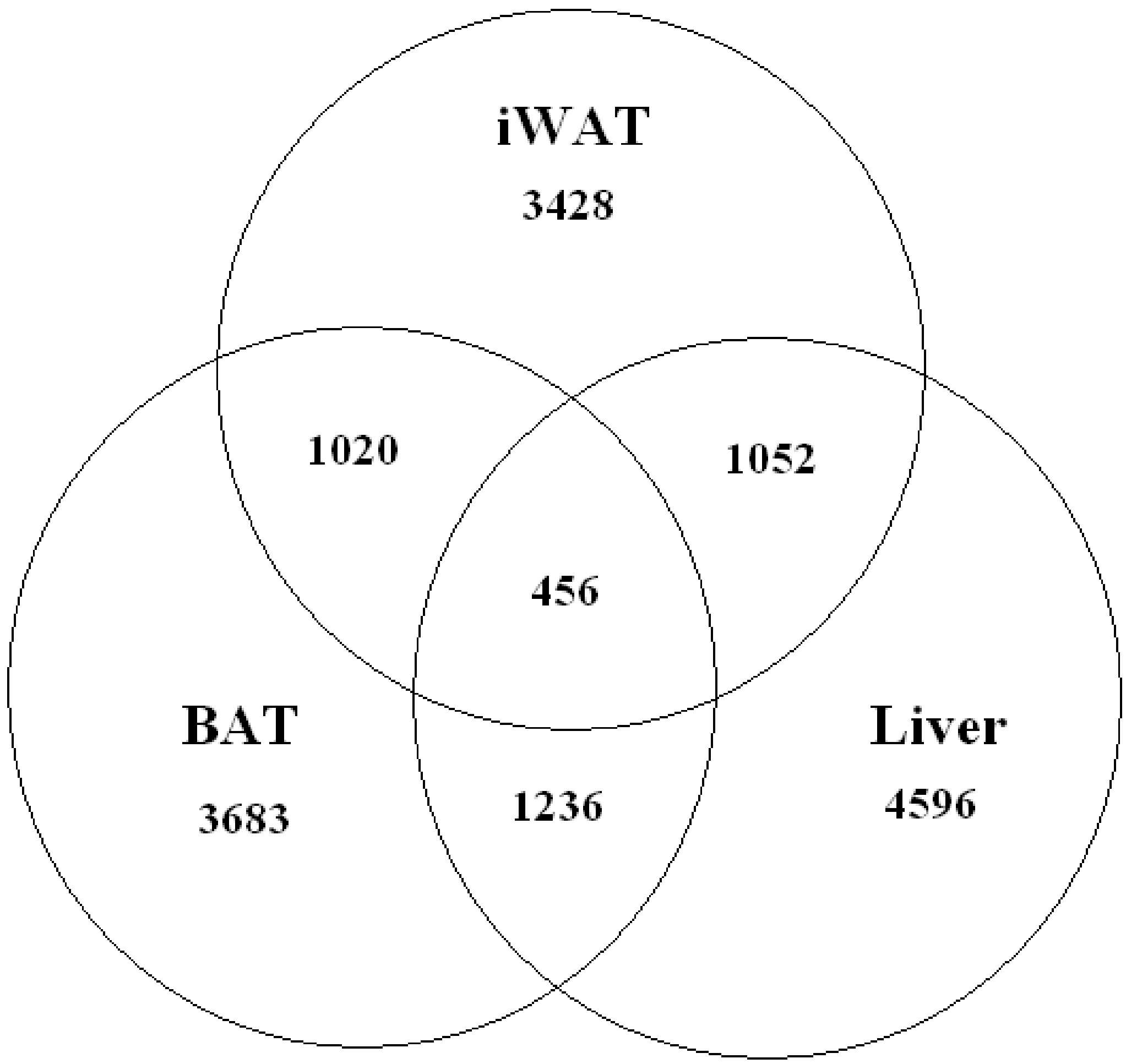

Supplemental Figure 1. Venn diagram of circadially expressed genes revealed by permutation test with *p*=0.05 cutoff.
