# Supplementary material for: Circadian Clocks Are Resounding in Peripheral Tissues
Source: PLoS Comput Biol. 2006 Mar 10;2(3):e16. doi: 10.1371/journal.pcbi.0020016 (PMC1391918; doi:10.1371/journal.pcbi.0020016)
Supplement: Supplementary file 3 [file pcbi.0020016.sg002.pdf]

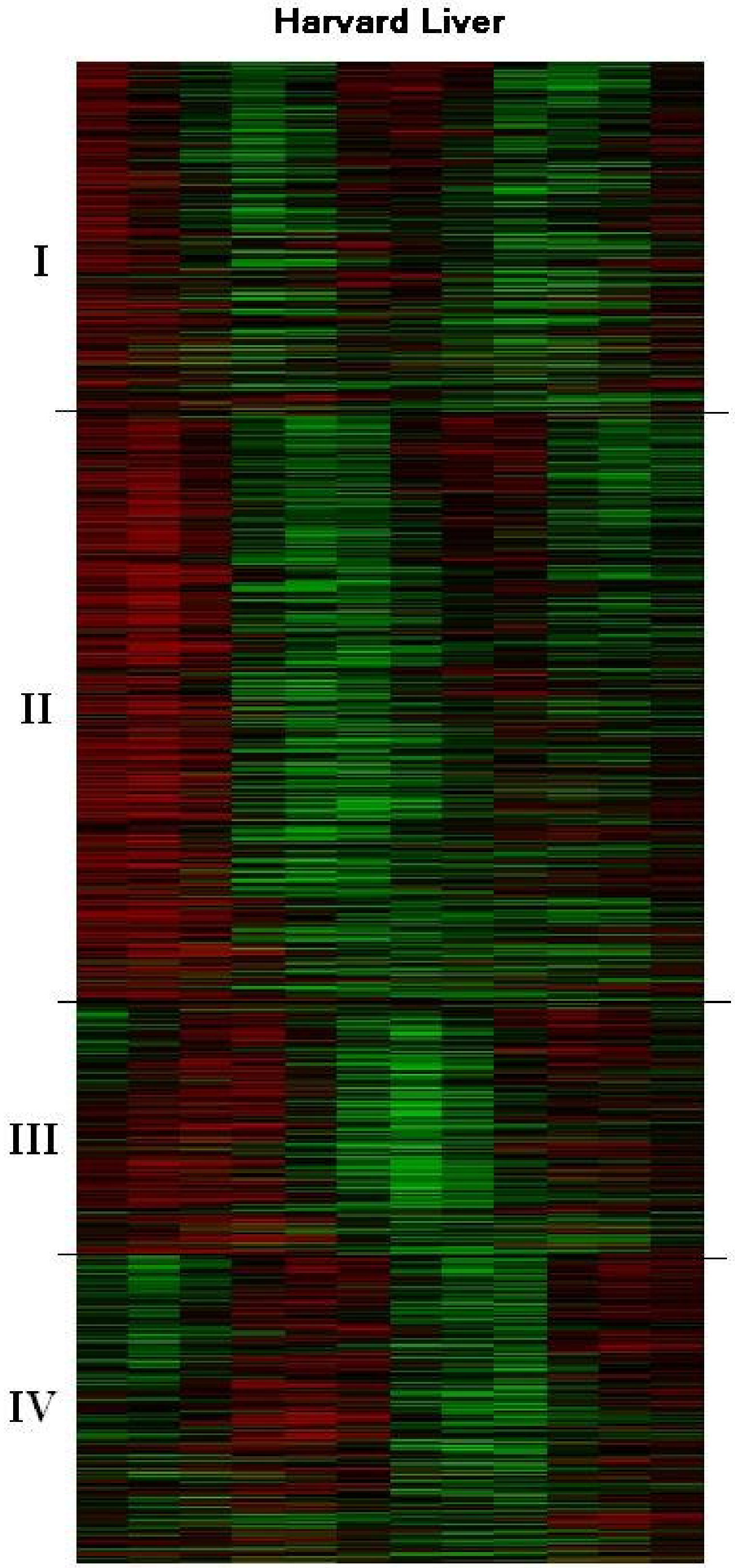

Supplemental Figure 2. Results of phase classification for Harvard murine liver time series data (Storch et al. 2002). Phase is assigned to each expression profile based on the maximal correlation to an artificial cosinusoid profile with a given phase shift. Phase I starts with a peak value at time 0, thus there is a peak in the middle and a rise at the end. For other phases there are two red zones, corresponding to the peak expression values, spaced by dark or green areas. This pattern is extends far beyond 575 out of 12486 genes, reported by the original authors. Like in all other discussed data sets, the period of observation covers 2 complete daily cycles.
