# Supplementary material for: Circadian Clocks Are Resounding in Peripheral Tissues
Source: PLoS Comput Biol. 2006 Mar 10;2(3):e16. doi: 10.1371/journal.pcbi.0020016 (PMC1391918; doi:10.1371/journal.pcbi.0020016)
Supplement: Supplementary file 4 [file pcbi.0020016.sg003.pdf]

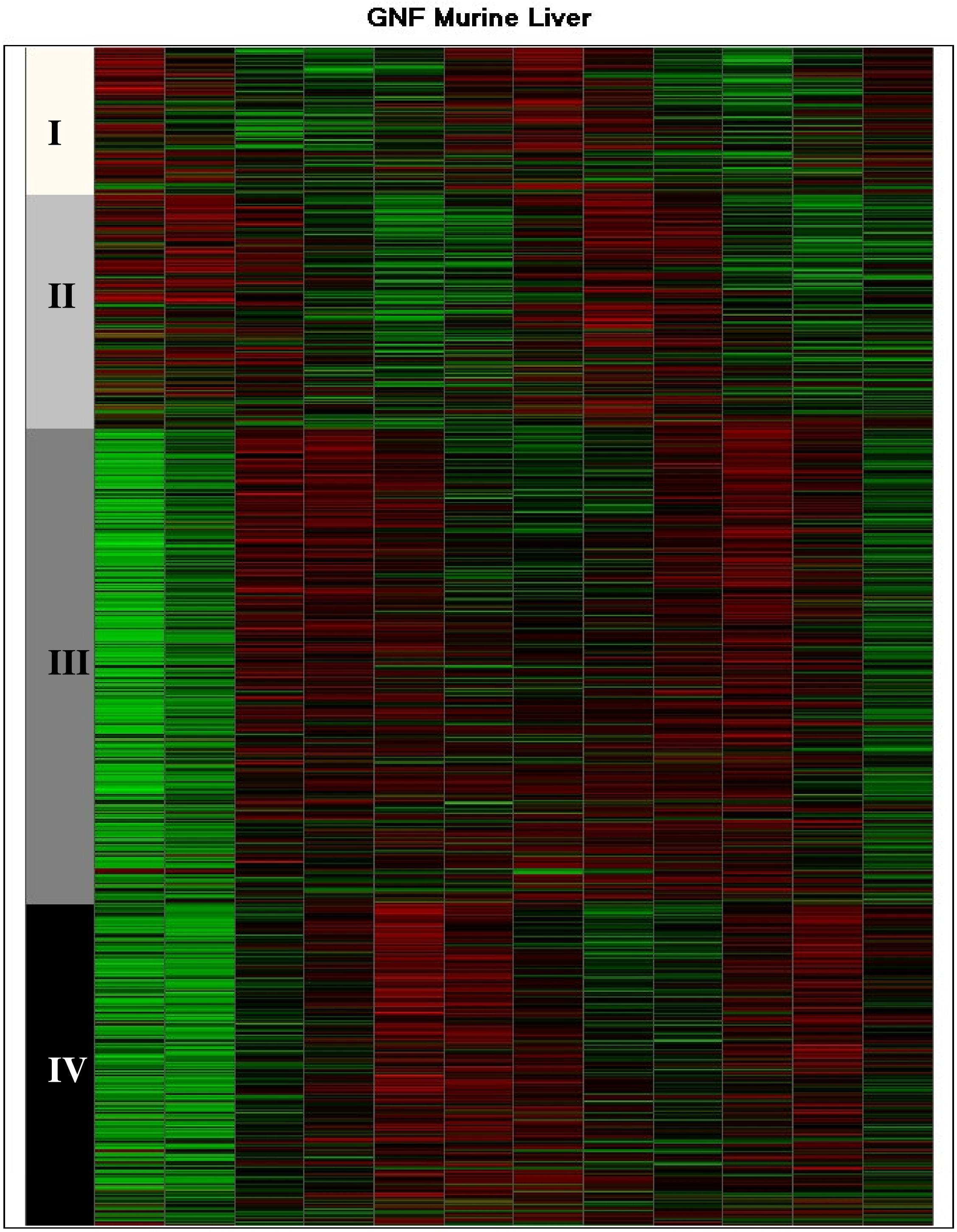

Supplemental Figure 3. Results of phase classification for GNF murine liver time series data. Phase is assigned to each expression profile based on the maximal correlation to an artificial cosinusoid profile with a given phase shift. Phase I starts with a peak value at time 0, thus there is a peak in the middle and a rise at the end. For other phases there are two red zones, corresponding to the peak expression values, spaced by dark or green areas. This pattern is prominent across the absolute majority of expressed genes, not merely 10-15% of each phase category.
