# Supplementary material for: Circadian Clocks Are Resounding in Peripheral Tissues
Source: PLoS Comput Biol. 2006 Mar 10;2(3):e16. doi: 10.1371/journal.pcbi.0020016 (PMC1391918; doi:10.1371/journal.pcbi.0020016)
Supplement: Supplementary file 5 [file pcbi.0020016.sg004.pdf]

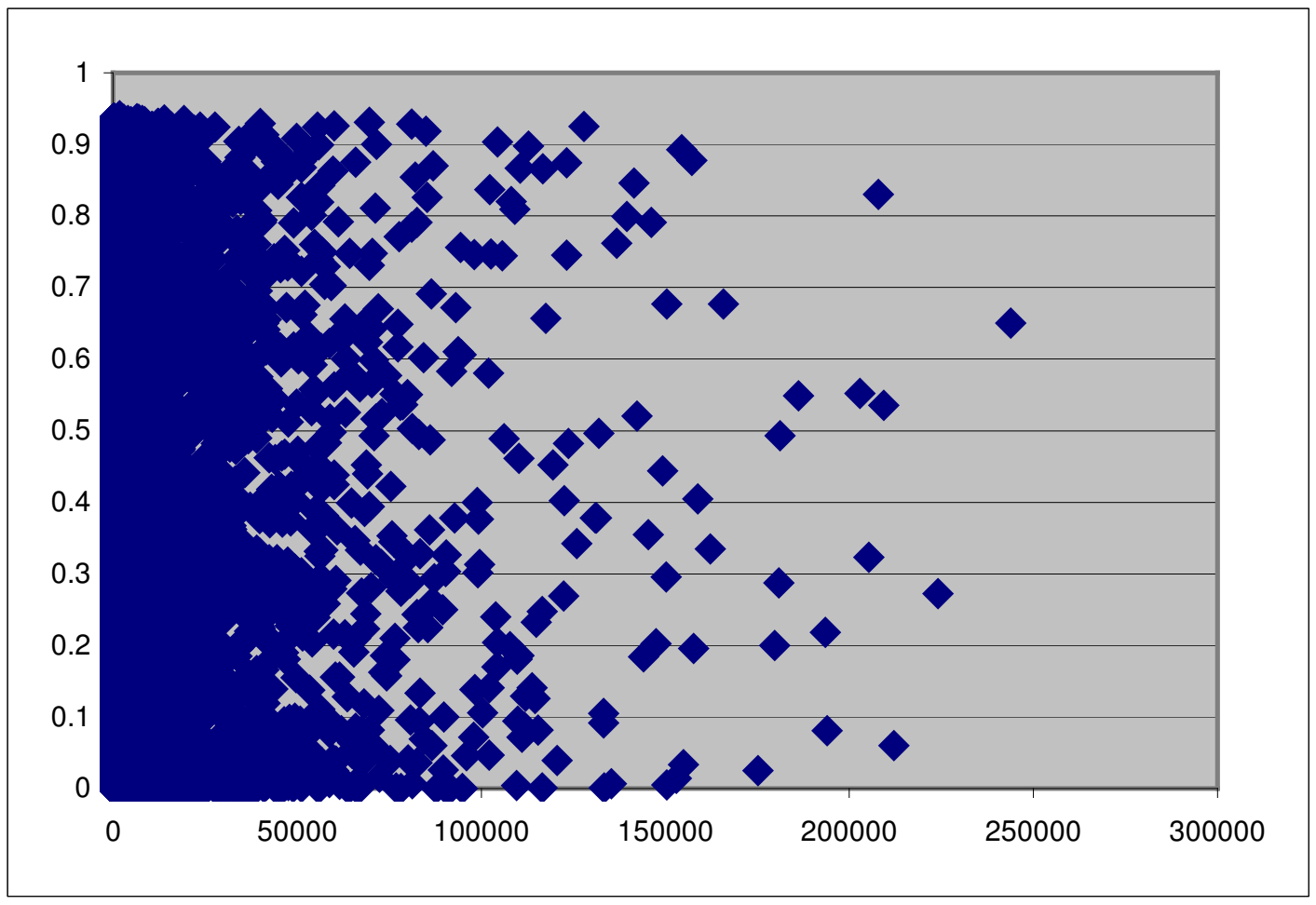

Liver

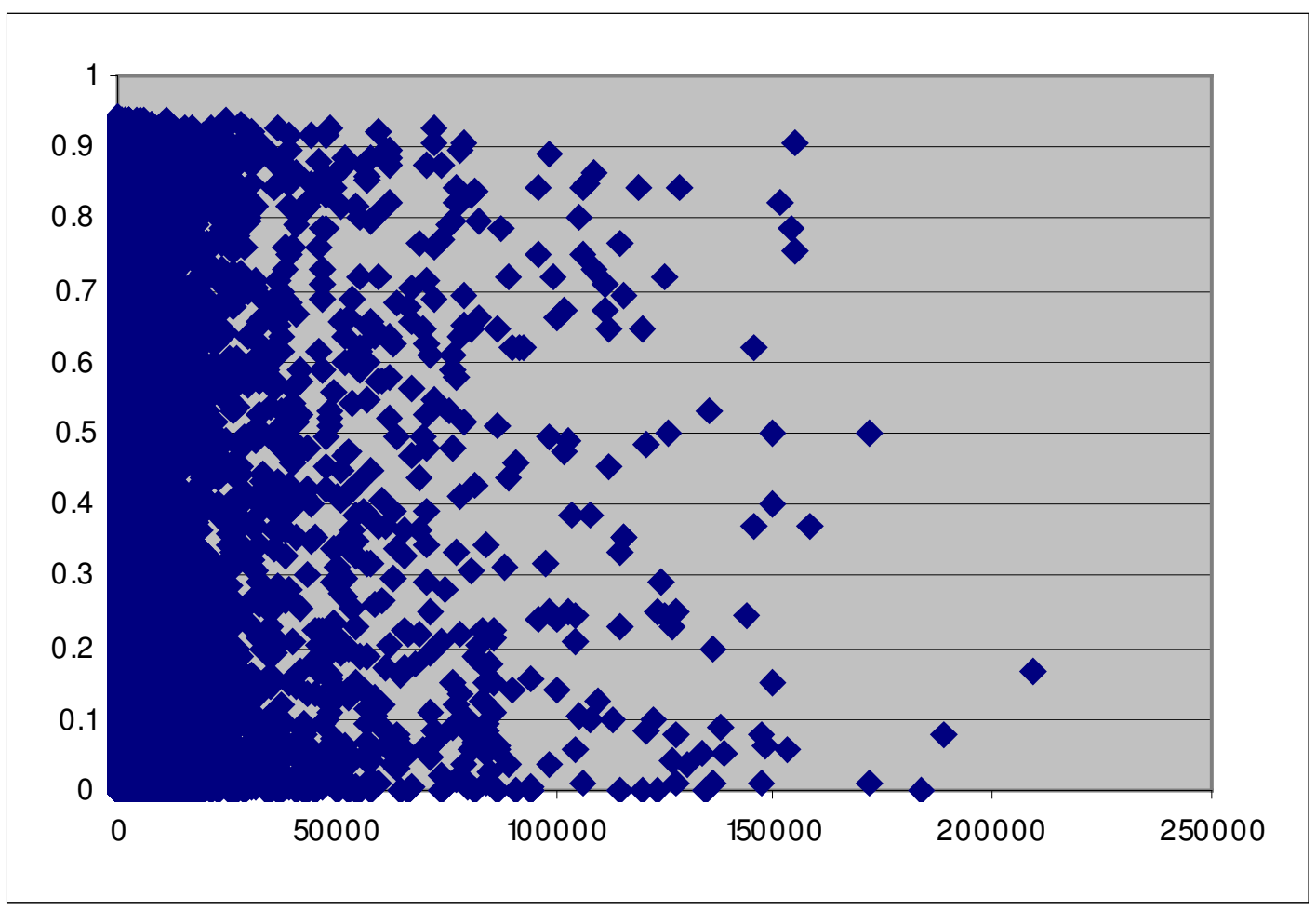

BAT

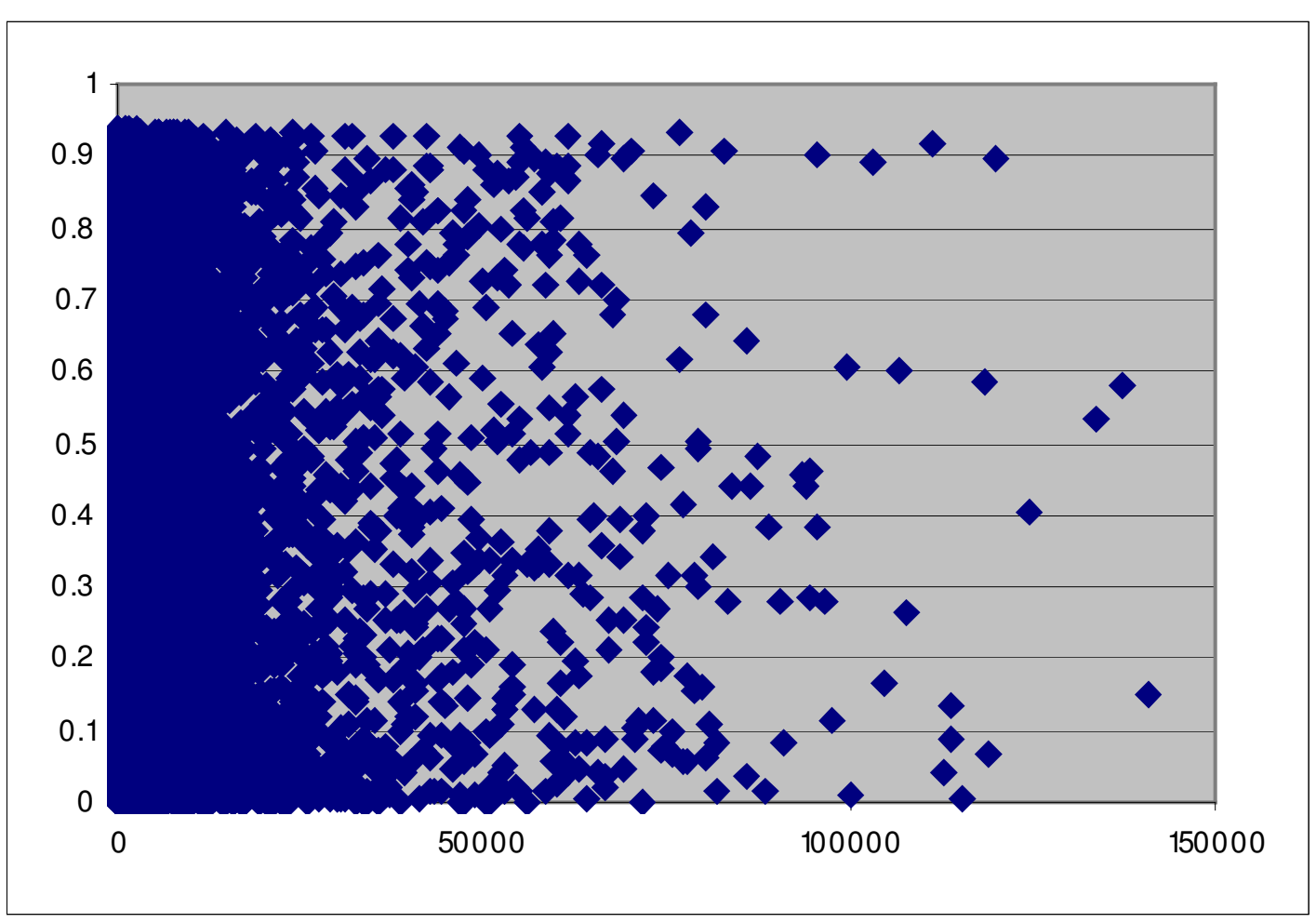

iWAT

Supplementary Figure 4. Relation between p-value (estimated by permutation test). In all three tissues, the mean expression level (raw) is plotted on the abscissa (x axis) and the corresponding *p* value on the ordinate (y axis).
