# Supplementary material for: Circadian Clocks Are Resounding in Peripheral Tissues
Source: PLoS Comput Biol. 2006 Mar 10;2(3):e16. doi: 10.1371/journal.pcbi.0020016 (PMC1391918; doi:10.1371/journal.pcbi.0020016)
Supplement: Supplementary file 6 [file pcbi.0020016.st001.pdf]

KEGGCharts

Show Annotation | Show Chart Data | Open in New Window ☑

PATHWAY	
Circadian rhythm	2.0% (9)
Glycerolipid metabolism	1.1% (5)
Purine metabolism	1.1% (5)
Butanoate metabolism	0.9% (4)
Sterol biosynthesis	0.9% (4)
Synthesis and degradation of ketonebodies	0.9% (4)
Valine, leucine and isoleucinedegradation	0.9% (4)
Citrate cycle (TCA cycle)	0.7% (3)
Fatty acid metabolism	0.7% (3)
Pyruvate metabolism	0.7% (3)
ATP synthesis	0.4% (2)
Aminosugarsmetabolism	0.4% (2)
Glycolysis / Gluconeogenesis	0.4% (2)
Nicotinate andnicotinamide metabolism	0.4% (2)
Oxidative phosphorylation	0.4% (2)
Propanoate metabolism	0.4% (2)
Pyrimidine metabolism	0.4% (2)
Reductive carboxylate cycle (CO2fixation)	0.4% (2)
Terpenoidbiosynthesis	0.4% (2)
Alzheimer's disease	0.2% (1)
Arginine and proline metabolism	0.2% (1)
Basal transcriptionfactors	0.2% (1)
Fructose and mannose metabolism	0.2% (1)
Glutamate metabolism	0.2% (1)
Glutathione metabolism	0.2% (1)
N-Glycansbiosynthesis	0.2% (1)
Nitrogen metabolism	0.2% (1)
Porphyrin andchlorophyll metabolism	0.2% (1)
Prion disease	0.2% (1)
Sphingoglycolipidmetabolism	0.2% (1)
Tyrosine metabolism	0.2% (1)
Glycosylphosphatidylinositol(GPI)-anchor biosynthesis	0.2% (1)
Phosphatidylinositol signaling system	0.2% (1)

Get Unclassifieds	**88.4% (403)**

53 out of 456 uniqids were classified
