# Supplementary material for: Circadian Clocks Are Resounding in Peripheral Tissues
Source: PLoS Comput Biol. 2006 Mar 10;2(3):e16. doi: 10.1371/journal.pcbi.0020016 (PMC1391918; doi:10.1371/journal.pcbi.0020016)
Supplement: Supplementary file 7 [file pcbi.0020016.st002.pdf]

| Affymetrix probesets | Gene identifiers | Gene Name | Gene Symbol | Summary |
|---|---|---|---|---|
| 1415688_at | 67128 | ubiquitin-conjugating enzyme E2G 1 (UBC7 homolog, C. elegans) | UBE2G1;Ube2g1 | [RefSeq Summary:] The modification of proteins with ubiquitin is an important cellular mechanism for targeting abnormal or short-lived proteins for degradation. Ubiquitination involves at least three classes of enzymes: ubiquitin-activating enzymes, or E1s, ubiquitin-conjugating enzymes, or E2s, and ubiquitin-protein ligases, or E3s. This gene encodes a member of the E2 ubiquitin-conjugating enzyme family. The encoded protein shares 98-100% sequence identity with the zebrafish, frog, rat and mouse counterparts, which indicates that this enzyme is highly conserved in eukaryotes. Two alternatively spliced transcript variants encoding distinct isoforms have been found for this gene. |
| 1415708_at | 103674 | expressed sequence AI316828 | AI316828 | |
| 1415721_a_at | 74763 | RIKEN cDNA 1200013P24 gene | 1200013P24Rik | |
| 1415723_at | 217869 | eukaryotic initiation factor 5 (eIF-5);eukaryotic translation initiation factor 5 | EIF5;Eif5 | |
| 1415770_at | 83669 | WD repeat domain 6 | WDR6;Wdr6 | [RefSeq Summary:] This gene encodes a member of the WD repeat protein family. WD repeats are minimally conserved regions of approximately 40 amino acids typically bracketed by gly-his and trp-asp (GH-WD), which may facilitate formation of heterotrimeric or multiprotein complexes. Members of this family are involved in a variety of cellular processes, including cell cycle progression, signal transduction, apoptosis, and gene regulation. This gene is ubiquitously expressed in adult and fetal tissues. |

| | | | | |
|---|---|---|---|---|
| 1415783_at | 65114 | vacuolar protein sorting 35;vacuolar protein sorting 35 (yeast) | VPS35;Vps35 | [RefSeq Summary:] This gene belongs to a group of vacuolar protein sorting (VPS) genes. The encoded protein is a component of a large multimeric complex, termed the retromer complex, involved in retrograde transport of proteins from endosomes to the trans-Golgi network. The close structural similarity between the yeast and human proteins that make up this complex suggests a similarity in function. Expression studies in yeast and mammalian cells indicate that this protein interacts directly with VPS35, which serves as the core of the retromer complex. |
| 1415793_at | 103711 | expressed sequence AI415282 | AI415282 | |
| 1415796_at | 23994 | DAZ associated protein 2 | DAZAP2;Dazap2 | [RefSeq Summary:] In mammals, the Y chromosome directs the development of the testess and plays an important role in spermatogenesis. A high percentage of infertile men have deletions that map to regions of the Y chromosome. The DAZ (deleted in azoospermia) gene cluster maps to the AZFc region and is deleted in many azoospermic and severely oligospermic men. It is thought that the Y chromosomal DAZ gene cluster arose from the transposition, amplification, and pruning of the ancestral autosomal gene DAZL. This gene encodes a RNA-binding protein with two RNP motifs that was originally identified by its interaction with the infertility factors DAZ and DAZL. |
| 1415802_at | 20501 | solute carrier family 16 (monocarboxylic acid transporters), member 1;solute carrier family 16, member 1 | SLC16A1;Slc16a1 | |
| 1415827_a_at | 28146 | stress-associated endoplasmic reticulum protein 1 | D3Ucla1 | |

| | | | | |
|---|---|---|---|---|
| 1415840_at | 68801 | ELOVL family member 5, elongation of long chain fatty acids (FEN1/Elo2, SUR4/Elo3-like, yeast);ELOVL family member 5, elongation of long chain fatty acids (yeast) | ELOVL5;Elovl5 | |
| 1415860_at | 16647 | karyopherin (importin) alpha 2;karyopherin alpha 2 (RAG cohort 1, importin alpha 1) | KPNA2;Kpna2 | [RefSeq Summary:] The import of proteins into the nucleus is a process that involves at least 2 steps. The first is an energy-independent docking of the protein to the nuclear envelope and the second is an energy-dependent translocation through the nuclea |
| 1415892_at | 20397 | sphingosine phosphate lyase 1;sphingosine-1-phosphate lyase 1 | SGPL1;Sgpl1 | |
| 1415899_at | 16477 | Jun-B oncogene;jun B proto-oncogene | JUNB;Junb | |
| 1415909_at | 20867 | stress-induced phosphoprotein 1;stress-induced-phosphoprotein 1 (Hsp70/Hsp90-organizing protein) | STIP1;Stip1 | |
| 1415973_at | 17118 | myristoylated alanine rich protein kinase C substrate;myristoylated alanine-rich protein kinase C substrate | MARCKS;Marcks | [RefSeq Summary:] The protein encoded by this gene is a substrate for protein kinase C. It is localized to the plasma membrane and is an actin filament crosslinking protein. Phosphorylation by protein kinase C or binding to calcium-calmodulin inhibits its association with actin and with the plasma membrane, leading to its presence in the cytoplasm. The protein is thought to be involved in cell motility, phagocytosis, membrane trafficking and mitogenesis. |

| | | | | |
|---|---|---|---|---|
| 1415975_at | 52502 | calcium regulated heat stable protein 1;calcium regulated heat stable protein 1, 24kDa | CARHSP1;Carhsp1 | |
| 1415992_at | 56703 | phosphatidylinositol glycan class O;phosphatidylinositol glycan, class O | PIGO;Pigo | [RefSeq Summary:] This gene encodes a protein that is involved in glycosylphosphatidylinositol (GPI)-anchor biosynthesis. The GPI-anchor is a glycolipid which contains three mannose molecules in its core backbone. The GPI-anchor is found on many blood cells and serves to anchor proteins to the cell surface. This protein is involved in the transfer of ethanolaminephosphate (EtNP) to the third mannose in GPI. At least two alternatively spliced transcripts encoding distinct isoforms have been found for this gene. |
| 1415993_at | 20775 | squalene epoxidase | SQLE;Sqle | [RefSeq Summary:] Squalene epoxidase catalyzes the first oxygenation step in sterol biosynthesis and is thought to be one of the rate-limiting enzymes in this pathway. |
| 1415996_at | 56338 | thioredoxin interacting protein | TXNIP;Txnip | |
| 1416027_at | 18570 | programmed cell death 6 | PDCD6;Pdcd6 | [RefSeq Summary:] This gene encodes a calcium-binding protein belonging to the penta-EF-hand protein family. Calcium binding is important for homodimerization and for conformational changes required for binding to other protein partners. This gene product participates in T cell receptor-, Fas-, and glucocorticoid-induced programmed cell death. In mice deficient for this gene product, however, apoptosis was not blocked suggesting this gene product is functionally redundant. |

| | | | | |
|---|---|---|---|---|
| 1416075_at | 64010 | salvador homolog 1 (Drosophila) | SAV1;Sav1 | [RefSeq Summary:] WW domain-containing proteins are found in all eukaryotes and play an important role in the regulation of a wide variety of cellular functions such as protein degradation, transcription, and RNA splicing. This gene encodes a protein which contains 2 WW domains and a coiled-coil region. It is ubiquitously expressed in adult tissues. The encoded protein is 94% identical to the mouse protein at the amino acid level. |
| 1416125_at | 14229 | FK506 binding protein 5 | FKBP5;Fkbp5 | [RefSeq Summary:] The protein encoded by this gene is a member of the immunophilin protein family, which play a role in immunoregulation and basic cellular processes involving protein folding and trafficking. This encoded protein is a cis-trans prolyl isomerase that binds to the immunosuppressants FK506 and rapamycin. It is thought to mediate calcineurin inhibition. It also interacts functionally with mature hetero-oligomeric progesterone receptor complexes along with the 90 kDa heat shock protein and P23 protein. This gene has been found to have multiple polyadenylation sites. |
| 1416147_at | 15525 | heat shock 70 kDa protein 4;heat shock 70kDa protein 4;heat shock protein 4 | HSPA4;Hspa4 | [Proteome Summary:] Member 4 of the heat shock HSP70 family of molecular chaperones; may act in protein folding, translocation, and assembly into complexes; strongly similar to rat irp94 |
| 1416155_at | 15354 | high mobility group box 3;high-mobility group box 3 | HMGB3;Hmgb3 | |
| 1416156_at | 22330 | vinculin | VCL;Vcl | [RefSeq Summary:] Vinculin is a cytoskeletal protein associated with cell-cell and cell-matrix junctions, where it is thought to function as one of several interacting proteins involved in anchoring F-actin to the membrane. There are two vinculin isoforms: vinculin (VCL) and metavinculin (meta-VCL), which arise via alternative splicing of the vinculin gene. Human vinculin protein exhibits a greater than 95% sequence identity to the chicken vinculin protein. |

| | | | | |
|---|---|---|---|---|
| 1416201_at | 12928 | v-crk sarcoma virus CT10 oncogene homolog (avian) | CRK;Crk | [RefSeq Summary:] This gene encodes a member of an adapter protein family that binds to several tyrosine-phosphorylated proteins. The product of this gene has several SH2 and SH3 domains (src-homology domains) and is involved in several signaling pathways, recruiting cytoplasmic proteins in the vicinity of tyrosine kinase through SH2-phosphotyrosine interaction. The N-terminal SH2 domain of this protein functions as a positive regulator of transformation whereas the C-terminal SH3 domain functions as a negative regulator of transformation. Two alternative transcripts encoding different isoforms with distinct biological activity have been described. |
| 1416222_at | 18194 | NAD(P) dependent steroid dehydrogenase-like | Nsdhl | |
| 1416256_a_at | 22154 | tubulin, beta 5;tubulin, beta, 5 | TUBB5;Tubb5 | [Proteome Summary:] Beta 5-tubulin; polymerizes to form microtubules; member of a family of structural proteins |
| 1416288_at | 15502 | DnaJ (Hsp40) homolog, subfamily A, member 1 | DNAJA1;Dnaja1 | |
| 1416303_at | 56722 | LPS-induced TN factor;LPS-induced TNF-alpha factor;lipopolysaccharide-induced TNF factor | LITAF;Litaf | |
| 1416304_at | 56722 | LPS-induced TN factor;LPS-induced TNF-alpha factor;lipopolysaccharide-induced TNF factor | LITAF;Litaf | |

| | | | | |
|---|---|---|---|---|
| 1416316_at | 26458 | solute carrier family 27 (fatty acid transporter), member 2;solute carrier family 27 (fatty acid transporter), member 32 | SLC27A2;Slc27a2 | [RefSeq Summary:] The protein encoded by this gene is an isozyme of long-chain fatty-acid-coenzyme A ligase family. Although differing in substrate specificity, subcellular localization, and tissue distribution, all isozymes of this family convert free long-chain fatty acids into fatty acyl-CoA esters, and thereby play a key role in lipid biosynthesis and fatty acid degradation. This isozyme activates long-chain, branched-chain and very-long-chain fatty acids containing 22 or more carbons to their CoA derivatives. It is expressed primarily in liver and kidney, and is present in both endoplasmic reticulum and peroxisomes but not in mitochondria. Its decreased peroxisomal enzyme activity is in part responsible for the biochemical pathology in X-linked adrenoleukodystrophy. |
| 1416332_at | 12696 | cold inducible RNA binding protein;cold inducible RNA-binding protein | CIRBP;Cirbp | |
| 1416362_a_at | 14228 | FK506 binding protein 4;FK506 binding protein 4 (59 kDa);FK506 binding protein 4, 59kDa | FKBP4;Fkbp4 | [RefSeq Summary:] The protein encoded by this gene is a member of the immunophilin protein family, which play a role in immunoregulation and basic cellular processes involving protein folding and trafficking. This encoded protein is a cis-trans prolyl iso |
| 1416364_at | 15516 | heat shock 90kDa protein 1, beta;heat shock protein 1, beta | HSPCB;Hspcb | |
| 1416432_at | 170768 | 6-phosphofructo-2-kinase/fructose-2,6-biphosphatase 3 | PFKFB3;Pfkfb3 | |
| 1416440_at | 53599 | CD164 antigen;CD164 antigen, sialomucin | CD164;Cd164 | [Proteome  Summary:] Sialomucin; mucin-like cell surface glycoprotein that facilitates adhesion of CD34+ cells, plays a role in regulating hematopoietic cell proliferation |

| | | | | |
|---|---|---|---|---|
| 1416488_at | 12452 | cyclin G2 | CCNG2;Ccng2 | [RefSeq Summary:] The eukaryotic cell cycle is governed by cyclin-dependent protein kinases (CDKs) whose activities are regulated by cyclins and CDK inhibitors. The 8 species of cyclins reported in mammals, cyclins A through H, share a conserved amino acid sequence of about 90 residues called the cyclin box. The amino acid sequence of cyclin G is well conserved among mammals. The nucleotide sequence of cyclin G1 and cyclin G2 are 53% identical. Unlike cyclin G1, cyclin G2 contains a C-terminal PEST protein destabilization motif, suggesting that cyclin G2 expression is tightly regulated through the cell cycle. |
| 1416530_a_at | 18950 | purine-nucleoside phosphorylase | Pnp | |
| 1416555_at | 13663 | etoposide induced 2.4 mRNA | EI24;Ei24 | |
| 1416591_at | 19376 | RAB34, member RAS oncogene family;RAB34, member of RAS oncogene family | RAB34;Rab34 | |
| 1416592_at | 93692 | glutaredoxin 1 (thioltransferase) | Glrx1 | |
| 1416593_at | 93692 | glutaredoxin 1 (thioltransferase) | Glrx1 | |
| 1416639_at | 56485 | solute carrier family 2 (facilitated glucose transporter), member 5;solute carrier family 2 (facilitated glucose/fructose transporter), member 5;solute carrier family 2, member 5 | SLC2A5;Slc2a5 | |

| | | | | |
|---|---|---|---|---|
| 1416699_at | 67388 | RIKEN cDNA 1110008F13 gene | 1110008F13Rik | |
| 1416743_at | 107652 | UDP-N-acetylglucosamine pyrophosphorylase 1;UDP-N-acteylglucosamine pyrophosphorylase 1 | UAP1;Uap1 | |
| 1416750_at | 18391 | opioid receptor, sigma 1;type I sigma receptor | OPRS1;Oprs1 | [RefSeq Summary:] This gene encodes a receptor protein that interacts with a variety of psychotomimetic drugs, including cocaine and amphetamines. The receptor is believed to play an important role in the cellular functions of various tissues associated with the endocrine, immune, and nervous systems. Alternative splicing has been observed for this gene and five transcript variants, each encoding a distinct protein, have been identified. |
| 1416754_at | 19085 | protein kinase, cAMP dependent regulatory, type I beta;protein kinase, cAMP dependent regulatory, type I, beta;protein kinase, cAMP-dependent, regulatory, type I, beta | PRKAR1B;Prkar1b | |
| 1416773_at | 22390 | WEE1 homolog (S. pombe);wee 1 homolog (S. pombe) | WEE1;Wee1 | [RefSeq Summary:] This gene encodes a nuclear protein, which is a tyrosine kinase belonging to the Ser/Thr family of protein kinases. This protein catalyzes the inhibitory tyrosine phosphorylation of CDC2/cyclin B kinase, and appears to coordinate the transition between DNA replication and mitosis by protecting the nucleus from cytoplasmically activated CDC2 kinase. |

| | | | | |
|---|---|---|---|---|
| 1416774_at | 22390 | WEE1 homolog (S. pombe);wee 1 homolog (S. pombe) | WEE1;Wee1 | [RefSeq Summary:] This gene encodes a nuclear protein, which is a tyrosine kinase belonging to the Ser/Thr family of protein kinases. This protein catalyzes the inhibitory tyrosine phosphorylation of CDC2/cyclin B kinase, and appears to coordinate the transition between DNA replication and mitosis by protecting the nucleus from cytoplasmically activated CDC2 kinase. |
| 1416794_at | 56298 | ADP-ribosylation-like factor 6 interacting protein 2 | ARL6IP2;Arl6ip2 | |
| 1416904_at | 56758 | muscleblind-like (Drosophila) | MBNL1;Mbnl1 | |
| 1416922_a_at | 12177 | BCL2/adenovirus E1B 19 kDa-interacting protein 3-like;BCL2/adenovirus E1B 19kDa interacting protein 3-like;BCL2/adenovirus E1B 19kDa-interacting protein 3-like | BNIP3L;Bnip3l | [RefSeq Summary:] This gene is a member of the BCL2/adenovirus E1B 19 kd-interacting protein (BNIP) family. It interacts with the E1B 19 kDa protein which is responsible for the protection of virally-induced cell death, as well as E1B 19 kDa-like sequences of BCL2, also an apoptotic protector. The protein encoded by this gene is a functional homolog of BNIP3, a proapoptotic protein. This protein may function simultaneously with BNIP3 and may play a role in tumor suppression. |
| 1416924_at | 55950 | brain protein I3 | BRI3;Bri3 | |
| 1416933_at | 18984 | P450 (cytochrome) oxidoreductase | POR;Por | |
| 1416953_at | 14219 | connective tissue growth factor | CTGF;Ctgf | |
| 1416958_at | 21833 | nuclear receptor subfamily 1, group D, member 1;thyroid hormone receptor alpha;thyroid hormone receptor, alpha (erythroblastic leukemia viral (v-erb-a) oncogene homolog, avian) | NR1D1;THRA;Thra | [RefSeq Summary:] The protein encoded by this gene is a nuclear hormone receptor for triiodothyronine. It is one of the several receptors for thyroid hormone, and has been shown to mediate the biological activities of thyroid hormone. Knockout studies in mice suggest that the different receptors, while having certain extent of redundancy, may mediate different functions of thyroid hormone. Alternatively spliced transcript variants encoding distinct isoforms have been reported. |

| | | | | |
|---|---|---|---|---|
| 1417013_at | 80888 | crystallin, alpha C;protein kinase H11 | HSPB8;Hspb8 | |
| 1417027_at | 80890 | tripartite motif protein 2;tripartite motif-containing 2 | TRIM2;Trim2 | [RefSeq Summary:] The protein encoded by this gene is a member of the tripartite motif (TRIM) family. The TRIM motif includes three zinc-binding domains, a RING, a B-box type 1 and a B-box type 2, and a coiled-coil region. The protein localizes to cytoplasmic filaments. Its function has not been identified. |
| 1417028_a_at | 80890 | tripartite motif protein 2;tripartite motif-containing 2 | TRIM2;Trim2 | [RefSeq Summary:] The protein encoded by this gene is a member of the tripartite motif (TRIM) family. The TRIM motif includes three zinc-binding domains, a RING, a B-box type 1 and a B-box type 2, and a coiled-coil region. The protein localizes to cytoplasmic filaments. Its function has not been identified. |
| 1417065_at | 13653 | early growth response 1 | EGR1;Egr1 | [RefSeq Summary:] The protein encoded by this gene belongs to the EGR family of C2H2-type zinc-finger proteins. It is a nuclear protein and functions as a transcriptional regulator. The products of target genes it activates are required for differentitation and mitogenesis. Studies suggest this is a cancer suppresor gene. |
| 1417066_at | 67426 | chaperone, ABC1 activity of bc1 complex like (S. pombe) | CABC1;Cabc1 | [RefSeq Summary:] CABC1 is one of several genes induced by p53 (MIM 191170) expression. The S. pombe homolog, ABC1, encodes a chaperone-like protein essential for the proper conformation and functioning of protein complexes in the respiratory chain.[supplied by OMIM] |
| 1417130_s_at | 57875 | angiopoietin-like 4 | ANGPTL4;Angptl4 | [RefSeq Summary:] Peroxisome proliferator-activated receptor gamma is a nuclear receptor which regulates adipose differentiation and glucose homeostasis. It acts through ligand-dependent transcriptional activation of target genes. This gene is one of the targets of peroxisome proliferator-activated receptor gamma and encodes an angiopoietin-like secreted glycoprotein. This gene also has been referred to as ANGPTL2, although a different gene exists with that same designation. Two transcript variants encoding the same protein have been found for this gene. |

| | | | | |
|---|---|---|---|---|
| 1417146_at | 67873 | RIKEN cDNA 2410018C20 gene;eukaryotic translation initiation factor 2B, subunit 1 (alpha);eukaryotic translation initiation factor 2B, subunit 1 alpha, 26kDa;hypothetical protein MGC3207;translation initiation factor eIF-2B alpha-subunit | 2410018C20Rik;EIF2B1;Eif2b1;MGC3207 | |
| 1417168_a_at | 53376 | ubiquitin specific protease 2 | USP2;Usp2 | |
| 1417169_at | 53376 | ubiquitin specific protease 2 | USP2;Usp2 | |
| 1417190_at | 59027 | pre-B-cell colony-enhancing factor | Pbef | |
| 1417212_at | 68241 | RIKEN cDNA 9530058B02 gene | 9530058B02Rik | |
| 1417273_at | 27273 | pyruvate dehydrogenase kinase, isoenzyme 4;pyruvate dehydrogenate kinase 4 | PDK4;Pdk4 | |
| 1417339_a_at | 56455 | dynein, cytoplasmic, light chain 1 | Dnclc1 | |
| 1417406_at | 55942 | RIKEN cDNA 1110032C13 gene | 1110032C13Rik | |
| 1417496_at | 12870 | ceruloplasmin;ceruloplasmin (ferroxidase) | CP;Cp | [RefSeq Summary:] Ceruloplasmin is a plasma metalloprotein that that binds most of the copper in plasma. Human ceruloplasmin is composed of a single polypeptide chain. Ceruloplasmin deficiency leads to iron accumulation and cuases damages to a varieties of tissues and organs. |

| | | | | |
|---|---|---|---|---|
| 1417506_at | 57441 | geminin;geminin, DNA replication inhibitor | GMNN;Gmnn | |
| 1417602_at | 18627 | period homolog 1 (Drosophila);period homolog 2;period homolog 2 (Drosophila);period homolog 3 (Drosophila) | PER1;PER2;PER3;Per1;Per2;Per3 | [RefSeq Summary:] This gene is a member of the Period family of genes and is expressed in a circadian pattern in the suprachiasmatic nucleus, the primary circadian pacemaker in the mammalian brain. Genes in this family encode components of the circadian r |
| 1417603_at | 18627 | period homolog 1 (Drosophila);period homolog 2;period homolog 2 (Drosophila);period homolog 3 (Drosophila) | PER1;PER2;PER3;Per1;Per2;Per3 | [RefSeq Summary:] This gene is a member of the Period family of genes and is expressed in a circadian pattern in the suprachiasmatic nucleus, the primary circadian pacemaker in the mammalian brain. Genes in this family encode components of the circadian r |
| 1417622_at | 20496 | solute carrier family 12 (sodium/potassium/chloride transporters), member 2;solute carrier family 12, member 2 | SLC12A2;Slc12a2 | |
| 1417713_at | 67204 | eukaryotic translation initiation factor 2, subunit 2 (beta);eukaryotic translation initiation factor 2, subunit 2 beta, 38kDa;similar to translation initiation factor eIF-2 beta chain - rabbit | EIF2S2;Eif2s2 | [RefSeq Summary:] Eukaryotic translation initiation factor 2 (EIF-2) functions in the early steps of protein synthesis by forming a ternary complex with GTP and initiator tRNA and binding to a 40S ribosomal subunit. EIF-2 is composed of three subunits, alpha, beta, and gamma, with the protein encoded by this gene representing the beta subunit. The beta subunit catalyzes the exchange of GDP for GTP, which recycles the EIF-2 complex for another round of initiation. |
| 1417766_at | 66427 | cytochrome b5 outer mitochondrial membrane precursor | 1810044O22Rik | |

| | | | | |
|---|---|---|---|---|
| 1417767_at | 66427 | cytochrome b5 outer mitochondrial membrane precursor | 1810044O22Rik | |
| 1417839_at | 12741 | claudin 5;claudin 5 (transmembrane protein deleted in velocardiofacial syndrome) | CLDN5;Cldn5 | [RefSeq Summary:] This gene encodes a member of the claudin family. Claudins are integral membrane proteins and components of tight junction strands. Tight junction strands serve as a physical barrier to prevent solutes and water from passing freely through the paracellular space between epithelial or endothelial cell sheets. Mutations in this gene have been found in patients with velocardiofacial syndrome. |
| 1417870_x_at | 64138 | cathepsin Z | CTSZ;Ctsz | [RefSeq Summary:] The protein encoded by this gene is a lysosomal cysteine proteinase and member of the peptidase C1 family. It exhibits both carboxy-monopeptidase and carboxy-dipeptidase activities. The encoded protein has also been known as cathepsin X and cathepsin P. This gene is expressed ubiquitously in cancer cell lines and primary tumors and, like other members of this family, may be involved in tumorigenesis. At least two transcript variants of this gene have been found, but the full-length nature of only one of them has been determined. |
| 1417941_at | 67311 | RIKEN cDNA 1600031M04 gene | 1600031M04Rik | |
| 1417998_at | 56351 | telomerase binding protein, p23 | 5730442A20Rik | |
| 1418171_at | 66684 | RIKEN cDNA 3930402F23 gene | 3930402F23Rik | |
| 1418174_at | 13170 | D site albumin promoter binding protein;D site of albumin promoter (albumin D-box) binding protein | DBP;Dbp | |

| | | | | |
|---|---|---|---|---|
| 1418253_a_at | 18415 | osmotic stress protein;similar to osmotic stress protein 94 kDa | OSP94;Osp94 | |
| 1418300_a_at | 17347 | MAP kinase-interacting serine/threonine kinase 2 | MKNK2;Mknk2 | |
| 1418322_at | 12916 | cAMP responsive element modulator | CREM;Crem | [RefSeq Summary:] This gene encodes a bZIP transcription factor that binds to the cAMP responsive element found in many viral and cellular promoters. It is an important component of cAMP-mediated signal transduction during the spermatogenetic cycle, as well as other complex processes. Alternative promoter and translation initiation site usage allows this gene to exert spatial and temporal specificity to cAMP responsiveness. Multiple alternatively spliced transcript variants encoding several different isoforms have been found for this gene, with some of them functioning as activators and some as repressors of transcription. |
| 1418443_at | 103573 | exportin 1 (CRM1 homolog, yeast);exportin 1 (CRM1, yeast, homolog);exportin 1, CRM1 homolog (yeast) | XPO1;Xpo1 | [RefSeq Summary:]  Exportin 1 mediates leucine-rich nuclear export signal (NES)-dependent protein transport. XPO1 specifically inhibits the nuclear export of Rev and U snRNAs.  It is also involved in the control of several cellular processes by controlling the localization of cyclin B, MPAK, and MAPKAP kinase 2.  XPO1 also regulates NFAT and AP-1. |
| 1418553_at | 102098 | RIKEN cDNA D030053O22 gene;Rho-specific guanine nucleotide exchange factor p114 | ARHGEF18;Arhgef18 | [RefSeq Summary:] Rho GTPases are GTP binding proteins that regulate a wide spectrum of cellular functions. These cellular processes include cytoskeletal rearrangements, gene transcription, cell growth and motility. Activation of Rho GTPases is under the direct control of guanine nucleotide exchange factors (GEFs). The protein encoded by this gene is a guanine nucleotide exchange factor and belongs to the Rho GTPase GFE family. Family members share a common feature, a Dbl (DH) homology domain followed by a pleckstrin (PH) homology domain. |

| | | | | |
|---|---|---|---|---|
| 1418586_at | 11515 | adenylate cyclase 1 (brain);adenylate cyclase 2 (brain);adenylate cyclase 3;adenylate cyclase 4;adenylate cyclase 5;adenylate cyclase 6;adenylate cyclase 7;adenylate cyclase 8;adenylate cyclase 8 (brain);adenylate cyclase 9;adenylyl cyclase 2;adenylyl cyclase 4;adenylyl cyclase 6;adenylyl cyclase 8;testicular soluble adenylyl cyclase | ADCY1;ADCY2;ADCY3;ADCY4;ADCY5;ADCY6;ADCY7;ADCY8;ADCY9;Adcy2;Adcy3;Adcy4;Adcy5;Adcy6;Adcy7;Adcy8;Adcy9;SAC | [RefSeq Summary:] Adenylate cyclase is a membrane bound enzyme that catalyses the formation of cyclic AMP from ATP. It is regulated by a family of G protein-coupled receptors, protein kinases, and calcium. The type 9 adenylyl cyclase (AC9) is a widely dis |
| 1418592_at | 58233 | DnaJ (Hsp40) homolog, subfamily A, member 4 | DNAJA4;Dnaja4 | |

| | | | | |
|---|---|---|---|---|
| 1418659_at | 12753 | circadian locomoter output cycles kaput;clock gene;clock homolog (mouse);neuronal PAS domain protein 2 | CLOCK;Clock;NPAS2;Npas2 | [RefSeq Summary:] The protein encoded by this gene is a member of the basic helix-loop-helix (bHLH)-PAS family of transcription factors. A similar mouse protein may play a regulatory role in the acquisition of specific types of memory. It also may function as a part of a molecular clock operative in the mammalian forebrain. ;[RefSeq Summary:] This gene encodes a protein that belongs to the basic helix-loop-helix (bHLH) family of transcription factors. Polymorphisms within the encoded protein have been associated with circadian rhythm sleep disorders. A similar protein in mice is a circadian regulator that acts as a transcription factor and forms a heterodimer with aryl hydrocarbon receptor nuclear translocator-like to activate transcription of mouse period 1. |
| 1418695_a_at | 74287 | potassium channel modulatory factor 1 | KCMF1;Kcmf1 | |
| 1418697_at | 21743 | thioether S-methyltransferase | Temt | |
| 1418815_at | 12558 | cadherin 2;cadherin 2, type 1, N-cadherin (neuronal) | CDH2;Cdh2 | [RefSeq Summary:] This gene is a classical cadherin from the cadherin superfamily. The encoded protein is a calcium dependent cell-cell adhesion glycoprotein comprised of five extracellular cadherin repeats, a transmembrane region and a highly conserved cytoplasmic tail. The protein functions during gastrulation and is required for establishment of left-right asymmetry. At certain central nervous system synapses, presynaptic to postsynaptic adhesion is mediated at least in part by this gene product. |
| 1418819_at | 67166 | RIKEN cDNA 2610313E07 gene | 2610313E07Rik | |
| 1418932_at | 18030 | nuclear factor, interleukin 3 regulated;nuclear factor, interleukin 3, regulated | NFIL3;Nfil3 | |

| | | | | |
|---|---|---|---|---|
| 1418982_at | 12606 | CCAAT/enhancer binding protein (C/EBP), alpha;CCAAT/enhancer binding protein, alpha | CEBPA;Cebpa | [RefSeq Summary:] The protein encoded by this intronless gene is a bZIP transcription factor which can bind as a homodimer to certain promoters and enhancers. It can also form heterodimers with the related proteins CEBP-beta and CEBP-gamma. The encoded protein has been shown to bind to the promoter and modulate the expression of the gene encoding leptin, a protein that plays an important role in body weight homeostasis. Also, the encoded protein can interact with CDK2 and CDK4, thereby inhibiting these kinases and causing growth arrest in cultured cells. |
| 1419029_at | 50527 | ERO1-like (S. cerevisiae) | ERO1L;Ero1l | [RefSeq Summary:] This gene encodes a protein similar to a Saccharomyces cerevisiae protein which is required for oxidative protein folding. A similar human protein is an integral membrane protein in the endoplasmic reticulum (ER) and is thought to be involved in oxidative ER protein folding. |
| 1419070_at | 12879 | cystin 1 | CYS1;Cys1 | |
| 1419104_at | 66082 | RIKEN cDNA 0610041D24 gene;abhydrolase domain containing 6 | ABHD6;Abhd6 | |
| 1419163_s_at | 19107 | DnaJ (Hsp40) homolog, subfamily C, member 3 | DNAJC3;Dnajc3 | [RefSeq Summary:] The protein encoded by this gene contains multiple tetratricopeptide repeat (TPR) motifs as well as the highly conserved J domain found in DNAJ chaperone family members. It is a member of the tetratricopeptide repeat family of proteins and acts as an inhibitor of the interferon-induced, dsRNA-activated protein kinase (PKR). |
| 1419182_at | 64817 | likely ortholog of mouse polydom;polydomain protein | POLYDOM;Polydom | |

| | | | | |
|---|---|---|---|---|
| 1419185_a_at | 58805 | Williams Beuren syndrome chromosome region 14;Williams-Beuren syndrome chromosome region 14 homolog (human) | WBSCR14;Wbscr14 | [RefSeq Summary:] This gene encodes a basic helix-loop-helix leucine zipper transcription factor of the Myc/Max/Mad superfamily. It may play a role in cell proliferation and/or differentiation. It is deleted in Williams-Beuren syndrome, a multisystem developmental disorder caused by the deletion of contiguous genes at chromosome 7q11.23. This gene contains 17 exons; its alternative splicing generates 5 transcript variants. |
| 1419273_at | 19777 | RPB5-mediating protein | C80913 | |
| 1419450_at | 66612 | ORM1-like 3 (S. cerevisiae) | ORMDL3;Ormdl3 | |
| 1419509_a_at | 56174 | N-acetylglucosamine kinase | NAGK;Nagk | [RefSeq Summary:] N-acetylglucosamine kinase (NAGK; EC 2.7.1.59) converts endogenous N-acetylglucosamine (GlcNAc), a major component of complex carbohydrates, from lysosomal degradation or nutritional sources into GlcNAc 6-phosphate. NAGK belongs to the group of N-acetylhexosamine kinases and is a prominent salvage enzyme of amino sugar metabolism in mammals.[supplied by OMIM] |
| 1419544_at | 66335 | ATPase, H+ transporting, V1 subunit C, isoform 1;ATPase, H+ transporting, lysosomal 42kDa, V1 subunit C, isoform 1 | ATP6V1C1;Atp6v1c1 | [RefSeq Summary:] This gene encodes a component of vacuolar ATPase (V-ATPase), a multisubunit enzyme that mediates acidification of eukaryotic intracellular organelles. V-ATPase dependent organelle acidification is necessary for such intracellular processes as protein sorting, zymogen activation, and receptor-mediated endocytosis. V-ATPase is comprised of a cytosolic V1 domain and a transmembrane V0 domain. The V1 domain consists of a hexamer of three A and three B subunits plus the C, D, and E subunits. It contains the ATP catalytic site. The encoded protein is known as the C subunit and is found ubiquitously. This C subunit is analogous but not homologous to gamma subunit of F-ATPases. Transcript variants derived from alternative polyadenylation exist. Previously, this gene was designated ATP6D. |
| 1419650_at | 22763 | zinc finger RNA binding protein | ZFR;Zfr | |

| | | | | |
|---|---|---|---|---|
| 1419666_x_at | 56312 | nuclear protein 1 | Nupr1 | |
| 1419748_at | 26874 | ATP-binding cassette, sub-family D (ALD), member 2 | ABCD2;Abcd2 | [RefSeq Summary:] The membrane-associated protein encoded by this gene is a member of the superfamily of ATP-binding cassette (ABC) transporters. ABC proteins transport various molecules across extra- and intra-cellular membranes. ABC genes are divided in |
| 1420013_s_at | 16987 | 2,3-oxidosqualene: lanosterol cyclase;lanosterol synthase;lanosterol synthase (2,3-oxidosqualene-lanosterol cyclase) | LSS;Lss | |
| 1420093_s_at | 50926 | heterogeneous nuclear ribonucleoprotein D-like | HNRPDL;Hnrpdl | [RefSeq Summary:] This gene belongs to the subfamily of ubiquitously expressed heterogeneous nuclear ribonucleoproteins (hnRNPs). The hnRNPs are RNA binding proteins and they complex with heterogeneous nuclear RNA (hnRNA). These proteins are associated with pre-mRNAs in the nucleus and appear to influence pre-mRNA processing and other aspects of mRNA metabolism and transport. While all of the hnRNPs are present in the nucleus, some seem to shuttle between the nucleus and the cytoplasm. The hnRNP proteins have distinct nucleic acid binding properties. The protein encoded by this gene has two RRM domains that bind to RNAs. Two alternatively spliced transcript variants have been described for this gene. The variants encode the same protein but have different 3' UTRs. This protein is similar to its family member HNRPD. |
| 1420131_s_at | 108705 | RIKEN cDNA 1810010L20 gene | 1810010L20Rik | |

| | | | | |
|---|---|---|---|---|
| 1420138_at | 20509 | solute carrier family 19 (folate transporter), member 1;solute carrier family 19 (sodium/hydrogen exchanger), member 1;solute carrier family 19, member 1 | SLC19A1;Slc19a1 | [RefSeq Summary:] Transport of folate compounds into mammalian cells can occur via receptor-mediated (see MIM 136430) or carrier-mediated mechanisms. A functional coordination between these 2 mechanisms has been proposed to be the method of folate uptake in certain cell types. Methotrexate (MTX) is an antifolate chemotherapeutic agent that is actively transported by the carrier-mediated uptake system. RFC1 plays a role in maintaining intracellular concentrations of folate.[supplied by OMIM] |
| 1420150_at | 74646 | RIKEN cDNA 4930422J18 gene | 4930422J18Rik | |
| 1420376_a_at | 15081 | H3 histone, family 3B;H3 histone, family 3B (H3.3B) | H3F3B;H3f3b | [RefSeq Summary:] Histones are basic nuclear proteins that are responsible for the nucleosome structure of the chromosomal fiber in eukaryotes. Two molecules of each of the four core histones (H2A, H2B, H3, and H4) form an octamer, around which approximately 146 bp of DNA is wrapped in repeating units, called nucleosomes. The linker histone, H1, interacts with linker DNA between nucleosomes and functions in the compaction of chromatin into higher order structures. This gene contains introns and its mRNA is poyadenylated, unlike most histone genes. The protein encoded is a member of the histone H3 family. |
| 1420478_at | 53605 | nucleosome assembly protein 1-like 1 | NAP1L1;Nap1l1 | [RefSeq Summary:] This gene encodes a member of the nucleosome assembly protein (NAP) family. This protein participates in DNA replication and may play a role in modulating chromatin formation and contribute to the regulation of cell proliferation. Alternative splicing of this gene results in several transcript variants; however, not all have been fully described. |
| 1420502_at | 20229 | spermidine/spermine N1-acetyl transferase;spermidine/spermine N1-acetyltransferase | SAT;Sat1 | |

| | | | | |
|---|---|---|---|---|
| 1420622_a_at | 15481 | heat shock 70kDa protein 8;heat shock protein 8 | HSPA8;Hspa8 | [RefSeq Summary:] The product encoded by this gene belongs to the heat shock protein 70 family which contains both heat-inducible and constitutively expressed members. The latter are called heat-shock cognate proteins. This gene encodes a heat-shock cognate protein. This protein binds to nascent polypeptides to facilitate correct folding. It also functions as an ATPase in the disassembly of clathrin-coated vesicles during transport of membrane components through the cell. Two alternatively spliced variants have been characterized to date. |
| 1420623_x_at | 15481 | heat shock 70kDa protein 8;heat shock protein 8 | HSPA8;Hspa8 | [RefSeq Summary:] The product encoded by this gene belongs to the heat shock protein 70 family which contains both heat-inducible and constitutively expressed members. The latter are called heat-shock cognate proteins. This gene encodes a heat-shock cognate protein. This protein binds to nascent polypeptides to facilitate correct folding. It also functions as an ATPase in the disassembly of clathrin-coated vesicles during transport of membrane components through the cell. Two alternatively spliced variants have been characterized to date. |
| 1420722_at | 12686 | elongation of very long chain fatty acids (FEN1/Elo2, SUR4/Elo3, yeast)-like 3 | ELOVL3;Elovl3 | |
| 1420772_a_at | 14605 | glucocorticoid-induced leucine zipper | Dsip1 | |
| 1420808_at | 27057 | nuclear receptor coactivator 4 | NCOA4;Ncoa4 | [Proteome  Summary:] Binds and activates androgen receptor (AR) in ligand-dependent manner |

| | | | | |
|---|---|---|---|---|
| 1420816_at | 22628 | 3-monooxgenase/tryptophan 5-monooxgenase activation protein, gamma polypeptide;tyrosine 3-monooxgenase/tryptophan 5-monooxgenase activation protein, gamma polypeptide;tyrosine 3-monooxygenase/tryptophan 5-monooxygenase activation protein, gamma polypeptide | YWHAG;Ywhag | [RefSeq Summary:] This gene product belongs to the 14-3-3 family of proteins which mediate signal transduction by binding to phosphoserine-containing proteins. This highly conserved protein family is found in both plants and mammals, and this protein is 100% identical to the rat ortholog. It is induced by growth factors in human vascular smooth muscle cells, and is also highly expressed in skeletal and heart muscles, suggesting an important role for this protein in muscle tissue. It has been shown to interact with RAF1 and protein kinase C, proteins involved in various signal transduction pathways. |
| 1420878_a_at | 54401 | tyrosine 3-monooxgenase/tryptophan 5 monooxgenase activation protein, beta polypeptide;tyrosine 3-monooxygenase/tryptophan 5-monooxygenase activation protein, beta polypeptide | YWHAB;Ywhab | [RefSeq Summary:] This gene encodes a protein belonging to the 14-3-3 family of proteins, members of which mediate signal transduction by binding to phosphoserine-containing proteins. This highly conserved protein family is found in both plants and mammals. The encoded protein has been shown to interact with RAF1 and CDC25 phosphatases, suggesting that it may play a role in linking mitogenic signaling and the cell cycle machinery. Two transcript variants, which encode the same protein, have been identified for this gene. |
| 1420880_a_at | 54401 | tyrosine 3-monooxgenase/tryptophan 5 monooxgenase activation protein, beta polypeptide;tyrosine 3-monooxygenase/tryptophan 5-monooxygenase activation protein, beta polypeptide | YWHAB;Ywhab | [RefSeq Summary:] This gene encodes a protein belonging to the 14-3-3 family of proteins, members of which mediate signal transduction by binding to phosphoserine-containing proteins. This highly conserved protein family is found in both plants and mammals. The encoded protein has been shown to interact with RAF1 and CDC25 phosphatases, suggesting that it may play a role in linking mitogenic signaling and the cell cycle machinery. Two transcript variants, which encode the same protein, have been identified for this gene. |

| | | | | |
|---|---|---|---|---|
| 1420940_x_at | 19737 | regulator of G-protein signaling 5;regulator of G-protein signalling 5 | RGS5;Rgs5 | [RefSeq Summary:] The regulator of G protein signaling (RGS) proteins are signal transduction molecules that have structural homology to SST2 of Saccharomyces cerevisiae and EGL-10 of Caenorhabditis elegans. Multiple genes homologous to SST2 are present in higher eukaryotes. RGS proteins are involved in the regulation of heterotrimeric G proteins by acting as GTPase activators.[supplied by OMIM] |
| 1421037_at | 18143 | circadian locomoter output cycles kaput;clock homolog (mouse);neuronal PAS domain protein 2 | CLOCK;Clock;NPAS2;Npas2 | [RefSeq Summary:] The protein encoded by this gene is a member of the basic helix-loop-helix (bHLH)-PAS family of transcription factors. A similar mouse protein may play a regulatory role in the acquisition of specific types of memory. It also may function as a part of a molecular clock operative in the mammalian forebrain. ;[RefSeq Summary:] This gene encodes a protein that belongs to the basic helix-loop-helix (bHLH) family of transcription factors. Polymorphisms within the encoded protein have been associated with circadian rhythm sleep disorders. A similar protein in mice is a circadian regulator that acts as a transcription factor and forms a heterodimer with aryl hydrocarbon receptor nuclear translocator-like to activate transcription of mouse period 1. |
| 1421086_at | 18628 | period homolog 1 (Drosophila);period homolog 2;period homolog 2 (Drosophila);period homolog 3 (Drosophila) | PER1;PER2;PER3;Per1;Per2;Per3 | [RefSeq Summary:] This gene is a member of the Period family of genes and is expressed in a circadian pattern in the suprachiasmatic nucleus, the primary circadian pacemaker in the mammalian brain. Genes in this family encode components of the circadian r |
| 1421087_at | 18628 | period homolog 1 (Drosophila);period homolog 2;period homolog 2 (Drosophila);period homolog 3 (Drosophila) | PER1;PER2;PER3;Per1;Per2;Per3 | [RefSeq Summary:] This gene is a member of the Period family of genes and is expressed in a circadian pattern in the suprachiasmatic nucleus, the primary circadian pacemaker in the mammalian brain. Genes in this family encode components of the circadian r |

| | | | | |
|---|---|---|---|---|
| 1421217_a_at | 16859 | Lectin, galactose binding, soluble 9 (Galectin-9);lectin, galactose binding, soluble 9;lectin, galactoside-binding, soluble, 9 (galectin 9) | LGALS9;Lgals9 | [RefSeq Summary:] The galectins are a family of beta-galactoside-binding proteins implicated in modulating cell-cell and cell-matrix interactions. The protein encoded by this gene is an S-type lectin. This galectin is strongly overexpressed in Hodgkin's disease tissue and it might participate in the interaction between the H&RS cells with their surrounding cells and might thus play a role in the pathogenesis of this disease and/or its consistently associated immunodeficiency. The protein has N- and C- terminal carbohydrate-binding domains connected by a link peptide. Two isoforms (long and short) exist. |
| 1421225_a_at | 54403 | solute carrier family 4 (anion exchanger), member 4;solute carrier family 4, member 4;solute carrier family 4, sodium bicarbonate cotransporter, member 4 | SLC4A4;Slc4a4 | [Proteome Summary:] Sodium bicarbonate cotransporter 4 |
| 1421681_at | 83961 | neuregulin 4 | Nrg4 | |
| 1421743_a_at | 18521 | poly(rC) binding protein 2 | PCBP2;Pcbp2 | [RefSeq Summary:] The protein encoded by this gene appears to be multifunctional. It along with PCBP-1 and hnRNPK corresponds to the major cellular poly(rC)-binding proteins. It contains three K-homologous (KH) domains which may be involved in RNA binding |
| 1421918_at | 11737 | acidic (leucine-rich) nuclear phosphoprotein 32 family, member A;acidic nuclear phosphoprotein 32 family, member A | ANP32A;Anp32a | |
| 1422017_s_at | 97820 | RIKEN cDNA 4833439L19 gene | 4833439L19Rik | |

| | | | | |
|---|---|---|---|---|
| 1422341_s_at | 192654 | lysophospholipase 3;lysophospholipase 3 (lysosomal phospholipase A2) | LYPLA3;Lypla3 | [RefSeq Summary:] Lysophospholipases are enzymes that act on biological membranes to regulate the multifunctional lysophospholipids. The protein encoded by this gene hydrolyzes lysophosphatidylcholine to glycerophosphorylcholine and a free fatty acid. This enzyme is present in the plasma and thought to be associated with high-density lipoprotein. A later paper contradicts the function of this gene. It demonstrates that this gene encodes a lysosomal enzyme instead of a lysophospholipase and has both calcium-independent phospholipase A2 and transacylase activities. |
| 1422470_at | 12176 | BCL2/adenovirus E1B 19 kDa-interacting protein 3, nuclear gene for mitochondrial product;BCL2/adenovirus E1B 19kDa interacting protein 3;BCL2/adenovirus E1B 19kDa-interacting protein 1, NIP3 | BNIP3;Bnip3 | [RefSeq Summary:] This gene is a member of the BCL2/adenovirus E1B 19 kd-interacting protein (BNIP) family. It interacts with the E1B 19 kDa protein which is responsible for the protection of virally-induced cell death, as well as E1B 19 kDa-like sequences of BCL2, also an apoptotic protector. This gene contains a BH3 domain and a transmembrane domain, which have been associated with pro-apoptotic function. The dimeric mitochondrial protein encoded by this gene is known to induce apoptosis, even in the presence of BCL2. |
| 1422478_a_at | 60525 | acetyl-Coenzyme A synthetase 2 (ADP forming) | ACAS2;Acas2 | [RefSeq Summary:] This gene encodes a cytosolic enzyme that catalyzes the activation of acetate for use in lipid synthesis and energy generation. The protein acts as a monomer and produces acetyl-CoA from acetate in a reaction that requires ATP. Expression of this gene is regulated by sterol regulatory element-binding proteins, transcription factors that activate genes required for the synthesis of cholesterol and unsaturated fatty acids. Two transcript variants encoding different isoforms have been found for this gene. |

| | | | | |
|---|---|---|---|---|
| 1422479_at | 60525 | acetyl-Coenzyme A synthetase 2 (ADP forming) | ACAS2;Acas2 | [RefSeq Summary:] This gene encodes a cytosolic enzyme that catalyzes the activation of acetate for use in lipid synthesis and energy generation. The protein acts as a monomer and produces acetyl-CoA from acetate in a reaction that requires ATP. Expression of this gene is regulated by sterol regulatory element-binding proteins, transcription factors that activate genes required for the synthesis of cholesterol and unsaturated fatty acids. Two transcript variants encoding different isoforms have been found for this gene. |
| 1422491_a_at | 12175 | BCL2/adenovirus E1B 19kDa interacting protein 2;BCL2/adenovirus E1B 19kDa-interacting protein 1, NIP2 | BNIP2;Bnip2 | [RefSeq Summary:] This gene is a member of the BCL2/adenovirus E1B 19 kd-interacting protein (BNIP) family. Though the specific function is unknown, it interacts with the E1B 19 kDa protein which is responsible for the protection of virally-induced cell death, as well as E1B 19 kDa-like sequences of BCL2, also an apoptotic protector. |
| 1422497_at | 69048 | solute carrier family 30 (zinc transporter), member 5 | SLC30A5;Slc30a5 | |

| | | | | |
|---|---|---|---|---|
| 1422526_at | 14081 | fatty acid Coenzyme A ligase, long chain 2;fatty acid Coenzyme A ligase, long chain 3;fatty acid Coenzyme A ligase, long chain 4;fatty acid Coenzyme A ligase, long chain 5;fatty acid-Coenzyme A ligase, long chain 4;fatty-acid-Coenzyme A ligase, long-chain 1;fatty-acid-Coenzyme A ligase, long-chain 2;fatty-acid-Coenzyme A ligase, long-chain 3;fatty-acid-Coenzyme A ligase, long-chain 4;fatty-acid-Coenzyme A ligase, long-chain 5;fatty-acid-Coenzyme A | FACL2;FACL3;FACL4;FACL5;FACL6;Facl2;Facl3;Facl4;Facl5 | [RefSeq Summary:] The protein encoded by this gene is an isozyme of the long-chain fatty-acid-coenzyme A ligase family. Although differing in substrate specificity, subcellular localization, and tissue distribution, all isozymes of this family convert fre |
| 1422533_at | 13121 | cytochrome P450, 51;cytochrome P450, subfamily 51 | Cyp51 | |
| 1422557_s_at | 17748 | metallothionein 1 | Mt1 | |
| 1422731_at | 29806 | LIM domains containing 1 | LIMD1;Limd1 | |
| 1422751_at | 21885 | transducin-like enhancer of split 1 (E(sp1) homolog, Drosophila);transducin-like enhancer of split 1, homolog of Drosophila E(spl) | TLE1;Tle1 | |

| | | | | |
|---|---|---|---|---|
| 1422768_at | 56403 | NS1-associated protein 1 | SYNCRIP;Syncrip | |
| 1422771_at | 17130 | MAD homolog 6 (Drosophila);MAD, mothers against decapentaplegic homolog 6 (Drosophila) | MADH6;Madh6 | |
| 1422788_at | 58207 | embryonic epithelial gene 1;likely ortholog of mouse embryonic epithelial gene 1 | EEG1;Eeg1 | [Proteome Summary:] Low similarity to POV1 |
| 1422901_at | 76055 | meningioma expressed antigen 5 (hyaluronidase) | MGEA5;Mgea5 | [Proteome Summary:] Hyaluronidase; immunogenic antigen in a group of meningioma patients |
| 1422905_s_at | 55990 | flavin containing monooxygenase 2 | FMO2;Fmo2 | [RefSeq Summary:] Metabolic N-oxidation of the diet-derived amino-trimethylamine (TMA) is mediated by flavin-containing monooxygenase and is subject to an inherited FMO3 polymorphism in man resulting in a small subpopulation with reduced TMA N-oxidation capacity resulting in the fish odor syndrome Trimethylaminuria. Three forms of the enzyme, FMO1 found in fetal liver, FMO2 found in adult liver, and FMO3 are encoded by genes clustered in the 1q23-q25 region. Flavin-containing monooxygenases are NADPH-dependent flavoenzymes that catalyzes the oxidation of soft nucleophilic heteroatom centers in drugs, pesticides, and xenobiotics. The major FMO2 allele encodes a truncated polypeptide which is catalytically inactive. |
| 1422906_at | 26357 | ATP-binding cassette protein G2;ATP-binding cassette, sub-family G (WHITE), member 2 | ABCG2;Abcg2 | [RefSeq Summary:] The membrane-associated protein encoded by this gene is included in the superfamily of ATP-binding cassette (ABC) transporters. ABC proteins transport various molecules across extra- and intra-cellular membranes. ABC genes are divided in |
| 1422943_a_at | 15507 | heat shock 27kDa protein 1;heat shock protein 1 | HSPB1;Hspb1 | |

| | | | | |
|---|---|---|---|---|
| 1423121_at | 15925 | Insulin degrading enzyme;insulin degrading enzyme;insulin-degrading enzyme | IDE;Ide | [RefSeq Summary:] This gene may belong to a protease family responsible for intercellular peptide signalling. Though its role in the cellular processing of insulin has not yet been defined, insulin-degrading enzyme is thought to be involved in the termination of the insulin response. |
| 1423223_a_at | 11758 | arylacetamide deacetylase (esterase);eosinophil peroxidase;lipase, hormone sensitive;lipase, hormone sensitive;lipase, hormone-sensitive;myeloperoxidase;peroxiredoxin 6 | AADAC;EPX;Epx;LIPE;Lipe;MPO;Mpo;PRDX6;Prdx6 | [RefSeq Summary:] Microsomal arylacetamide deacetylase competes against the activity of cytosolic arylamine N-acetyltransferase, which catalyzes one of the initial biotransformation pathways for arylamine and heterocyclic amine carcinogens ;[RefSeq Summar |
| 1423256_a_at | 66290 | ATPase, H+ transporting, V1 subunit G isoform 1;ATPase, H+ transporting, V1 subunit G isoform 2;ATPase, H+ transporting, lysosomal 13kDa, V1 subunit G isoform 1;ATPase, H+ transporting, lysosomal 13kDa, V1 subunit G isoform 2;ATPase, H+ transporting, lysosomal 13kDa, V1 subunit G isoform 3 | ATP6V1G1;ATP6V1G2;ATP6V1G3;Atp6v1g1;Atp6v1g2 | [RefSeq Summary:] This gene encodes a component of vacuolar ATPase (V-ATPase), a multisubunit enzyme that mediates acidification of eukaryotic intracellular organelles. V-ATPase dependent organelle acidification is necessary for such intracellular process |

| | | | | |
|---|---|---|---|---|
| 1423269_a_at | 83814 | E3 ubiquitin ligase SMURF2;Nedd-4-like ubiquitin-protein ligase;hect (homologous to the E6-AP (UBE3A) carboxyl terminus) domain and RCC1 (CHC1)-like domain (RLD) 1;hect domain and RLD 2;itchy homolog E3 ubiquitin protein ligase (mouse);neural precursor cell expressed, developmentally down-regulated 4-like;neural precursor cell expressed, developmentally down-regulated gene 4-like;neural precursor cell | DD5;HERC1;HERC2;ITCH;NEDD4L;Nedd4;Nedd4l;SMURF2;WWP2 | [RefSeq Summary:] Atrophin-1 contains a polyglutamine repeat, expansion of which is responsible for dentatorubral and pallidoluysian atrophy. The protein encoded by this gene interacts with atrophin-1. This encoded protein is a closely related member of t |
| 1423308_at | 22134 | trans-golgi network protein | TGOLN1;Tgoln1 | |
| 1423324_at | 18949 | pinin;pinin, desmosome associated protein | PNN;Pnn | |
| 1423383_a_at | 230598 | N-arginine dibasic convertase 1;nardilysin (N-arginine dibasic convertase);nardilysin, N-arginine dibasic convertase, NRD convertase 1 | NRD1;Nrd1 | |

| | | | | |
|---|---|---|---|---|
| 1423437_at | 14859 | Glutathione S-transferase 1 (theta);Glutathione-S-transferase, mu type 2 (Yb2);glutathione S-transferase A1;glutathione S-transferase A2;glutathione S-transferase A3;glutathione S-transferase A4;glutathione S-transferase M1;glutathione S-transferase M2 (m | GSTA1;GSTA2;GSTA3;GSTA4;GSTM1;GSTM2;GSTM3;GSTM4;GSTM5;GSTO1;GSTP1;GSTT1;GSTT2;GSTZ1;GstYb4;Gsta1;Gsta2;Gsta3;Gsta4;Gstm1;Gstm2;Gstm3;Gstm4;Gstm5;Gstm6;Gsto1;Gstp1;Gstp2;Gstt1;Gstt2;Gstz1;LOC51064;MGST1;MGST2;MGST3;Mgst3 | [RefSeq Summary:] Cytosolic and membrane-bound forms of glutathione S-transferase are encoded by two distinct supergene families. At present, eight distinct classes of the soluble cytoplasmic mammalian glutathione S-transferases have been identified: alph |
| 1423439_at | 18534 | phosphoenolpyruvate carboxykinase 1 (soluble);phosphoenolpyruvate carboxykinase 1, cytosolic;phosphoenolpyruvate carboxykinase 2 (mitochondrial) | PCK1;PCK2;Pck1 | [RefSeq Summary:] Phosphoenolpyruvate carboxykinase (PCK; EC 4.1.1.32) is a key enzyme in gluconeogenesis. The activity is about equally distributed between cytosol and mitochondria in human liver. In contrast, PCK is essentially a cytosolic enzyme in rat liver. See also PCK1 (MIM 261680), the human cytosolic PCK enzyme.[supplied by OMIM] ;[RefSeq Summary:] This gene is a main control point for the regulation of gluconeogenesis. The cytosolic enzyme encoded by this gene, along with GTP, catalyzes the formation of phosphoenolpyruvate from oxaloacetate, with the release of carbon dioxide and GDP. The expression of this gene can be regulated by insulin, glucocorticoids, glucagon, cAMP, and diet. A mitochondrial isozyme of the encoded protein also has been characterized. |
| 1423447_at | 270166 | ClpX caseinolytic protease X homolog (E. coli);caseinolytic protease X (E.coli) | CLPX;Clpx | |
| 1423566_a_at | 15505 | heat shock protein 105 | Hsp105 | |

| | | | | |
|---|---|---|---|---|
| 1423652_at | 69046 | RIKEN cDNA 1810010A06 gene;hypothetical protein MGC4276 similar to CG8198 | HBLD2;Hbld2 | |
| 1423680_at | 76267 | RIKEN cDNA 0710001O03 gene | 0710001O03Rik | |
| 1423717_at | 56248 | adenylate kinase 3;adenylate kinase 3 alpha-like;adenylate kinase 3 like 1 | AK3L1;Ak3;Ak3l | |
| 1423723_s_at | 230908 | TAR DNA binding protein | TARDBP;Tardbp | [RefSeq Summary:] HIV-1, the causative agent of acquired immunodeficiency syndrome (AIDS), contains an RNA genome that produces a chromosomally integrated DNA during the replicative cycle. Activation of HIV-1 gene expression by the transactivator Tat is dependent on an RNA regulatory element (TAR) located downstream of the transcription initiation site. The protein encoded by this gene is a transcriptional repressor that binds to chromosomally integrated TAR DNA and represses HIV-1 transcription. In addition, this protein regulates alternate splicing of the CFTR gene. A similar pseudogene is present on chromosome 20. |
| 1423797_at | 78894 | acetoacetyl-CoA synthetase | AACS;Aacs | |
| 1423799_at | 20918 | suppressor of initiator codon mutations, related sequence 1 (S. cerevisiae) | Sui1-rs1 | |
| 1423819_s_at | 54208 | ADP-ribosylation-like factor 6 interacting protein | Arl6ip1 | |
| 1423883_at | | | | |
| 1423974_at | 101706 | expressed sequence AL022610 | AL022610 | |

| | | | | |
|---|---|---|---|---|
| 1424005_at | 78521 | RIKEN cDNA B230219D22 gene | B230219D22Rik | |
| 1424101_at | 15388 | heterogeneous nuclear ribonucleoprotein L | HNRPL;Hnrpl | [RefSeq Summary:] Heterogeneous nuclear RNAs (hnRNAs) which include mRNA precursors and mature mRNAs are associated with specific proteins to form heterogenous ribonucleoprotein (hnRNP) complexes. Heterogeneous nuclear ribonucleoprotein L is among the pro |
| 1424126_at | 11655 | aminolevulinate, delta-, synthase 1;aminolevulinic acid synthase 1 | ALAS1;Alas1 | |
| 1424147_at | 217737 | AHA1, activator of heat shock 90kDa protein ATPase homolog 1 (yeast) | AHSA1;Ahsa1 | |
| 1424175_at | 21685 | thyrotroph embryonic factor;thyrotrophic embryonic factor | TEF;Tef | [RefSeq Summary:] Thyrotroph embryonic factor (TEF), a transcription factor, is a member of the PAR (proline and acidic amino acid-rich) subfamily of basic region/leucine zipper (bZIP) transcription factors. It is expressed in a broad range of cells and |
| 1424274_at | 56041 | vesicle docking protein;vesicle docking protein p115;vesicle docking protein, 115 kDa | VDP;Vdp | [RefSeq Summary:] The protein encoded by this gene is a peripheral membrane protein which recycles between the cytosol and the Golgi apparatus during interphase. It is regulated by phosphorylation: dephosphorylated protein associates with the Golgi membrane and dissociates from the membrane upon phosphorylation. Ras-associated protein 1 recruits this protein to coat protein complex II (COPII) vesicles during budding from the endoplasmic reticulum, where it interacts with a set of COPII vesicle-associated SNAREs to form a cis-SNARE complex that promotes targeting to the Golgi apparatus. Transport from the ER to the cis/medial Golgi compartments requires the action of this gene product, GM130 and giantin in a sequential manner. |
| 1424444_a_at | 72244 | RIKEN cDNA 1600014C10 gene | 1600014C10Rik | |

| | | | | |
|---|---|---|---|---|
| 1424574_at | 73130 | RIKEN cDNA 3110020O18 gene | 3110020O18Rik | |
| 1424638_at | 12575 | cyclin-dependent kinase inhibitor 1A;cyclin-dependent kinase inhibitor 1A (P21);cyclin-dependent kinase inhibitor 1A (p21, Cip1) | CDKN1A;Cdkn1a | [RefSeq Summary:] This gene encodes a potent cyclin-dependent kinase inhibitor. The encoded protein binds to and inhibits the activity of cyclin-CDK2 or -CDK4 complexes, and thus functions as a regulator of cell cycle progression at G1. The expression of |
| 1424669_at | 68520 | RIKEN cDNA 1110013H04 gene;zinc finger, FYVE domain containing 21 | ZFYVE21;Zfyve21 | |
| 1424671_at | 72287 | RIKEN cDNA 1810013P09 gene;pleckstrin homology domain containing, family F (with FYVE domain) member 1 | PLEKHF1;Plekhf1 | |
| 1424712_at | 226747 | ELYS transcription factor-like protein TMBS62;embryonic large molecule derived from yolk sac | ELYS;Elys | |
| 1424769_s_at | 109624 | Caldesmon 1;caldesmon 1 | CALD1;Cald1 | [RefSeq Summary:] This gene encodes a calmodulin- and actin-binding protein, which plays a vital role in the regulation of smooth muscle and nonmuscle contraction. This gene consists of at least 16 exons, and its alternative splicing generates several transcript variants varying in 5' UTR and coding region. These variants encode at least 5 different isoforms. The high molecular weight isoform (isoform 1) is predominantly expressed in smooth muscles, whereas the low molecular weight isoforms (isoforms 2-5) are widely distributed in nonmuscle tissues and cells. |

| | | | | |
|---|---|---|---|---|
| 1424770_at | 109624 | Caldesmon 1;caldesmon 1 | CALD1;Cald1 | [RefSeq Summary:] This gene encodes a calmodulin- and actin-binding protein, which plays a vital role in the regulation of smooth muscle and nonmuscle contraction. This gene consists of at least 16 exons, and its alternative splicing generates several transcript variants varying in 5' UTR and coding region. These variants encode at least 5 different isoforms. The high molecular weight isoform (isoform 1) is predominantly expressed in smooth muscles, whereas the low molecular weight isoforms (isoforms 2-5) are widely distributed in nonmuscle tissues and cells. |
| 1424786_s_at | 54636 | DNA segment, Chr X, Immunex 38, expressed | DXImx38e | |
| 1424915_s_at | 217732 | RIKEN cDNA 2310044G17 gene | 2310044G17Rik | |
| 1424980_s_at | 226548 | anterior pharynx defective 1A homolog (C. elegans) | 6530402N02Rik | |
| 1425020_at | 217379 | RIKEN cDNA 6330407P03 gene;hypothetical protein LOC165324 | UBXD4;Ubxd4 | |
| 1425021_a_at | 18633 | peroxisomal biogenesis factor 16;peroxisome biogenesis factor 16 | PEX16;Pex16 | [RefSeq Summary:] The protein encoded by this gene is an integral peroxisomal membrane protein. An inactivating nonsense mutation localized to this gene was observed in a patient with Zellweger syndrome of the complementation group CGD/CG9. Expression of this gene product morphologically and biochemically restores the formation of new peroxisomes, suggesting a role in peroxisome organization and biogenesis. Alternative splicing has been observed for this gene and two variants have been described. |
| 1425099_a_at | 11865 | aryl hydrocarbon receptor nuclear translocator-like;transcription factor BMAL2 | ARNTL;ARNTL2;Arntl | [Proteome Summary:] BMAL2; functions as a transcription factor |

| | | | | |
|---|---|---|---|---|
| 1425262_at | 12611 | CCAAT/enhancer binding protein (C/EBP), gamma;CCAAT/ enhancer binding protein ,gamma | CEBPG;Cebpg | [RefSeq Summary:] The C/EBP family of transcription factors regulates viral and cellular CCAAT/enhancer element-mediated transcription. C/EBP proteins contain the bZIP region, which is characterized by two motifs in the C-terminal half of the protein: a basic region involved in DNA binding and a leucine zipper motif involved in dimerization. The C/EBP family consist of several related proteins, C/EBP alpha, C/EBP beta, C/EBP gamma, and C/EBP delta, that form homodimers and that form heterodimers with each other. CCAAT/enhancer binding protein gamma may cooperate with Fos to bind PRE-I enhancer elements. |
| 1425281_a_at | 14605 | glucocorticoid-induced leucine zipper | Dsip1 | |
| 1425296_a_at | 50780 | regulator of G-protein signaling 3;regulator of G-protein signalling 3 | RGS3;Rgs3 | [RefSeq Summary:] This gene encodes a member of the regulator of G-protein signaling (RGS) family. This protein is a GTP-ase activating protein which inhibits G-protein mediated signal transduction. The protein is largely cytosolic, but G-protein activation leads to translocation of this protein to the plasma membrane. A nuclear form of this protein has also been described, but its sequence has not been identified. Multiple alternatively spliced transcript variants have been described for this gene but the full length nature of some transcripts is not yet known. |
| 1425326_at | 104112 | ATP citrate lyase | ACLY;Acly | [RefSeq Summary:] ATP citrate lyase is the primary enzyme responsible for the synthesis of cytosolic acetyl-CoA in many tissues. The enzyme is a tetramer (relative molecular weight approximately 440,000) of apparently identical subunits. It catalyzes the formation of acetyl-CoA and oxaloacetate from citrate and CoA with a concomitant hydrolysis of ATP to ADP and phosphate. The product, acetyl-CoA, serves several important biosynthetic pathways, including lipogenesis and cholesterogenesis. In nervous tissue, ATP citrate-lyase may be involved in the biosynthesis of acetylcholine. Two transcript variants encoding distinct isoforms have been identified for this gene. |

| | | | | |
|---|---|---|---|---|
| 1425343_at | 72748 | RIKEN cDNA 2810435D12 gene | 2810435D12Rik | |
| 1425568_a_at | 67878 | RIKEN cDNA 1600019D15 gene | 1600019D15Rik | |
| 1425631_at | 53412 | protein phosphatase 1, regulatory (inhibitor) subunit 3C | PPP1R3C;Ppp1r3c | [RefSeq Summary:] Protein phosphatase-1 (PP1; see MIM 176875) participates in the regulation of a wide variety of cellular functions by reversible protein phosphorylation. The ability of PP1 to regulate diverse functions resides in its capacity to interact with a variety of regulatory subunits that may target PP1 to specific subcellular locations, modulate its substrate specificity, and allow its activity to be responsive to extracellular signals. Several targeting subunits of PP1 have been identified, including PPP1R5, the glycogen-binding subunits PPP1R3 (MIM 600917) and PPP1R4, and the nuclear inhibitor of PP1 (PPP1R8; MIM 602636).[supplied by OMIM] |
| 1425678_a_at | 20623 | SNF related kinase;SNF-1 related kinase | SNRK;Snrk | |
| 1425701_a_at | 50780 | regulator of G-protein signaling 3;regulator of G-protein signalling 3 | RGS3;Rgs3 | [RefSeq Summary:] This gene encodes a member of the regulator of G-protein signaling (RGS) family. This protein is a GTPase activating protein which inhibits G-protein mediated signal transduction. The protein is largely cytosolic, but G-protein activation leads to translocation of this protein to the plasma membrane. A nuclear form of this protein has also been described, but its sequence has not been identified. Multiple alternatively spliced transcript variants have been described for this gene but the full length nature of some transcripts is not yet known. |
| 1425742_a_at | 21807 | Transforming growth factor beta stimulated clone 22;transforming growth factor beta 1 induced transcript 4 | Tgfb1i4 | |

| | | | | |
|---|---|---|---|---|
| 1425792_a_at | 19885 | RAR-related orphan receptor C;RAR-related orphan receptor gamma | RORC;Rorc | [RefSeq Summary:] The protein encoded by this gene is a DNA-binding transcription factor and is a member of the NR1 subfamily of nuclear hormone receptors. The specific functions of this protein are not known; however, studies of a similar gene in mice have shown that this gene may be essential for lymphoid organogenesis and may play an important regulatory role in thymopoiesis. In addition, studies in mice suggest that the protein encoded by this gene may inhibit the expression of Fas ligand and IL2. |
| 1425829_a_at | 117167 | Tnfa-induced adipose-related protein | AI481214 | |
| 1425834_a_at | 14732 | glycerol 3-phosphate acyltransferase, mitochondrial;glycerol-3-phosphate acyltransferase, mitochondrial | Gpam;KIAA1560 | |
| 1425964_x_at | 15507 | heat shock 27kDa protein 1;heat shock protein 1 | HSPB1;Hspb1 | |
| 1425966_x_at | 22190 | ubiquitin C | UBC;Ubc | [Proteome  Summary:] Ubiquitin C; polyubiquitin protein precursor that marks cellular proteins for degradation |
| 1425993_a_at | 15505 | heat shock protein 105 | Hsp105 | |
| 1426037_a_at | 19734 | Regulator of G-protein signaling 16;regulator of G-protein signaling 16;regulator of G-protein signalling 16 | RGS16;Rgs16 | [RefSeq Summary:] The protein encoded by this gene belongs to the 'regulator of G protein signaling' family. It inhibits signal transduction by increasing the GTPase activity of G protein alpha subunits. It also may play a role in regulating the kinetics of signaling in the phototransduction cascade. |
| 1426084_a_at | 208263 | hypothetical protein MGC6357 | MGC6357 | |
| 1426124_a_at | 12747 | CDC-like kinase | Clk | |

| | | | | |
|---|---|---|---|---|
| 1426245_s_at | 212307 | microtubule-associated protein, RP/EB family, member 2 | MAPRE2;Mapre2 | [RefSeq Summary:] The protein encoded by this gene shares significant homology to the adenomatous polyposis coli (APC) protein-binding EB1 gene family. The function of this protein is unknown; however, its homology suggests involvement in tumorigenesis of colorectal cancers and proliferative control of normal cells. This gene may belong to the intermediate/early gene family, involved in the signal transduction cascade downstream of the TCR. |
| 1426256_at | 21854 | translocase of inner mitochondrial membrane 17 homolog A (yeast);translocator of inner mitochondrial membrane 17a (yeast);translocator of inner mitochondrial membrane a | TIMM17A;Timm17a | |
| 1426342_at | 68292 | source of immunodominant MHC-associated peptides | 1300006C19Rik | |
| 1426356_at | 76178 | RIKEN cDNA 6330578E17 gene | 6330578E17Rik | |
| 1426432_a_at | 54403 | solute carrier family 4 (anion exchanger), member 4;solute carrier family 4, member 4;solute carrier family 4, sodium bicarbonate cotransporter, member 4 | SLC4A4;Slc4a4 | [Proteome Summary:] Sodium bicarbonate cotransporter 4 |
| 1426457_at | 83997 | sarcolemma associated protein | SLMAP;Slmap | [Proteome Summary:] Membrane protein with predicted coiled coil structures |
| 1426459_s_at | 106064 | expressed sequence AW549877 | AW549877 | |

| | | | | |
|---|---|---|---|---|
| 1426464_at | 217166 | nuclear receptor subfamily 1, group D, member 1 | NR1D1;Nr1d1 | |
| 1426489_s_at | 67118 | RIKEN cDNA 3010001A07 gene;bifunctional apoptosis regulator | BFAR;Bfar | |
| 1426490_at | 67118 | RIKEN cDNA 3010001A07 gene;bifunctional apoptosis regulator | BFAR;Bfar | |
| 1426584_a_at | 20322 | sorbitol dehydrogenase;sorbitol dehydrogenase 1 | SORD;Sdh1;Sord | |
| 1426624_a_at | 66090 | RIKEN cDNA 0610043B10 gene | 0610043B10Rik | |
| 1426645_at | 15519 | heat shock 90kDa protein 1, alpha;heat shock protein 1, alpha | HSPCA;Hspca | |
| 1426722_at | 67760 | solute carrier family 38, member 2 | SLC38A2;Slc38a2 | |
| 1426798_a_at | 108954 | RIKEN cDNA 1810033K10 gene;protein phosphatase 1, regulatory (inhibitor) subunit 15B | PPP1R15B;Ppp1r15b | |
| 1426894_s_at | 98952 | RIKEN cDNA C230093N12 gene | C230093N12Rik | |
| 1426915_at | 69635 | death associated protein kinase 1;death-associated protein kinase 1 | DAPK1;Dapk1 | [RefSeq Summary:] Death-associated protein kinase 1 is a positive mediator of gamma-interferon induced programmed cell death. DAPK1 encodes a structurally unique 160-kD calmodulin dependent serine-threonine kinase that carries 8 ankyrin repeats and 2 putative P-loop consensus sites. It is a tumor suppressor candidate. |

| | | | | |
|---|---|---|---|---|
| 1426955_at | 12822 | collagen, type XVIII, alpha 1;procollagen, type XVIII, alpha 1 | COL18A1;Col18a1 | [RefSeq Summary:] This gene encodes the alpha chain of type XVIII collagen. This collagen is one of the multiplexins, extracellular matrix proteins that contain multiple triple-helix domains (collagenous domains) interrupted by non-collagenous domains. The proteolytically produced C-terminal fragment of type XVIII collagen is endostatin, a potent antiangiogenic protein. Mutations in this gene are associated with Knobloch syndrome. The main features of this syndrome involve retinal abnormalities so type XVIII collagen may play an important role in retinal structure and in neural tube closure. Three transcripts have been identified for this gene. |
| 1427040_at | 16543 | kidney cell line derived transcript 1 | Kdt1 | |
| 1427087_at | 192196 | LUC7-like 2 (S. cerevisiae) | LUC7L2;Luc7l2 | |
| 1427202_at | 320204 | RIKEN cDNA 4833442J19 gene | 4833442J19Rik | |
| 1427250_at | 11938 | ATPase, Ca++ transporting, cardiac muscle, slow twitch 2 | ATP2A2;Atp2a2 | [RefSeq Summary:] This gene encodes one of the SERCA Ca(2+)-ATPases, which are intracellular pumps located in the sarcoplasmic or endoplasmic reticula of muscle cells. This enzyme catalyzes the hydrolysis of ATP coupled with the translocation of calcium from the cytosol to the sarcoplasmic reticulum lumen, and is involved in regulation of the contraction/relaxation cycle. Mutations in this gene cause Darier-White disease, also known as keratosis follicularis, an autosomal dominant skin disorder characterized by loss of adhesion between epidermal cells and abnormal keratinization. Alternative splicing results in two transcript variants encoding different isoforms. |
| 1427347_s_at | 22151 | tubulin, beta 2;tubulin, beta, 2 | TUBB2;Tubb2 | |

| | | | | |
|---|---|---|---|---|
| 1427358_a_at | 69635 | death associated protein kinase 1;death-associated protein kinase 1 | DAPK1;Dapk1 | [RefSeq Summary:] Death-associated protein kinase 1 is a positive mediator of gamma-interferon induced programmed cell death. DAPK1 encodes a structurally unique 160-kD calmodulin dependent serine-threonine kinase that carries 8 ankyrin repeats and 2 putative P-loop consensus sites. It is a tumor suppressor candidate. |
| 1427413_a_at | 13046 | CUG triplet repeat, RNA binding protein 1 | CUGBP1;Cugbp1 | [RefSeq Summary:] The CUGBP1 gene encodes a member of the CELF family of RNA processing factors that regulates pre-mRNA splicing.[supplied by OMIM] |
| 1427422_at | | | | |
| 1427559_a_at | 11909 | RATF2;activating transcription factor 2 | ATF2;Atf2 | [RefSeq Summary:] This gene encodes a transcription factor that is a member of the leucine zipper family of DNA binding proteins. This protein binds to the cAMP-responsive element (CRE), an octameric palindrome. The protein forms a homodimer or heterodimer with c-Jun and stimulates CRE-dependent transcription. The protein is also a histone acetyltransferase (HAT) that specifically acetylates histones H2B and H4 in vitro; thus it may represent a class of sequence-specific factors that activate transcription by direct effects on chromatin components. Additional transcript variants have been identified but their biological validity has not been determined. |
| 1427653_at | 21473 | T-cell receptor alpha chain | Tcra | |
| 1427893_a_at | 68603 | RIKEN cDNA 1110011E12 gene;phosphomevalonate kinase | PMVK;Pmvk | |
| 1427929_a_at | 216134 | pyridoxal (pyridoxine, vitamin B6) kinase;pyridoxal kinase | PDXK;Pdxk | |
| 1427963_s_at | 103142 | retinol dehydrogenase 9 | Rdh9 | |
| 1428070_at | 74126 | HRD1 protein | 1200010C09Rik | |
| 1428071_at | 68778 | RIKEN cDNA 1110038D17 gene | 1110038D17Rik | |

| | | | | |
|---|---|---|---|---|
| 1428082_at | 433256 | | | |
| 1428106_at | 74148 | RIKEN cDNA 1300001I01 gene | 1300001I01Rik | |
| 1428289_at | 16601 | basic transcription element binding protein 1 | BTEB1;Bteb1 | [RefSeq Summary:] Basic transcription element binding protein 1 (BTEB1) is a transcription factor that binds to GC box elements located in the promoter. Binding of BTEB1 to a single GC box inhibits mRNA expression while BTEB1 binding to tandemly repeated GC box elements activates transcription. |
| 1428306_at | 74747 | HIF-1 responsive RTP801 | 5830413E08Rik | |
| 1428357_at | 72148 | RIKEN cDNA 2610019F03 gene | 2610019F03Rik | |
| 1428369_s_at | 71435 | RIKEN cDNA 5530401C11 gene | 5530401C11Rik | |
| 1428690_at | 71767 | RIKEN cDNA 1300019N10 gene | 1300019N10Rik | |
| 1428772_at | 73192 | exportin, tRNA (nuclear export receptor for tRNAs) | XPOT;Xpot | [RefSeq Summary:] This gene encodes a protein belonging to the RAN-GTPase exportin family that mediates export of tRNA from the nucleus to the cytoplasm. Translocation of tRNA to the cytoplasm occurs once exportin has bound both tRNA and GTP-bound RAN. |
| 1428929_s_at | 67582 | RIKEN cDNA 4930433D19 gene;hypothetical protein LOC115286 | SLC25A26;Slc25a26 | |
| 1428942_at | 17750 | metallothionein 2 | Mt2 | |
| 1429002_at | 66354 | SKI interacting protein | Skiip | |
| 1429568_x_at | 67921 | RIKEN cDNA 2510010F15 gene | 2510010F15Rik | |
| 1429758_at | 74211 | RIKEN cDNA 1700017B05 gene | 1700017B05Rik | |

| | | | | |
|---|---|---|---|---|
| 1430019_a_at | 15382 | heterogeneous nuclear ribonucleoprotein A1 | HNRPA1;Hnrpa1 | [RefSeq Summary:] This gene belongs to the A/B subfamily of ubiquitously expressed heterogeneous nuclear ribonucleoproteins (hnRNPs). The hnRNPs are RNA binding proteins and they complex with heterogeneous nuclear RNA (hnRNA). These proteins are associate |
| 1430128_a_at | 70335 | deleted in polyposis 1-like 1;polyposis locus protein 1-like 1 | DP1L1;Dp1l1 | |
| 1431037_a_at | 15568 | ELAV (embryonic lethal, abnormal vision, Drosophila)-like 1 (Hu antigen R) | ELAVL1;Elavl1 | [RefSeq Summary:] The protein encoded by this gene is a member of the ELAVL protein family. This encoded protein contains 3 RNA-binding domains and binds cis-acting AU-rich elements. It destabilizes mRNAs and thereby regulates gene expression. |
| 1432143_a_at | 73389 | HMG-box containing protein 1;RIKEN cDNA 1700058O05 gene | HBP1;Hbp1 | |
| 1432195_s_at | 56036 | cyclin L2 | CCNL2;Ccnl2 | |
| 1432646_a_at | 14705 | Bernardinelli-Seip congenital lipodystrophy 2 (seipin);Bernardinelli-Seip congenital lipodystrophy 2 homolog (human) | BSCL2;Bscl2 | |

| | | | | |
|---|---|---|---|---|
| 1433443_a_at | 208715 | 3-hydroxy-3-methylglutaryl-Coenzyme A synthase 1;3-hydroxy-3-methylglutaryl-Coenzyme A synthase 1 (soluble);3-hydroxy-3-methylglutaryl-Coenzyme A synthase 2;3-hydroxy-3-methylglutaryl-Coenzyme A synthase 2 (mitochondrial) | HMGCS1;HMGCS2;Hmgcs1;Hmgcs2 | |
| 1433444_at | 208715 | 3-hydroxy-3-methylglutaryl-Coenzyme A synthase 1;3-hydroxy-3-methylglutaryl-Coenzyme A synthase 1 (soluble);3-hydroxy-3-methylglutaryl-Coenzyme A synthase 2;3-hydroxy-3-methylglutaryl-Coenzyme A synthase 2 (mitochondrial) | HMGCS1;HMGCS2;Hmgcs1;Hmgcs2 | |

| | | | | |
|---|---|---|---|---|
| 1433445_x_at | 208715 | 3-hydroxy-3-methylglutaryl-Coenzyme A synthase 1;3-hydroxy-3-methylglutaryl-Coenzyme A synthase 1 (soluble);3-hydroxy-3-methylglutaryl-Coenzyme A synthase 2;3-hydroxy-3-methylglutaryl-Coenzyme A synthase 2 (mitochondrial) | HMGCS1;HMGCS2;Hmgcs1;Hmgcs2 | |
| 1433446_at | 208715 | 3-hydroxy-3-methylglutaryl-Coenzyme A synthase 1;3-hydroxy-3-methylglutaryl-Coenzyme A synthase 1 (soluble);3-hydroxy-3-methylglutaryl-Coenzyme A synthase 2;3-hydroxy-3-methylglutaryl-Coenzyme A synthase 2 (mitochondrial) | HMGCS1;HMGCS2;Hmgcs1;Hmgcs2 | |
| 1433448_at | 229517 | RIKEN cDNA B430110G05 gene | B430110G05Rik | |
| 1433491_at | 13822 | erythrocyte protein band 4.1-like 2 | Epb4.1l2 | |
| 1433514_at | 75320 | RIKEN cDNA 4930555L11 gene | 4930555L11Rik | |
| 1433545_s_at | 102632 | RIKEN cDNA 5730439E10 gene | 5730439E10Rik | |
| 1433582_at | 68861 | RIKEN cDNA 1190002N15 gene | 1190002N15Rik | |

| | | | | |
|---|---|---|---|---|
| 1433645_at | 70174 | RIKEN cDNA 2210409B22 gene | 2210409B22Rik | |
| 1433691_at | 53412 | protein phosphatase 1, regulatory (inhibitor) subunit 3C | PPP1R3C;Ppp1r3c | [RefSeq Summary:] Protein phosphatase-1 (PP1; see MIM 176875) participates in the regulation of a wide variety of cellular functions by reversible protein phosphorylation. The ability of PP1 to regulate diverse functions resides in its capacity to interact with a variety of regulatory subunits that may target PP1 to specific subcellular locations, modulate its substrate specificity, and allow its activity to be responsive to extracellular signals. Several targeting subunits of PP1 have been identified, including PPP1R5, the glycogen-binding subunits PPP1R3 (MIM 600917) and PPP1R4, and the nuclear inhibitor of PP1 (PPP1R8; MIM 602636).[supplied by OMIM] |
| 1433733_a_at | 12952 | cryptochrome 1 (photolyase-like);cryptochrome 2 (photolyase-like);similar to cryptochrome 1 (photolyase-like) | CRY1;Cry1;Cry2 | |
| 1433816_at | 14123 | fibrosin 1 | FBS1;Fbs1 | |
| 1433951_at | 75423 | RIKEN cDNA 2810410P22 gene | 2810410P22Rik | |
| 1433995_s_at | | | | |

| | | | | |
|---|---|---|---|---|
| 1434005_at | 56878 | RNA binding motif, single stranded interacting protein 1 | RBMS1;Rbms1 | [RefSeq Summary:] RBMS1 gene product is a member of a small family of proteins which bind single stranded DNA/RNA. These proteins are characterized by the presence of two sets of ribonucleoprotein consensus sequence (RNP-CS) that contain conserved motifs, RNP1 and RNP2, originally described in RNA binding proteins, and required for DNA binding. The RBMS proteins have been implicated in such diverse functions as DNA replication, gene transcription, cell cycle progression and apoptosis. Multiple transcript variants of RBMS1 resulting from alternative splicing and encoding different isoforms, have been described. Several of these were isolated by virtue of their binding to either strand of an upstream element of c-myc (MSSPs), or by phenotypic complementation of cdc2 and cdc13 mutants of yeast (scr2), or as a potential human repressor of HIV-1 and ILR-2 alpha promoter transcription (YC1). |
| 1434087_at | 17769 | 5,10-methylenetetrahydrofolate reductase;5,10-methylenetetrahydrofolate reductase (NADPH);methylenetetrahydrofolate reductase | MTHFR;Mthfr | [RefSeq Summary:] Methylenetetrahydrofolate reductase (EC 1.5.1.20) catalyzes the conversion of 5,10-methylenetetrahydrofolate to 5-methyltetrahydrofolate, a cosubstrate for homocysteine remethylation to methionine.[supplied by OMIM] |
| 1434181_at | 218952 | pleckstrin homology domain containing, family C (with FERM domain) member 1 | PLEKHC1;Plekhc1 | |

| | | | | |
|---|---|---|---|---|
| 1434486_x_at | 216558 | UDP-glucose pyrophosphorylase 2 | UGP2;Ugp2 | [RefSeq Summary:] The enzyme encoded by this gene is an important intermediary in mammalian carbohydrate interconversions. It transfers a glucose moiety from glucose-1-phosphate to MgUTP and forms UDP-glucose and MgPPi. In liver and muscle tissue, UDP-glucose is a direct precursor of glycogen; in lactating mammary gland it is converted to UDP-galactose which is then converted to lactose. The eukaryotic enzyme has no significant sequence similarity to the prokaryotic enzyme. Transcript variants have been described but are not supported by experimental evidence. |
| 1434510_at | 23972 | 3'-phosphoadenosine 5'-phosphosulfate synthase 1;3'-phosphoadenosine 5'-phosphosulfate synthase 2 | PAPSS1;PAPSS2;Papss1;Papss2 | [RefSeq Summary:] Three-prime-phosphoadenosine 5-prime-phosphosulfate (PAPS) is the sulfate donor cosubstrate for all sulfotransferase (SULT) enzymes (Xu et al., 2000 [PubMed 10679223]). SULTs catalyze the sulfate conjugation of many endogenous and exogenous compounds, including drugs and other xenobiotics. In humans, PAPS is synthesized from adenosine 5-prime triphosphate (ATP) and inorganic sulfate by 2 isoforms, PAPSS1 and PAPSS2 (MIM 603005).[supplied by OMIM] |
| 1434545_x_at | 66162 | RIKEN cDNA 1110025L05 gene | 1110025L05Rik | |
| 1434735_at | | | | |
| 1434736_at | 217082 | hepatic leukemia factor | HLF;Hlf | |
| 1435160_at | 268390 | RIKEN cDNA 1110064P04 gene | 1110064P04Rik | |
| 1435222_at | 108655 | forkhead box P1 | FOXP1;Foxp1 | |
| 1435250_at | 12448 | cyclin E 2;cyclin E2 | CCNE2;Ccne2 | [RefSeq Summary:] The protein encoded by this gene belongs to the highly conserved cyclin family, whose members are characterized by a dramatic periodicity in protein abundance through the cell cycle. Cyclins function as regulators of CDK kinases. Differe |
| 1435444_at | 226641 | RIKEN cDNA 9130025P16 gene | 9130025P16Rik | |

| | | | | |
|---|---|---|---|---|
| 1435626_a_at | 64209 | homocysteine-inducible, endoplasmic reticulum stress-inducible, ubiquitin-like domain member 1 | HERPUD1;Herpud1 | |
| 1435803_a_at | 26987 | eukaryotic translation initiation factor 4E;eukaryotic translation initiation factor 4E like 3;eukaryotic translation initiation factor 4E-like 3 | EIF4E;EIF4EL3;Eif4e;Eif4el3 | |
| 1435859_x_at | 19181 | Proteasome (prosome, macropain) 26S subunit, ATPase;proteasome (prosome, macropain) 26S subunit, ATPase 2;proteasome (prosome, macropain) 26S subunit, ATPase, 2 | PSMC2;Psmc2 | [RefSeq Summary:] The 26S proteasome is a multicatalytic proteinase complex with a highly ordered structure composed of 2 complexes, a 20S core and a 19S regulator. The 20S core is composed of 4 rings of 28 non-identical subunits; 2 rings are composed of |
| 1435872_at | 18712 | pim-1 oncogene;proviral integration site 1 | PIM1;Pim1 | [RefSeq Summary:] The protooncogene PIM1 encodes a protein kinase upregulated in prostate cancer.[supplied by OMIM] |
| 1436058_at | 58185 | viral hemorrhagic septicemia virus(VHSV) induced gene 1 | 2510004L01Rik | |
| 1436228_at | 360216 | TRAF-binding protein | Trabid | |

| | | | | |
|---|---|---|---|---|
| 1436297_a_at | 66168 | NMDA receptor glutamate-binding chain;RIKEN cDNA 1110025J15 gene;glutamate receptor, ionotropic, N-methyl D-asparate-associated protein 1 (glutamate binding) | GRINA;Grina | [Proteome Summary:] Glutamate-binding subunit of an N-methyl-D-aspartate (NMDA) receptor; a form of ionotropic glutamate receptor |
| 1436316_at | 109249 | RIKEN cDNA 9430029L20 gene | 9430029L20Rik | |
| 1436519_a_at | 68832 | RIKEN cDNA 1110057K04 gene | 1110057K04Rik | |
| 1436766_at | 20822 | Sjogren syndrome antigen A2;Sjogren syndrome antigen A2 (60kDa, ribonucleoprotein autoantigen SS-A/Ro) | SSA2;Ssa2 | |
| 1436947_a_at | 53382 | thioredoxin-like;thioredoxin-like (32kD);thioredoxin-like, 32kDa | TXNL;Txnl | |
| 1436991_x_at | 227753 | gelsolin;gelsolin (amyloidosis, Finnish type) | GSN;Gsn | |

| | | | | |
|---|---|---|---|---|
| 1436994_a_at | 50708 | histone 1, H1c | HIST1H1C;Hist1h1c | [RefSeq Summary:] Histones are basic nuclear proteins that are responsible for the nucleosome structure of the chromosomal fiber in eukaryotes. Two molecules of each of the four core histones (H2A, H2B, H3, and H4) form an octamer, around which approximately 146 bp of DNA is wrapped in repeating units, called nucleosomes. The linker histone, H1, interacts with linker DNA between nucleosomes and functions in the compaction of chromatin into higher order structures. This gene is intronless and encodes a member of the histone H1 family. Transcripts from this gene lack polyA tails but instead contain a palindromic termination element. This gene is found in the large histone gene cluster on chromosome 6. |
| 1437082_at | 100986 | A kinase (PRKA) anchor protein (yotiao);A kinase (PRKA) anchor protein (yotiao) 9 | AKAP9;Akap9 | [RefSeq Summary:] The A-kinase anchor proteins (AKAPs) are a group of structurally diverse proteins which have the common function of binding to the regulatory subunit of protein kinase A (PKA) and confining the holoenzyme to discrete locations within the cell. This gene encodes a member of the AKAP family. Alternate splicing of this gene results in many isoforms that localize to the centrosome and the Golgi apparatus, and interact with numerous signaling proteins from multiple signal transduction pathways. These signaling proteins include type II protein kinase A, serine/threonine kinase protein kinase N, protein phosphatase 1, protein phosphatase 2a, protein kinase C-epsilon and phosphodiesterase 4D3. |
| 1437100_x_at | 223775 | proviral integration site 3;serine threonine kinase pim3 | Pim3 | |

| | | | | |
|---|---|---|---|---|
| 1437211_x_at | 68801 | ELOVL family member 5, elongation of long chain fatty acids (FEN1/Elo2, SUR4/Elo3-like, yeast);ELOVL family member 5, elongation of long chain fatty acids (yeast) | ELOVL5;Elovl5 | |
| 1437343_x_at | 170769 | TOB3-like | TOB3L | |
| 1437497_a_at | 15519 | heat shock 90kDa protein 1, alpha;heat shock protein 1, alpha | HSPCA;Hspca | |
| 1437513_a_at | 56442 | likely ortholog of mouse tumor differentially expressed 1, like;tumor differentially expressed 1, like | TDE2;Tde2 | |
| 1437624_x_at | 66911 | RIKEN cDNA 1110001K21 gene | 1110001K21Rik | |
| 1437626_at | | | | |
| 1437801_at | 21761 | mortality factor 4 like 1 | MORF4L1;Morf4l1 | |
| 1438033_at | 21685 | thyrotroph embryonic factor;thyrotrophic embryonic factor | TEF;Tef | [RefSeq Summary:] Thyrotroph embryonic factor (TEF), a transcription factor, is a member of the PAR (proline and acidic amino acid-rich) subfamily of basic region/leucine zipper (bZIP) transcription factors. It is expressed in a broad range of cells and |
| 1438040_a_at | 22027 | tumor rejection antigen (gp96) 1;tumor rejection antigen gp96 | TRA1;Tra1 | |

| | | | | |
|---|---|---|---|---|
| 1438116_x_at | 26941 | solute carrier family 9 (sodium/hydrogen exchanger), isoform 3 regulator 1;solute carrier family 9 (sodium/hydrogen exchanger), isoform 3 regulatory factor 1 | SLC9A3R1;Slc9a3r1 | [RefSeq Summary:] Members of the ezrin (VIL2; MIM 123900)-radixin (RDX; MIM 179410)-moesin (MSN; MIM 309845) (ERM) protein family are highly concentrated in the apical aspect of polarized epithelial cells. These cells are studded with microvilli containing bundles of actin filaments, which must attach to the membrane to assemble and maintain the microvilli. The ERM proteins, together with merlin, the NF2 (MIM 101000) gene product, are thought to be linkers between integral membrane and cytoskeletal proteins, and they bind directly to actin in vitro. Actin cytoskeleton reorganization requires the activation of a sodium/hydrogen exchanger (SLC9A3; MIM 182307). SLC9A3R1 is an ERM-binding protein.[supplied by OMIM] |
| 1438199_at | 20516 | solute carrier family 20 (phosphate transporter), member 2;solute carrier family 20, member 2 | SLC20A2;Slc20a2 | |
| 1438211_s_at | 13170 | D site albumin promoter binding protein;D site of albumin promoter (albumin D-box) binding protein | DBP;Dbp | |

| | | | | |
|---|---|---|---|---|
| 1438292_x_at | 11534 | adenosine kinase | ADK;Adk | [RefSeq Summary:] This gene encodes adenosine kinase, an abundant enzyme in mammalian tissues. The enzyme catalyzes the transfer of the gamma-phosphate from ATP to adenosine, thereby serving as a regulator of concentrations of both extracellular adenosine and intracellular adenine nucleotides. Adenosine has widespread effects on the cardiovascular, nervous, respiratory, and immune systems and inhibitors of the enzyme could play an important pharmacological role in increasing intravascular adenosine concentrations and acting as anti-inflammatory agents. Alternative splicing results in two transcript variants encoding different isoforms. Both isoforms of the enzyme phosphorylate adenosine with identical kinetics and both require Mg2+ for activity. |
| 1438322_x_at | 14137 | farnesyl diphosphate farnesyl transferase 1;farnesyl-diphosphate farnesyltransferase 1 | FDFT1;Fdft1 | |
| 1438386_x_at | 232087 | methionine adenosyltransferase II, alpha | MAT2A;Mat2a | |
| 1438902_a_at | 15519 | heat shock 90kDa protein 1, alpha;heat shock protein 1, alpha | HSPCA;Hspca | |
| 1439381_x_at | | | | |

| | | | | |
|---|---|---|---|---|
| 1439459_x_at | 104112 | ATP citrate lyase | ACLY;Acly | [RefSeq Summary:] ATP citrate lyase is the primary enzyme responsible for the synthesis of cytosolic acetyl-CoA in many tissues. The enzyme is a tetramer (relative molecular weight approximately 440,000) of apparently identical subunits. It catalyzes the formation of acetyl-CoA and oxaloacetate from citrate and CoA with a concomitant hydrolysis of ATP to ADP and phosphate. The product, acetyl-CoA, serves several important biosynthetic pathways, including lipogenesis and cholesterogenesis. In nervous tissue, ATP citrate-lyase may be involved in the biosynthesis of acetylcholine. Two transcript variants encoding distinct isoforms have been identified for this gene. |
| 1447462_at | | | | |
| 1448102_a_at | 66317 | RIKEN cDNA 2700038L12 gene | 2700038L12Rik | |
| 1448138_at | 110854 | protein phosphatase 2A, regulatory subunit B (PR 53);protein phosphatase 2A, regulatory subunit B' (PR 53) | PPP2R4;Ppp2r4 | [RefSeq Summary:] The product of this gene encodes a specific phosphotyrosyl phosphatase activator of the dimeric form of protein phosphatase 2A, which is composed of a catalytic subunit and a constant regulatory subunit. Protein phosphatase 2A is one of the four major Ser/Thr phosphatases, and it is implicated in the negative control of cell growth and division. Association of this protein with protein phosphatase 2A heterodimeric core enzyme results in the formation of a specific trimeric holoenzyme. Alternatively spliced variants encoding different isoforms have been identified for this gene. |
| 1448151_at | 15568 | ELAV (embryonic lethal, abnormal vision, Drosophila)-like 1 (Hu antigen R) | ELAVL1;Elavl1 | [RefSeq Summary:] The protein encoded by this gene is a member of the ELAVL protein family. This encoded protein contains 3 RNA-binding domains and binds cis-acting AU-rich elements. It destabilizes mRNAs and thereby regulates gene expression. |

| | | | | |
|---|---|---|---|---|
| 1448178_a_at | 12462 | chaperonin containing TCP1, subunit 3 (gamma);chaperonin subunit 3 (gamma);similar to chaperonin subunit 3 (gamma) | CCT3;Cct3 | |
| 1448185_at | 64209 | homocysteine-inducible, endoplasmic reticulum stress-inducible, ubiquitin-like domain member 1 | HERPUD1;Herpud1 | |
| 1448213_at | 16952 | annexin 1;annexin A1 | ANXA1;Anxa1 | [RefSeq Summary:] Annexin I belongs to a family of Ca(2+)-dependent phospholipid binding proteins which have a molecular weight of approximately 35,000 to 40,000 and are preferentially located on the cytosolic face of the plasma membrane. Annexin I protein has an apparent relative molecular mass of 40 kDa, with phospholipase A2 inhibitory activity. Since phospholipase A2 is required for the biosynthesis of the potent mediators of inflammation, prostaglandins and leukotrienes, annexin I may have potential anti-inflammatory activity. |
| 1448219_a_at | 22631 | Tyrosine 3-monooxygenase/tryptophan 5-monooxygenase activation protein, zeta polypeptide;tyrosine 3-monooxygenase/tryptophan 5-monooxygenase activation protein, zeta polypeptide | YWHAZ;Ywhaz | [RefSeq Summary:] This gene product belongs to the 14-3-3 family of proteins which mediate signal transduction by binding to phosphoserine-containing proteins. This highly conserved protein family is found in both plants and mammals, and this protein is 99% identical to the mouse, rat and sheep orthologs. The encoded protein interacts with IRS1 protein, suggesting a role in regulating insulin sensitivity. Two transcript variants differing in the 5' UTR, but encoding the same protein, have been identified for this gene. |
| 1448230_at | 22224 | ubiquintin c-terminal hydrolase related polypeptide;ubiquitin specific protease 10 | USP10;Usp10 | |

| | | | | |
|---|---|---|---|---|
| 1448233_at | 19122 | prion protein;prion protein (p27-30) (Creutzfeld-Jakob disease, Gerstmann-Strausler-Scheinker syndrome, fatal familial insomnia) | PRNP;Prnp | [RefSeq Summary:] The protein encoded by this gene is a membrane glycosylphosphatidylinositol-anchored glycoprotein that tends to aggregate into rod-like structures. The encoded protein contains a highly unstable region of five tandem octapeptide repeats. This gene is found on chromosome 20, approximately 20 kbp upstream of a gene which encodes a biochemically and structurally similar protein to the one encoded by this gene. Mutations in the repeat region as well as elsewhere in this gene have been associated with Creutzfeldt-Jakob disease, fatal familial insomnia, Gerstmann-Straussler disease, Huntington disease-like 1, and kuru. Two transcript variants encoding the same protein have been found for this gene. |
| 1448276_at | 64540 | transmembrane 4 superfamily member 7 | TM4SF7;Tm4sf7 | [RefSeq Summary:] The protein encoded by this gene is a member of the transmembrane 4 superfamily, also known as the tetraspanin family. Most of these members are cell-surface proteins that are characterized by the presence of four hydrophobic domains. The proteins mediate signal transduction events that play a role in the regulation of cell development, activation, growth and motility. This encoded protein is a cell surface glycoprotein and is similar in sequence to its family member CD53 antigen. It is known to complex with integrins and other transmembrane 4 superfamily proteins. |
| 1448306_at | 18035 | nuclear factor of kappa light chain gene enhancer in B-cells inhibitor, alpha;nuclear factor of kappa light polypeptide gene enhancer in B-cells inhibitor, alpha | NFKBIA;Nfkbia | |

| | | | | |
|---|---|---|---|---|
| 1448309_at | 55946 | adaptor-related protein complex 3, mu 1 subunit;adaptor-related protein complex AP-3, mu 1 subunit | AP3M1;Ap3m1 | [RefSeq Summary:] This gene encodes a subunit of the heterotetrameric adaptor-related protein comlex 3 (AP-3), which belongs to the adaptor complexes medium subunits family. The AP-3 complex plays a role in protein trafficking to lysosomes and specialized organelles. It is observed that mutations of this encoded protein is associated with Hermansky-Pudlak syndrome. |
| 1448348_at | 53872 | GPI-anchored membrane protein 1 | Gpiap1 | |
| 1448370_at | 22241 | Unc-51 like kinase 1;Unc-51 like kinase 1 (C. elegans);unc-51-like kinase 1 (C. elegans) | ULK1;Ulk1 | |
| 1448390_a_at | 20148 | retinal short-chain dehydrogenase/reductase 1;short-chain dehydrogenase/reductase 1 | DHRS3;Dhrs3 | |
| 1448448_a_at | 12651 | choline kinase;choline kinase-like;choline/ethanolamine kinase;ethanolamine kinase | CHK;CHKL;Chetk;Chk;Chkl;EKI1 | [RefSeq Summary:] Choline kinase (CK) and ethanolamine kinase (EK) catalyze the phosphorylation of choline/ethanolamine to phosphocholine/phosphoethanolamine. This is the first enzyme in the biosynthesis of phosphatidylcholine/phosphatidylethanolamine in |
| 1448450_at | 11637 | Adenylate kinase 1;Adenylate kinase 2;adenylate kinase 1;adenylate kinase 2;adenylate kinase 3;adenylate kinase 4;adenylate kinase 5 | AK1;AK2;AK3;AK5;Ak1;Ak2;Ak4 | [RefSeq Summary:] Adenylate kinase is an enzyme involved in regulating the adenine nucleotide composition within a cell by catalyzing the reversible transfer of phosphate group among adinine nucleotides. Three isozymes of adenylate kinase have been identi |

| | | | | |
|---|---|---|---|---|
| 1448454_at | 67996 | splicing factor, arginine/serine-rich 6 | SFRS6;Sfrs6 | [RefSeq Summary:] The protein encoded by this gene is involved in mRNA splicing and may play a role in the determination of alternative splicing. The encoded nuclear protein belongs to the splicing factor SR family and has been shown to bind with and modulate another member of the family, SFRS12. |
| 1448619_at | 13360 | 7-dehydrocholesterol reductase | DHCR7;Dhcr7 | |
| 1448641_at | 103537 | mbt domain containing 1 | MBTD1;Mbtd1 | [Proteome Summary:] Region of moderate similarity to a region of SCML2; related protein is a member of the Polycomb group |
| 1448647_at | 17158 | Mannosidase 2, alpha 1;mannosidase 2, alpha 1;mannosidase, alpha, class 2A, member 1;mannosidase, alpha, class 2A, member 2 | MAN2A1;MAN2A2;Man2a1 | |
| 1448655_at | 16971 | low density lipoprotein receptor-related protein 1;low density lipoprotein-related protein 1 (alpha-2-macroglobulin receptor) | LRP1;Lrp1 | |

| | | | | |
|---|---|---|---|---|
| 1448681_at | 16169 | interleukin 15 receptor, alpha;interleukin 15 receptor, alpha chain | IL15RA;Il15ra | [RefSeq Summary:] The protein encoded by this gene is a cytokine receptor that specifically binds IL15 with high affinity. The receptors of IL15 and IL2 share two subunits, the IL2R beta and IL2R gamma chains. This forms the basis of many overlapping biological activities of IL15 and IL2. The IL2 receptor requires an additional IL2-specific alpha subunit for high affinity IL2 binding. This protein is structurally related to IL2R alpha, but is capable of binding IL15 with high affinity independent of other subunits, which suggests the distinct roles between IL15 and IL2. This receptor is reported to enhance cell proliferation and expression of apoptosis inhibitor BCL2L1/BCL2-XL and BCL2. Multiple alternatively spliced transcript variants of this gene have been reported. The full length sequences of only two variants encoding distinct isoforms are available. |
| 1448682_at | 56455 | dynein, cytoplasmic, light chain 1 | Dnclc1 | |
| 1448729_a_at | 18952 | septin 4 | 4-Sep | |

| | | | | |
|---|---|---|---|---|
| 1448830_at | 19252 | Osteotesticular phosphatase;Protein tyrosine phosphatase, receptor type, A;Protein tyrosine phosphatase, receptor type, J;X-linked myotubular myopathy gene 1;acid phosphatase 1, soluble;cell division cycle 25 homolog A (S. cerevisiae);cell division cycle | ACP1;Acp1;CDC25A;CDC25B;CDC25C;CDKN3;Cdc25a;Cdc25b;Cdc25c;DUSP1;DUSP10;DUSP11;DUSP12;DUSP13;DUSP14;DUSP15;DUSP18;DUSP19;DUSP2;DUSP3;DUSP4;DUSP5;DUSP6;DUSP8;DUSP9;Dusp1;Dusp12;Dusp13;Dusp14;Dusp2;Dusp3;Dusp6;Dusp7;Dusp8;Esp;Hcph;Lcptp;MTM1;MTMR3;Mtm1;PTEN;PTP9Q22;PTPN1;PTPN11;PTPN12;PTPN13;PTPN14;PTPN18;PTPN2;PTPN21;PTPN22;PTPN3;PTPN4;PTPN5;PTPN6;PTPN7;PTPN9 | [Proteome Summary:] Dual-specificity protein phosphatase for testis and skeletal muscle; acts on P-serine,P-threonine and P-tyrosine of myelin basic protein ;[Proteome Summary:] Receptor-type protein tyrosine phosphatase Z1; similar to carbonic anhydras |
| 1448844_at | 66427 | cytochrome b5 outer mitochondrial membrane precursor | 1810044O22Rik | |
| 1448864_at | 20623 | SNF related kinase;SNF-1 related kinase | SNRK;Snrk | |
| 1448875_at | 22770 | zinc fingers and homeoboxes protein 1;zinc-fingers and homeoboxes 1 | ZHX1;Zhx1 | |

| | | | | |
|---|---|---|---|---|
| 1448997_at | 19157 | pleckstrin homology, Sec7 and coiled-coil domains 1;pleckstrin homology, Sec7 and coiled-coil domains 1(cytohesin 1);pleckstrin homology, Sec7 and coiled/coil domains 1 | PSCD1;Pscd1 | [RefSeq Summary:] Pleckstrin homology, Sec7 and coiled/coil domains 1 (PSCD1) is a member of the PSCD family. Members of this family have identical structural organization that consists of an N-terminal coiled-coil motif, a central Sec7 domain, and a C-terminal pleckstrin homology (PH) domain. The coiled-coil motif is involved in homodimerization, the Sec7 domain contains guanine-nucleotide exchange protein (GEP) activity, and the PH domain interacts with phospholipids and is responsible for association of PSCDs with membranes. Members of this family appear to mediate the regulation of protein sorting and membrane trafficking. The PSCD1 is highly expressed in natural killer and peripheral T cells, and regulates the adhesiveness of integrins at the plasma membrane of lymphocytes. PSCD1 protein is 83% homologous to PSCD2. |
| 1449010_at | 18415 | osmotic stress protein;similar to osmotic stress protein 94 kDa | OSP94;Osp94 | |
| 1449039_a_at | 50926 | heterogeneous nuclear ribonucleoprotein D-like | HNRPDL;Hnrpdl | [RefSeq Summary:] This gene belongs to the subfamily of ubiquitously expressed heterogeneous nuclear ribonucleoproteins (hnRNPs). The hnRNPs are RNA binding proteins and they complex with heterogeneous nuclear RNA (hnRNA). These proteins are associated with pre-mRNAs in the nucleus and appear to influence pre-mRNA processing and other aspects of mRNA metabolism and transport. While all of the hnRNPs are present in the nucleus, some seem to shuttle between the nucleus and the cytoplasm. The hnRNP proteins have distinct nucleic acid binding properties. The protein encoded by this gene has two RRM domains that bind to RNAs. Two alternatively spliced transcript variants have been described for this gene. The variants encode the same protein but have different 3' UTRs. This protein is similar to its family member HNRPD. |

| | | | | |
|---|---|---|---|---|
| 1449051_at | 19013 | Peroxisome proliferator activated receptor alpha;peroxisome proliferative activated receptor, alpha;peroxisome proliferator activated receptor alpha | PPARA;Ppara | [RefSeq Summary:] Peroxisome proliferators are a diverse group of chemicals which include hypolipidemic drugs, herbicides, leukotriene antagonists, and plasticizers, and are so called because they induce an increase in the size and number of peroxisomes. |
| 1449089_at | 268903 | nuclear receptor interacting protein 1 | NRIP1;Nrip1 | [RefSeq Summary:] Nuclear receptor interacting protein 1 (NRIP1) is a nuclear protein that specifically interacts with the hormone-dependent activation domain AF2 of nuclear receptors. Also known as RIP140, this protein modulates transcriptional activity of the estrogen receptor. |
| 1449110_at | 11852 | ras homolog gene family, member AB;ras homolog gene family, member B;rhoB gene | ARHB;Arhb | |
| 1449183_at | 12846 | catechol-O-methyltransferase | COMT;Comt | [RefSeq Summary:] Catechol-O-methyltransferase catalyzes the transfer of a methyl group from S-adenosylmethionine to catecholamines, including the neurotransmitters dopamine, epinephrine, and norepinephrine. This O-methylation results in one of the major degradative pathways of the catecholamine transmitters. In addition to its role in the metabolism of endogenous substances, COMT is important in the metabolism of catechol drugs used in the treatment of hypertension, asthma, and Parkinson disease. COMT is found in two forms in tissues, a soluble form (S-COMT) and a membrane-bound form (MB-COMT). The differences between S-COMT and MB-COMT reside within the N-termini. The transcript variants are formed through the use of alternative translation initiation sites and promoters. |

| | | | | |
|---|---|---|---|---|
| 1449194_at | 64658 | mitochondrial ribosomal protein S25 | MRPS25;Mrps25 | [RefSeq Summary:] Mammalian mitochondrial ribosomal proteins are encoded by nuclear genes and help in protein synthesis within the mitochondrion. Mitochondrial ribosomes (mitoribosomes) consist of a small 28S subunit and a large 39S subunit. They have an estimated 75% protein to rRNA composition compared to prokaryotic ribosomes, where this ratio is reversed. Another difference between mammalian mitoribosomes and prokaryotic ribosomes is that the latter contain a 5S rRNA. Among different species, the proteins comprising the mitoribosome differ greatly in sequence, and sometimes in biochemical properties, which prevents easy recognition by sequence homology. This gene encodes a 28S subunit protein. A pseudogene corresponding to this gene is found on chromosome 4. |
| 1449209_a_at | 17252 | retinol dehydrogenase 11;retinol dehydrogenase 11 (all-trans and 9-cis) | RDH11;Rdh11 | |
| 1449324_at | 50527 | ERO1-like (S. cerevisiae) | ERO1L;Ero1l | [RefSeq Summary:] This gene encodes a protein similar to a Saccharomyces cerevisiae protein which is required for oxidative protein folding. A similar human protein is an integral membrane protein in the endoplasmic reticulum (ER) and is thought to be involved in oxidative ER protein folding. |
| 1449335_at | 21859 | Tissue inhibitor of metalloproteinase 3;tissue inhibitor of metalloproteinase 3;tissue inhibitor of metalloproteinase 3 (Sorsby fundus dystrophy, pseudoinflammatory) | TIMP3;Timp3 | [RefSeq Summary:] This gene belongs to the TIMP gene family. The proteins encoded by this gene family are natural inhibitors of the matrix metalloproteinases, a group of peptidases involved in degradation of the extracellular matrix. The expression of this gene is induced in response to mitogenic stimulation and subject to cell cycle regulation. Mutations in this gene cause the autosomal dominant disorder Sorsby's fundus dystrophy. |
| 1449568_at | 83379 | klotho beta | AV071179 | |

| | | | | |
|---|---|---|---|---|
| 1449576_at | 66235 | RIKEN cDNA 1500010B24 gene;eukaryotic translation initiation factor 1A;eukaryotic translation initiation factor 1A, Y chromosome | EIF1A;EIF1AY;Eif1a;Eif1ay | [RefSeq Summary:] This gene encodes a protein similar to eukaryotic translation initiation factor 1A (EIF1A). EIF1A is required for the binding of the 43S complex (a 40S subunit, eIF2/GTP/Met-tRNAi and eIF3) to the 5' end of capped RNA. ;[RefSeq Summary:] This gene encodes an essential eukaryotic translation initiation factor. The protein is required for the binding of the 43S complex (a 40S subunit, eIF2/GTP/Met-tRNAi and eIF3) to the 5' end of capped RNA. |
| 1449731_s_at | 18035 | nuclear factor of kappa light chain gene enhancer in B-cells inhibitor, alpha;nuclear factor of kappa light polypeptide gene enhancer in B-cells inhibitor, alpha | NFKBIA;Nfkbia | |
| 1449799_s_at | 67451 | RIKEN cDNA 1200008D14 gene | 1200008D14Rik | |
| 1449851_at | 18626 | period 1;period homolog 1 (Drosophila);period homolog 2;period homolog 2 (Drosophila);period homolog 3 (Drosophila) | PER1;PER2;PER3;Per1;Per2;Per3 | [RefSeq Summary:] This gene is a member of the Period family of genes and is expressed in a circadian pattern in the suprachiasmatic nucleus, the primary circadian pacemaker in the mammalian brain. Genes in this family encode components of the circadian r |

| | | | | |
|---|---|---|---|---|
| 1450136_at | 12494 | CD38 antigen;CD38 antigen (p45);bone marrow stromal cell antigen 1 | BST1;Bst1;CD38;Cd38 | [RefSeq Summary:] Bone marrow stromal cell antigen-1 is a stromal cell line-derived glycosylphosphatidylinositol-anchored molecule that facilitates pre-B-cell growth. The deduced amino acid sequence exhibits 33% similarity with CD38. BST1 expression is enhanced in bone marrow stromal cell lines derived from patients with rheumatoid arthritis. The polyclonal B-cell abnormalities in rheumatoid arthritis may be, at least in part, attributed to BST1 overexpression in the stromal cell population. ;[RefSeq Summary:] CD38 is a novel multifunctional ectoenzyme widely expressed in cells and tissues especially in leukocytes. CD38 also functions in cell adhesion,signal transduction and calcium signaling [PROW] |
| 1450184_s_at | 21685 | thyrotroph embryonic factor;thyrotrophic embryonic factor | TEF;Tef | [RefSeq Summary:] Thyrotroph embryonic factor (TEF), a transcription factor, is a member of the PAR (proline and acidic amino acid-rich) subfamily of basic region/leucine zipper (bZIP) transcription factors. It is expressed in a broad range of cells and |
| 1450259_a_at | 20850 | Signal transducer and activator of transcription 5a;signal transducer and activator of transcription 5A | STAT5A;Stat5a | [RefSeq Summary:] The protein encoded by this gene is a member of the STAT family of transcription factors. In response to cytokines and growth factors, STAT family members are phosphorylated by the receptor associated kinases, and then form homo- or heterodimers that translocate to the cell nucleus where they act as transcription activators. This protein is activated by, and mediates the responses of many cell ligands, such as IL2, IL3, IL7 GM-CSF, erythropoietin, thrombopoietin, and different growth hormones. Activation of this protein in myeloma and lymphoma associated with a TEL/JAK2 gene fusion is independent of cell stimulus and has been shown to be essential for the tumorigenesis. The mouse counterpart of this gene is found to induce the expression of BCL2L1/BCL-X(L), which suggests the antiapoptotic function of this gene in cells. |
| 1450391_a_at | 23945 | monoglyceride lipase | MGLL;Mgll | |

| | | | | |
|---|---|---|---|---|
| 1450395_at | 20520 | solute carrier family 22 (organic cation transporter), member 5;solute carrier family 22, member 5 | SLC22A5;Slc22a5 | [RefSeq Summary:] Polyspecific organic cation transporters in the liver, kidney, intestine, and other organs are critical for elimination of many endogenous small organic cations as well as a wide array of drugs and environmental toxins. The encoded protein is a plasma integral membrane protein which functions both as an organic cation transporter and as a sodium-dependent high affinity carnitine transporter. The encoded protein is involved in the active cellular uptake of carnitine. Mutations in this gene are the cause of systemic primary carnitine deficiency (CDSP), an autosomal recessive disorder manifested early in life by hypoketotic hypoglycemia and acute metabolic decompensation, and later in life by skeletal myopathy or cardiomyopathy. |
| 1450430_at | 17533 | mannose receptor, C type 1 | MRC1;Mrc1 | [RefSeq Summary:] The recognition of complex carbohydrate structures on glycoproteins is an important part of several biological processes, including cell-cell recognition, serum glycoprotein turnover, and neutralization of pathogens. The protein encoded by this gene is a type I membrane receptor that mediates the endocytosis of glycoproteins by macrophages. The protein has been shown to bind high-mannose structures on the surface of potentially pathogenic viruses, bacteria, and fungi so that they can be neutralized by phagocytic engulfment. |

| | | | | |
|---|---|---|---|---|
| 1450643_s_at | 14081 | fatty acid Coenzyme A ligase, long chain 2;fatty acid Coenzyme A ligase, long chain 3;fatty acid Coenzyme A ligase, long chain 4;fatty acid Coenzyme A ligase, long chain 5;fatty acid-Coenzyme A ligase, long chain 4;fatty-acid-Coenzyme A ligase, long-chain 1;fatty-acid-Coenzyme A ligase, long-chain 2;fatty-acid-Coenzyme A ligase, long-chain 3;fatty-acid-Coenzyme A ligase, long-chain 4;fatty-acid-Coenzyme A ligase, long-chain 5;fatty-acid-Coenzyme A | FACL2;FACL3;FACL4;FACL5;FACL6;Facl2;Facl3;Facl4;Facl5 | [RefSeq Summary:] The protein encoded by this gene is an isozyme of the long-chain fatty-acid-coenzyme A ligase family. Although differing in substrate specificity, subcellular localization, and tissue distribution, all isozymes of this family convert fre |
| 1450699_at | 20341 | selenium binding protein 1 | SELENBP1;Selenbp1 | [RefSeq Summary:] This gene product belongs to the selenium-binding protein family. Selenium is an essential nutrient that exhibits potent anticarcinogenic properties, and deficiency of selenium may cause certain neurologic diseases. It has been proposed that the effects of selenium in preventing cancer and neurologic diseases may be mediated by selenium-binding proteins. The exact function of this gene is not known. |

| | | | | |
|---|---|---|---|---|
| 1450714_at | 54375 | ornithine decarboxylase antizyme inhibitor | OAZIN;Oazin | [RefSeq Summary:] Ornithine decarboxylase (ODC) catalyzes the conversion of ornithine to putrescine in the first and apparently rate-limiting step in polyamine biosynthesis. Ornithine decarboxylase antizymes play a role in the regulation of polyamine synthesis by binding to and inhibiting ornithine decarboxylase. The protein encoded by this gene is highly similar to ODC. It binds to ODC antizyme and stabilizes ODC, thus inhibiting antizyme-mediated ODC degradation. Two alternatively spliced transcript variants have been found for this gene. |
| 1450776_at | 102247 | expressed sequence AU041707 | AU041707 | |
| 1450843_a_at | 12406 | serine (or cysteine) proteinase inhibitor, clade H (heat shock protein 47), member 1, (collagen binding protein 1);serine (or cysteine) proteinase inhibitor, clade H, member 1 | SERPINH1;Serpinh1 | [RefSeq Summary:] This gene encodes a member of the serpin superfamily of serine proteinase inhibitors. Its expression is induced by heat shock. The protein localizes to the endoplasmic reticulum lumen and binds collagen; thus it is thought to be a molecular chaperone involved in the maturation of collagen molecules. Autoantibodies to this protein have been found in patients with rheumatoid arthritis. |
| 1450845_a_at | 66882 | basic leucine zipper and W2 domains 1 | BZW1;Bzw1 | |

| | | | | |
|---|---|---|---|---|
| 1450869_at | 14164 | fibroblast growth factor 1;fibroblast growth factor 1 (acidic) | FGF1;Fgf1 | [RefSeq Summary:] The protein encoded by this gene is a member of the fibroblast growth factor (FGF) family. FGF family members possess broad mitogenic and cell survival activities, and are involved in a variety of biological processes, including embryonic development, cell growth, morphogenesis, tissue repair, tumor growth and invasion. This protein functions as a modifier of endothelial cell migration and proliferation, as well as an angiogenic factor. It acts as a mitogen for a variety of mesoderm- and neuroectoderm-derived cells in vitro, thus is thought to be involved in organogenesis. Three alternatively spliced variants encoding different isoforms have been described. |
| 1450877_at | 79555 | RIKEN cDNA 8030460C05 gene | BC005537 | |
| 1451006_at | 22436 | xanthine dehydrogenase | XDH;Xdh | [RefSeq Summary:] Xanthine dehydrogenase belongs to the group of molybdenum-containing hydroxylases involved in the oxidative metabolism of purines. The enzyme is a homodimer. Xanthine dehydrogenase can be converted to xanthine oxidase by reversible sulfhydryl oxidation or by irreversible proteolytic modification. Defects in xanthine dehydrogenase cause xanthinuria, may contribute to adult respiratory stress syndrome, and may potentiate influenza infection through an oxygen metabolite-dependent mechanism. |
| 1451018_at | 68192 | leptin receptor overlapping transcript-like 1 | LEPROTL1;Leprotl1 | |
| 1451042_a_at | 67014 | myc induced nuclear antigen | Mina | |
| 1451069_at | 223775 | proviral integration site 3;serine threonine kinase pim3 | Pim3 | |

| | | | | |
|---|---|---|---|---|
| 1451074_at | 24017 | ring finger protein 13 | RNF13;Rnf13 | [RefSeq Summary:] The protein encoded by this gene contains a RING zinc finger, a motif known to be involved in protein-protein interactions. The specific function of this gene has not yet been determined. Five alternatively spliced transcript variants encoding three distinct isoforms have been reported. |
| 1451122_at | 207933 | isopentenyl-diphosphate delta-isomerase-like | LOC207933 | |
| 1451177_at | 67035 | RIKEN cDNA 2010306G19 gene | 2010306G19Rik | |
| 1451200_at | 16561 | kinesin family member 1B | KIF1B;Kif1b | |
| 1451208_at | 225363 | eukaryotic translation termination factor 1;hypothetical protein MGC18745 | ETF1;Etf1 | |
| 1451285_at | 233908 | fusion, derived from t(12;16) malignant liposarcoma;pigpen | FUS;Fus | |
| 1451286_s_at | 233908 | fusion, derived from t(12;16) malignant liposarcoma;pigpen | FUS;Fus | |

| | | | | |
|---|---|---|---|---|
| 1451310_a_at | 13039 | Cathepsin L;cathepsin L | CTSL;Ctsl | [RefSeq Summary:] The protein encoded by this gene is a lysosomal cysteine proteinase that plays a major role in intracellular protein catabolism. Its substrates include collagen and elastin, as well as alpha-1 protease inhibitor, a major controlling element of neutrophil elastase activity. The encoded protein has been implicated in several pathologic processes, including myofibril necrosis in myopathies and in myocardial ischemia, and in the renal tubular response to proteinuria. This protein, which is a member of the peptidase C1 family, is a dimer composed of disulfide-linked heavy and light chains, both produced from a single protein precursor. At least two transcript variants encoding the same protein have been found for this gene. |
| 1451322_at | 69574 | RIKEN cDNA 2310016A09 gene | 2310016A09Rik | |
| 1451350_a_at | 230514 | OB-receptor gene related protein (OB-RGRP);leptin receptor gene-related protein | OBRGRP;Obrgrp | |
| 1451392_at | 232089 | RIKEN cDNA C330008I15 gene | C330008I15Rik | |
| 1451457_at | 235293 | sterol-C5-desaturase (ERG3 delta-5-desaturase homolog, fungal)-like;sterol-C5-desaturase (fungal ERG3, delta-5-desaturase) homolog (S. cerevisae);sterol-C5-desaturase (fungal ERG3, delta-5-desaturase)-like | SC5DL;Sc5d | [RefSeq Summary:] This gene encodes an enzyme that is involved in cholesterol biosynthesis. It is thought to be an integral membrane protein. |
| 1451465_at | 69459 | RIKEN cDNA 2300004C15 gene | 2300004C15Rik | |

| | | | | |
|---|---|---|---|---|
| 1451714_a_at | 26397 | mitogen activated protein kinase kinase 3;mitogen-activated protein kinase kinase 3 | MAP2K3;Map2k3 | [RefSeq Summary:] The protein encoded by this gene is a dual specificity protein kinase that belongs to the MAP kinase kinase family. This kinase is activated by mitogenic and environmental stress, and participates in the MAP kinase-mediated signaling cascade. It phosphorylates and thus activates MAPK14/p38-MAPK. This kinase can be activated by insulin, and is necessary for the expression of glucose transporter. Expression of RAS oncogene is found to result in the accumulation of the active form of this kinase, which thus leads to the constitutive activation of MAPK14, and confers oncogenic transformation of primary cells. The inhibition of this kinase is involved in the pathogenesis of Yersina pseudotuberculosis. Three alternatively spliced transcript variants of this gene encoding distinct isoforms have been reported. |
| 1451742_a_at | 216558 | UDP-glucose pyrophosphorylase 2 | UGP2;Ugp2 | [RefSeq Summary:] The enzyme encoded by this gene is an important intermediary in mammalian carbohydrate interconversions. It transfers a glucose moiety from glucose-1-phosphate to MgUTP and forms UDP-glucose and MgPPi. In liver and muscle tissue, UDP-glucose is a direct precursor of glycogen; in lactating mammary gland it is converted to UDP-galactose which is then converted to lactose. The eukaryotic enzyme has no significant sequence similarity to the prokaryotic enzyme. Transcript variants have been described but are not supported by experimental evidence. |
| 1451770_s_at | 13211 | DEAH (Asp-Glu-Ala-His) box polypeptide 9 | DHX9;Dhx9 | [RefSeq Summary:] DEAD box proteins, characterized by the conserved motif Asp-Glu-Ala-Asp (DEAD), are putative RNA helicases. They are implicated in a number of cellular processes involving alteration of RNA secondary structure such as translation initiat |

| | | | | |
|---|---|---|---|---|
| 1451899_a_at | 57080 | GTF2I repeat domain containing 1;general transcription factor II I;general transcription factor II I repeat domain-containing 1;general transcription factor II, i | GTF2I;GTF2IRD1;Gtf2i;Gtf2ird1 | [RefSeq Summary:] The protein encoded by this gene contains five GTF2I-like repeats and each repeat possesses a potential helix-loop-helix (HLH) motif. It may have the ability to interact with other HLH-proteins and function as a transcription factor or a |
| 1452035_at | 12826 | collagen, type IV, alpha 1;procollagen, type IV, alpha 1 | COL4A1;Col4a1 | [RefSeq Summary:] This gene encodes the major type IV alpha collagen chain of basement membranes. Like the other members of the type IV collagen gene family, this gene is organized in a head-to-head conformation with another type IV collagen gene so that each gene pair shares a common promoter. |
| 1452047_at | 12301 | calcyclin binding protein | Cacybp | |
| 1452071_at | | | | |
| 1452094_at | 18451 | procollagen-proline, 2-oxoglutarate 4-dioxygenase (proline 4-hydroxylase), alpha 1 polypeptide;procollagen-proline, 2-oxoglutarate 4-dioxygenase (proline 4-hydroxylase), alpha polypeptide I;prolyl 4-hydroxylase alpha subunit | P4HA1;P4ha1 | |
| 1452145_at | 100198 | hexose-6-phosphate dehydrogenase (glucose 1-dehydrogenase) | H6PD;H6pd | [RefSeq Summary:] There are 2 forms of glucose-6-phosphate dehydrogenase. G form is X-linked and H form, encoded by this gene, is autosomally linked. This H form shows activity with other hexose-6-phosphates, especially galactose-6-phosphate, whereas the G form is specific for glucose-6-phosphate. Both forms are present in most tissues, but H form is not found in red cells. |

| | | | | |
|---|---|---|---|---|
| 1452179_at | 269424 | PHD protein Jade-1;RIKEN cDNA D530048A03 gene | JADE1;Jade1 | |
| 1452203_at | 109019 | RIKEN cDNA 5830411E10 gene | 5830411E10Rik | |
| 1452264_at | 209039 | tensin like C1 domain-containing phosphatase | TENC1;Tenc1 | [RefSeq Summary:] The protein encoded by this gene belongs to the tensin family. Tensin is a focal adhesion molecule that binds to actin filaments and participates in signaling pathways. This protein plays a role in regulating cell migration. Alternative splicing occurs at this locus and three transcript variants encoding three distinct isoforms have been identified. |
| 1452291_at | 212285 | centaurin, delta 1;hypothetical protein LOC212285 | CENTD1;Centd1 | [RefSeq Summary:] The protein encoded by this gene contains ARF-GAP, RHO-GAP, ankyrin repeat, RAS-associating, and pleckstrin homology domains. This protein lacks the predicted catalytic arginine in the RHO-GAP domain and is therefore unlikely to have RHO-GAP activity. While the encoded protein does contain a sterile alpha motif (SAM) commonly found in some signaling molecules, the function of the protein has not been determined. Two transcript variants encoding different isoforms have been found for this gene. |
| 1452298_a_at | 17919 | Glutamate decarboxylase 1 (brain);Glutamate decarboxylase 2 (islet);glutamate decarboxylase 1 (brain, 67kDa);glutamate decarboxylase 2 (pancreatic islets and brain, 65kDa);glutamic acid decarboxylase 1;glutamic acid decarboxylase 2;myosin 5B;myosin VB;myosin Vb | GAD1;GAD2;Gad1;Gad2;MYO5B;Myo5b | [RefSeq Summary:] This gene encodes one of several forms of glutamic acid decarboxylase, identified as a major autoantigen in insulin-dependent diabetes. The enzyme encoded is responsible for catalyzing the production of gamma-aminobutyric acid from L-gl |

| | | | | |
|---|---|---|---|---|
| 1452309_at | 68178 | RIKEN cDNA 4933421H10 gene | 4933421H10Rik | |
| 1452545_a_at | 16412 | Integrin, beta 1;integrin beta 1 (fibronectin receptor beta);integrin, beta 1 (fibronectin receptor, beta polypeptide, antigen CD29 includes MDF2, MSK12) | ITGB1;Itgb1 | [RefSeq Summary:] Integrins are heterodimeric proteins made up of alpha and beta subunits. At least 18 alpha and 8 beta subunits have been described in mammals. Integrin family members are membrane receptors involved in cell adhesion and recognition in a variety of processes including embryogenesis, hemostasis, tissue repair, immune response and metatastatic diffusion of tumor cells. The protein encoded by this gene is a beta subunit. Six alternatively spliced variants have been found for this gene which encode five proteins with alternate carboxy termini. |
| 1452646_at | 68728 | RIKEN cDNA 1110029F20 gene | 1110029F20Rik | |
| 1452661_at | 22042 | transferrin receptor;transferrin receptor (p90, CD71) | TFRC;Tfrc | |
| 1452828_at | 231670 | F-box only protein 21 | FBXO21;Fbxo21 | [RefSeq Summary:] This gene encodes a member of the F-box protein family which is characterized by an approximately 40 amino acid motif, the F-box. The F-box proteins constitute one of the four subunits of ubiquitin protein ligase complex called SCFs (SKP1-cullin-F-box), which function in phosphorylation-dependent ubiquitination. The F-box proteins are divided into 3 classes: Fbws containing WD-40 domains, Fbls containing leucine-rich repeats, and Fbxs containing either different protein-protein interaction modules or no recognizable motifs. The protein encoded by this gene belongs to the Fbxs class. Alternative splicing of this gene generates 2 transcript variants. |

| | | | | |
|---|---|---|---|---|
| 1453851_a_at | 23882 | growth arrest and DNA-damage-inducible 45 gamma;growth arrest and DNA-damage-inducible, gamma | GADD45G;Gadd45g | [RefSeq Summary:] This gene is a member of a group of genes whose transcript levels are increased following stressful growth arrest conditions and treatment with DNA-damaging agents. The protein encoded by this gene responds to environmental stresses by mediating activation of the p38/JNK pathway via MTK1/MEKK4 kinase. The GADD45G is highly expressed in placenta. |
| 1454161_s_at | 58520 | open reading frame 11 | ORF11 | |
| 1454610_at | 235072 | septin 7 | 7-Sep | |
| 1454682_at | 97159 | RIKEN cDNA A430005L14 gene | A430005L14Rik | |
| 1454704_at | 12492 | scavenger receptor class B, member 2 | SCARB2;Scarb2 | [RefSeq Summary:] The protein encoded by this gene is a type III glycoprotein that is located primarily in limiting membranes of lysosomes and endosomes. Studies of the similar protein in mice and rat suggested that this protein may participate in membrane transportation and the reorganization of endosomal/lysosomal compartment. Deficiency of the similar protein in mice was reported to impair cell membrane transport processes and cause pelvic junction obstruction, deafness, and peripheral neuropathy. |
| 1454712_at | 230125 | hypothetical protein D130005A03 | D130005A03 | |

| | | | | |
|---|---|---|---|---|
| 1455002_at | 19243 | protein tyrosine phosphatase 4a1;protein tyrosine phosphatase type IVA, member 1 | PTP4A1;Ptp4a1 | [RefSeq Summary:] The protein encoded by this gene belongs to a small class of prenylated protein tyrosine phosphatases (PTPs), which contains a PTP domain and a characteristic C-terminal prenylation motif. PTPs are cell signaling molecules that play regulatory roles in a variety of cellular processes. This tyrosine phosphatase is a nuclear protein, but may primarily associate with plasma membrane. The surface membrane association of this protein depends on its C-terminal prenylation. Overexpression of this gene in mammalian cells conferred a transformed phenotype, which implicated its role in the tumorigenesis. Studies in rat suggested that this gene may be an immediate-early gene in mitogen-stimulated cells. |
| 1455066_s_at | 236520 | RIKEN cDNA 9130229H14 gene | 9130229H14Rik | |
| 1455090_at | 26360 | angiopoietin-like 2 | ANGPTL2;Angptl2 | [RefSeq Summary:] Angiopoietins are members of the vascular endothelial growth factor family and the only known growth factors largely specific for vascular endothelium. Angiopoietin-1, angiopoietin-2, and angiopoietin-4 participate in the formation of blood vessels. ANGPTL2 protein is a secreted glycoprotein with homology to the angiopoietins and may exert a function on endothelial cells through autocrine or paracrine action. |
| 1455206_at | | | | |
| 1455534_s_at | 106326 | oxysterol binding protein-like 11 | OSBPL11;Osbpl11 | [RefSeq Summary:] This gene encodes a member of the oxysterol-binding protein (OSBP) family, a group of intracellular lipid receptors. Like most members, the encoded protein contains an N-terminal pleckstrin homology domain and a highly conserved C-terminal OSBP-like sterol-binding domain. |

| | | | | |
|---|---|---|---|---|
| 1455655_a_at | 230908 | TAR DNA binding protein | TARDBP;Tardbp | [RefSeq Summary:] HIV-1, the causative agent of acquired immunodeficiency syndrome (AIDS), contains an RNA genome that produces a chromosomally integrated DNA during the replicative cycle. Activation of HIV-1 gene expression by the transactivator Tat is dependent on an RNA regulatory element (TAR) located downstream of the transcription initiation site. The protein encoded by this gene is a transcriptional repressor that binds to chromosomally integrated TAR DNA and represses HIV-1 transcription. In addition, this protein regulates alternate splicing of the CFTR gene. A similar pseudogene is present on chromosome 20. |
| 1455789_x_at | 15481 | heat shock 70kDa protein 8;heat shock protein 8 | HSPA8;Hspa8 | [RefSeq Summary:] The product encoded by this gene belongs to the heat shock protein 70 family which contains both heat-inducible and constitutively expressed members. The latter are called heat-shock cognate proteins. This gene encodes a heat-shock cognate protein. This protein binds to nascent polypeptides to facilitate correct folding. It also functions as an ATPase in the disassembly of clathrin-coated vesicles during transport of membrane components through the cell. Two alternatively spliced variants have been characterized to date. |
| 1455820_x_at | 20778 | scavenger receptor class B, member 1 | SCARB1;Scarb1 | |
| 1455940_x_at | 83669 | WD repeat domain 6 | WDR6;Wdr6 | [RefSeq Summary:] This gene encodes a member of the WD repeat protein family. WD repeats are minimally conserved regions of approximately 40 amino acids typically bracketed by gly-his and trp-asp (GH-WD), which may facilitate formation of heterotrimeric or multiprotein complexes. Members of this family are involved in a variety of cellular processes, including cell cycle progression, signal transduction, apoptosis, and gene regulation. This gene is ubiquitously expressed in adult and fetal tissues. |

| | | | | |
|---|---|---|---|---|
| 1455961_at | 16483 | Kidney androgen-regulated protein;kidney androgen regulated protein | Kap | |
| 1456081_a_at | 78894 | acetoacetyl-CoA synthetase | AACS;Aacs | |
| 1456088_at | | | | |
| 1456125_a_at | 56455 | dynein, cytoplasmic, light chain 1 | Dnclc1 | |
| 1456341_a_at | 16601 | basic transcription element binding protein 1 | BTEB1;Bteb1 | [RefSeq Summary:] Basic transcription element binding protein 1 (BTEB1) is a transcription factor that binds to GC box elements located in the promoter. Binding of BTEB1 to a single GC box inhibits mRNA expression while BTEB1 binding to tandemly repeated GC box elements activates transcription. |
| 1456393_at | 18569 | programmed cell death 4;programmed cell death 4 (neoplastic transformation inhibitor) | PDCD4;Pdcd4 | [RefSeq Summary:] This gene encodes a protein localized to the nucleus in proliferating cells. Expression of this gene is modulated by cytokines in natural killer and T cells. The gene product is thought to play a role in apoptosis but the specific role has not yet been determined. Two transcripts encoding different isoforms have been identified. |
| 1460004_x_at | 58244 | syntaxin 6 | STX6;Stx6 | |
| 1460179_at | 15502 | DnaJ (Hsp40) homolog, subfamily A, member 1 | DNAJA1;Dnaja1 | |
| 1460235_at | 12492 | scavenger receptor class B, member 2 | SCARB2;Scarb2 | [RefSeq Summary:] The protein encoded by this gene is a type III glycoprotein that is located primarily in limiting membranes of lysosomes and endosomes. Studies of the similar protein in mice and rat suggested that this protein may participate in membrane transportation and the reorganization of endosomal/lysosomal compartment. Deficiency of the similar protein in mice was reported to impair cell membrane transport processes and cause pelvic junction obstruction, deafness, and peripheral neuropathy. |

| | | | | |
|---|---|---|---|---|
| 1460256_at | 12350 | carbonic anhydrase 1;carbonic anhydrase 13;carbonic anhydrase 14;carbonic anhydrase 15;carbonic anhydrase 2;carbonic anhydrase 3;carbonic anhydrase 4;carbonic anhydrase 5;carbonic anhydrase 5a, mitochondrial;carbonic anhydrase 5b, mitochondrial;carbonic anhydrase 6;carbonic anhydrase I;carbonic anhydrase II;carbonic anhydrase III, muscle specific;carbonic anhydrase | CA1;CA11;CA12;CA14;CA2;CA3;CA4;CA5A;CA5B;CA6;CA7;CA8;CA9;Ca2;Ca3;Ca4;Ca5a;Car1;Car13;Car14;Car15;Car2;Car3;Car4;Car5a;Car5b;Car6 | [RefSeq Summary:] Carbonic anhydrases (CAs) are a large family of zinc metalloenzymes that catalyze the reversible hydration of carbon dioxide. They participate in a variety of biological processes, including respiration, calcification, acid-base balanc |
| 1460314_s_at | 15077 | H3 histone, family 2 | Hist2h3c1 | |
| 1460342_s_at | 26936 | Rho interacting protein 3 | AA536749 | |

| | | | | |
|---|---|---|---|---|
| 1460409_at | 12894 | Carnitine palmitoyltransferase 2;carnitine palmitoyltransferase 1;carnitine palmitoyltransferase 1, liver;carnitine palmitoyltransferase 1A (liver);carnitine palmitoyltransferase 1B (muscle);carnitine palmitoyltransferase 1b;carnitine palmitoyltransferase 2;carnitine palmitoyltransferase II | CPT1A;CPT1B;CPT2;Cpt1a;Cpt1b;Cpt2 | [RefSeq Summary:] Carnitine palmitoyltransferase II precursor (CPT2) is a nuclear protein which is transported to the mitochondrial inner membrane. CPT2 together with carnitine palmitoyltransferase I oxidizes long-chain fatty acids in the mitochondria. |
| 1460432_a_at | 16341 | eukaryotic translation initiation factor 3, subunit 6;eukaryotic translation initiation factor 3, subunit 6 48kDa | EIF3S6;Eif3s6 | |
| 1460545_at | 230753 | RIKEN cDNA 9330151F09 gene;thyroid hormone receptor-associated protein, 150 kDa subunit | THRAP3;Thrap3 | |
| 1460555_at | 193385 | RIKEN cDNA 6330500D04 gene | 6330500D04Rik | |
| 1460662_at | 18628 | period homolog 1 (Drosophila);period homolog 2;period homolog 2 (Drosophila);period homolog 3 (Drosophila) | PER1;PER2;PER3;Per1;Per2;Per3 | [RefSeq Summary:] This gene is a member of the Period family of genes and is expressed in a circadian pattern in the suprachiasmatic nucleus, the primary circadian pacemaker in the mammalian brain. Genes in this family encode components of the circadian r |
